# Microwave-Assisted Synthesis of (Piperidin-1-yl)quinolin-3-yl)methylene)hydrazinecarbothioamides as Potent Inhibitors of Cholinesterases: A Biochemical and In Silico Approach

**DOI:** 10.3390/molecules26030656

**Published:** 2021-01-27

**Authors:** Rubina Munir, Muhammad Zia-ur-Rehman, Shahzad Murtaza, Sumera Zaib, Noman Javid, Sana Javaid Awan, Kiran Iftikhar, Muhammad Makshoof Athar, Imtiaz Khan

**Affiliations:** 1 Institute of Chemistry, University of the Punjab, Lahore 54590, Pakistan; noumanjavid@gmail.com (N.J.); atharmakshoof@gmail.com (M.M.A.); 2 Applied Chemistry Research Centre, PCSIR Laboratories Complex, Lahore 54600, Pakistan; 3 Department of Chemistry, University of Gujrat, Gujrat 50700, Pakistan; shahzad.murtaza@uog.edu.pk (S.M.); kiran.iffi@gmail.com (K.I.); 4 Department of Biochemistry, Faculty of Life Sciences, University of Central Punjab, Lahore 54590, Pakistan; sumera.biochem@gmail.com; 5 Department of Chemistry (C-Block), Forman Christian College, Ferozepur Road, Lahore 54600, Pakistan; 6 Institute of Molecular Biology and Biotechnology, The University of Lahore, Lahore 54000, Pakistan; sana.javaidawan@yahoo.com; 7 Department of Zoology, Kinnaird College for Women, Lahore 54000, Pakistan; 8 Department of Chemistry and Manchester Institute of Biotechnology, The University of Manchester, 131 Princess Street, Manchester M1 7DN, UK

**Keywords:** quinoline, piperidine, thiosemicarbazone, carbothioamide, Alzheimer’s disease, neurodegeneration, cholinesterases, molecular docking, ADME properties, HYDE assessment

## Abstract

Alzheimer’s disease (AD), a progressive neurodegenerative disorder, characterized by central cognitive dysfunction, memory loss, and intellectual decline poses a major public health problem affecting millions of people around the globe. Despite several clinically approved drugs and development of anti-Alzheimer’s heterocyclic structural leads, the treatment of AD requires safer hybrid therapeutics with characteristic structural and biochemical properties. In this endeavor, we herein report a microwave-assisted synthesis of a library of quinoline thiosemicarbazones endowed with a piperidine moiety, achieved via the condensation of 6/8-methyl-2-(piperidin-1-yl)quinoline-3-carbaldehydes and (un)substituted thiosemicarbazides. The target *N*-heterocyclic products were isolated in excellent yields. The structures of all the synthesized compounds were fully established using readily available spectroscopic techniques (FTIR, ^1^H- and ^13^C-NMR). Anti-Alzheimer potential of the synthesized heterocyclic compounds was evaluated using acetylcholinesterase (AChE) and butyrylcholinesterase (BChE) enzymes. The in vitro biochemical assay results revealed several compounds as potent inhibitors of both enzymes. Among them, five compounds exhibited IC_50_ values less than 20 μM. *N*-(3-chlorophenyl)-2-((8-methyl-2-(piperidin-1-yl)quinolin-3-yl)methylene)hydrazine carbothioamide emerged as the most potent dual inhibitor of AChE and BChE with IC_50_ values of 9.68 and 11.59 μM, respectively. Various informative structure–activity relationship (SAR) analyses were also concluded indicating the critical role of substitution pattern on the inhibitory efficacy of the tested derivatives. In vitro results were further validated through molecular docking analysis where interactive behavior of the potent inhibitors within the active pocket of enzymes was established. Quinoline thiosemicarbazones were also tested for their cytotoxicity using MTT assay against HepG2 cells. Among the 26 novel compounds, there were five cytotoxical and 18 showed proliferative properties.

## 1. Introduction

Alzheimer’s, also known as senile dementia, is a chronic neurodegenerative disease and is a global health problem due to its limited available treatments. The development mechanism of Alzheimer’s disease (AD) still remains elusive though multiple factors have been proposed for its induction out of which cholinergic hypothesis explains in the best manner [1]. According to the hypothesis, the diminution of acetylcholine level causes cognitive deficit and memory loss. The inhibition of acetylcholinesterase (AChE; EC 3.1.1.7) and butyrylcholinesterase (BChE; EC 3.1.1.8) is supposed to be beneficial for the treatment of AD [2]. The AChE enzyme hydrolyzes the neurotransmitter (acetylcholine) to acetic acid and choline resulting in reduction of its levels that is the main cause of AD [3] while BChE hydrolyzes butyrylcholine (BuCh). Histologically, AChE is mostly of neuronal origin, while BChE is primarily present in the blood and glial cells [4,5,6,7,8]. To prevent the cholinesterase enzyme from hydrolyzing the neurotransmitters, several cholinesterase inhibitors such as donepezil, rivastigimine, tacrine, ensaculin, and galantamine have been designed, and used for the treatment of cognitive dysfunction and memory loss of AD patients. However, several adverse side effects such as nausea, vomiting, decreased appetite, weight loss, and hepatotoxicity associated with these drugs necessitate the development of new cholinesterase inhibitors for the effective treatment of AD [9,10,11].

**Figure 1:**
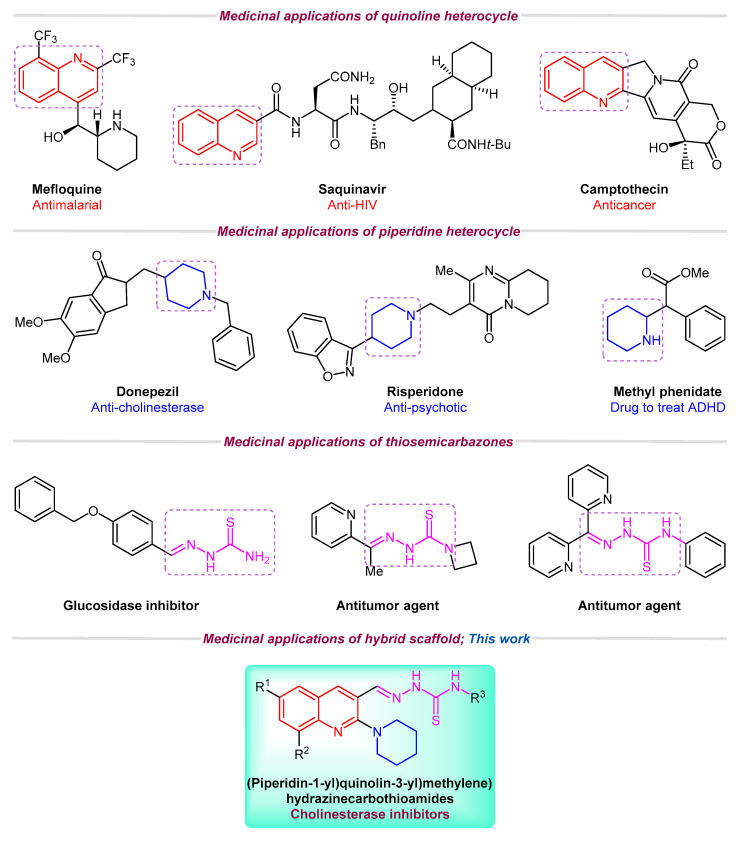
Medicinal importance of quinoline, piperidine, and thiosemicarbazone motifs and rationale of current study.

Among nitrogen-containing heterocycles, quinoline is considered as an important scaffold due to its marvelous pharmacological potential [12,13]. Several commercial drugs such as Primaquine^®^, Plasmoquine^®^, Mefloquine^®^, Chloroquine^®^, OSI-930, and Saquinavir^®^ incorporate quinoline pharmacophore [14,15]. A range of quinoline derivatives have been reported for their analgesic [16], antifungal [17], antibacterial [18], antioxidant [19], anticancer [20,21], anti-inflammatory [22], antiviral [23,24], anti-Alzheimer [25], cytotoxic [26], antileishmanial [27,28], and anti-hypertensive activities [29,30]. Similarly, piperidine ring is also prevalent in numerous naturally occurring alkaloids [31]. According to FDA Orange Book, piperidine ring falls among the top classified scaffolds in the list of 100 most exploited simple ring systems in drug design and synthesis [32]. Piperidine derivatives also demonstrate various biological functions such as antimalarial [33], anticonvulsant [34], anticancer [35], and antidepressant [36] activities. Crizotinib^®^ [37], Donepezil^®^ [38], Risperidone^®^ [39], and Methyl phenidate^®^ [40] are the well-known examples of piperidine-based drugs (Figure 1). Likewise, thiosemicarbazones are emerging scaffolds of medicinal interest and their antimicrobial [41,42], anti-inflammatory [43], antioxidant [44,45], anticancer [46,47,48], anti-Alzheimer [49], and alpha-glucosidase inhibitory [50] activities have been reported in recent years.

In view of our continued interest in the development of heterocyclic leads for the treatment of AD [51,52,53,54,55,56], and emerging trend in the exploration of hybrid molecules featuring more than one biologically potent moiety [57,58,59], we herein report the successful integration of piperidine and thiosemicarbazone pharmacophores with quinoline scaffold to develop a library of new hybrid heterocyclic molecules for the treatment of Alzheimer’s disease (AD). The target compounds were synthesized successfully under microwave irradiation conditions and were evaluated for their AChE and BChE inhibitory potential. Quinoline thiosemicarbazones were also tested for their cytotoxicity using MTT assay against HepG2 cells. Moreover, the binding affinities of the potent inhibitors in the active site of both enzymes were elucidated using molecular docking approach.

## 2. Results and Discussion

### 2.1. Synthetic Chemistry

**Scheme 1:**
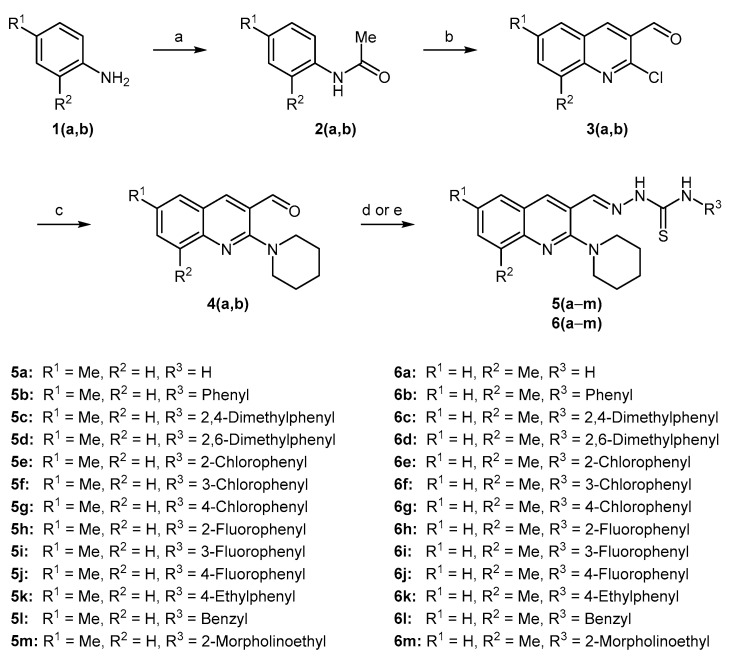
Synthesis of 2-((6/8-methyl-2-(piperidin-1-yl)quinolin-3-yl)methylene)hydrazinecarbothioamides. *Reagents and Conditions*: (**a**) glacial acetic acid, *o*-phosphoric acid, reflux, 6–7 h, 80–85%; (**b**) DMF, POCl_3_, 0→80 °C, 16–18 h, 65–75%; (**c**) piperidine, PEG-400, CTAB, 135 °C, 2.5 h, 97–98%; (**d**) substituted thiosemicarbazide, glacial acetic acid, EtOH, MWI, 3–5 min; (**e**) substituted thiosemicarbazide, glacial acetic acid, EtOH, reflux, 0.5–2 h.

A library of new piperidine containing quinolinyl thiosemicarbazones **5(a**–**m)** and **6(a**–**m)** was synthesized using a facile synthetic approach as illustrated in Scheme 1. Acetylation of the commercial anilines **1(a,b)** produced acetanilides **2(a,b)** which underwent Vilsmeier–Haack formylation using dimethylformamide and phosphoryl chloride affording 2-chloroquinoline-3-carbaldehydes **3(a,b**) in 65–75% yield [60]. Subsequently, cetyltrimethylammonium bromide (CTAB) catalyzed nucleophilic aromatic substitution of **3(a,b)** with piperidine in polyethylene glycol-400 (PEG-400) produced 2-(piperidin-1-yl)quinoline-3-carbaldehydes **4(a,b)** in 97–98% yield [61]. Finally, microwave-assisted condensation of **4(a,b)** with a range of thiosemicarbazides afforded the desired hybrid compounds **5(a**–**m)** and **6(a**–**m)** in excellent yields in 3–5 min (Table 1). The target compounds were also prepared using the conventional approach, however, the isolated yields were lower compared to microwave-assisted methodology. The comparative yields are given in Table 1. Two-point structural diversity was introduced in the target compounds. Quinoline pharmacophore inherits 6- and 8-substitutions (R^1^ and R^2^) from commercially available anilines whereas electron-rich and electron-deficient groups were introduced at the aromatic ring (R^3^) in the thiosemicarbazides. Benzyl and morpholinoethyl were also found as successful substituents as R^3^ on the thiosemicarbazide moiety.

### 2.2. Spectroscopic Characterization

**Figure 2:**
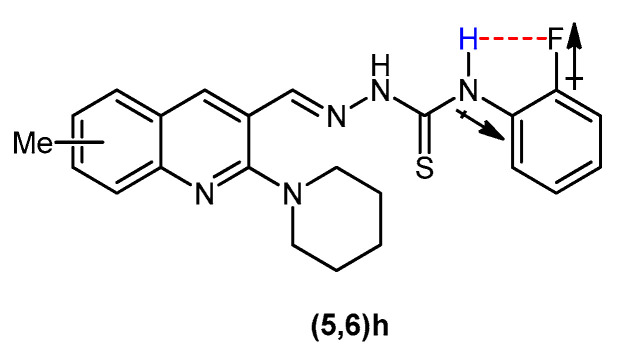
Deshielding of Ar-NH in compounds **5h** and **6h**.

The condensation reaction between a carbaldehyde and a thiosemicarbazide was confirmed by the appearance of a singlet around 8.77–9.07 ppm attributable to imine (N=CH) proton. Two downfield singlets of secondary thioamide protons endorsed the formation of desired product. The =N-NH proton, being more deshielded exhibited signal around 11.74–12.27 ppm, while C-NH proton was observed around 9.17–10.28 ppm. An exception was observed in the spectral data of compounds **5a** and **6a** regarding C-NH signal. Two –NH_2_ protons appeared as two discrete broad singlets at 8.14 and 8.33–8.37 ppm. This is attributed to the existence of thiosemicarbazone in different stereochemical forms and is in accordance with the previous theoretical and stereochemical studies of carbothioamides [62,63,64]. In the ^1^H NMR spectra of compounds **5h** and **6h**, the C-NH proton signal appeared downfield due to the electron-withdrawing inductive effect of fluoro group attached to the phenyl ring ortho to thioamide functionality. Along with electron-withdrawing inductive effect, the electrostatic attraction between the electronegative fluorine and the electropositive hydrogen may also develop resulting in the downfield shifting of the peak (Figure 2).

**Figure 3:**
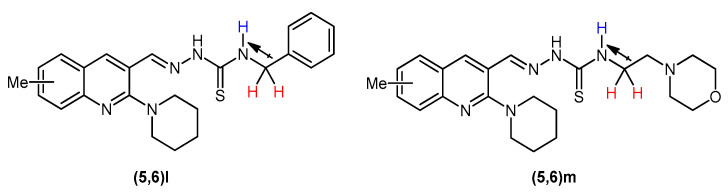
Upfield shifting and splitting of –NH signal in **5(l,m)** and **6(l,m)**.

Another distinct pattern was observed in compounds **5(l,m)** and **6(l,m)** in which the C-NH signal emerged comparatively upfield as a triplet (*J* = 6.0 Hz) instead of a singlet. The upfield chemical shift can be ascribed to the aliphatic carbon atom succeeding –NH that shields this proton as compared to the C-NH of the rest of the compounds in this series. The triplet spin multiplicity of –NH peak is due to the coupling of this proton with –CH_2_ protons in its immediate vicinity (Figure 3).

The peaks of aromatic protons, depending upon their chemical environment, were seen in a range of 7.05–8.37 ppm. Among aromatic protons, the singlet for H_4_ of quinoline ring showed the highest chemical shift around 8.29–8.37 ppm. In the spectral data of compounds **5e**, **5h**, and **6h**, the aromatic protons appeared downfield referring to the presence of electronegative fluoro and chloro group at ortho position of thioamide functionality. In the spectral data of compounds **(5,6)m**, the six methylene protons referring to four –OCH_2_ protons of morpholine ring and two protons of thioamide NH-CH_2_ emerged as a multiplet around 3.89–3.97 ppm while the six N–CH_2_ protons connected to nitrogen of morpholine ring gave a multiplet peak around 2.90–2.70 ppm. In compounds **(5,6)l**, the methylene of benzyl appeared as a triplet at 4.90 ppm, the higher chemical shift and splitting of signal owing to the vicinity of the –NH (Figure 3). Likewise, the piperidine ring protons appeared as two multiplet peaks near 1.70 and 3.25 ppm, respectively.

^13^C NMR spectra further confirmed the newly formed structures by showing the peak for C=S carbon as the most deshielded signal around 175.5–178.5 ppm. The peaks of the aromatic and imine carbon atoms appeared between 112 and 163 ppm. The elemental analyses were also in good agreement with the proposed structures.

Furthermore, the phenomenon of geometric as well as conformational isomerism was observed in the ^1^H NMR as well as ^13^C NMR of the derivatives in less polar solvents like chloroform. The compounds **5(a–m)** and **6(a–m)** could exist in either *E* or *Z* isomeric form because of azomethine (-CH=N-) linkage while C-N bond rotation may give rise to rotamers. Compound **5g** was selected as a test compound and its NMR spectral data were recorded in CDCl_3_ as well as DMSO. The spectra in CDCl_3_ showed additional peaks referring to various isomers. However, ^1^H NMR spectra in DMSO exhibited no additional peaks regarding NH or N=CH indicating the presence of a single isomeric form (see Supplementary Materials).

### 2.3. In Vitro Cholinesterase Inhibition and Structure–Activity Relationship Analyses

**Figure 4:**
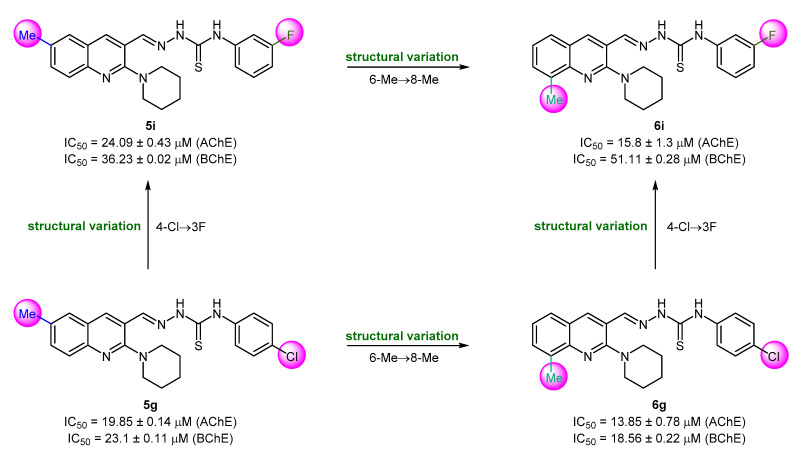
Structure–activity relationship analysis of compounds **5g**, **5i**, **6g**, and **6i**.

The newly synthesized (piperidin-1-yl)quinolin-3-yl)methylene)hydrazinecarbothioamides were screened for the identification of robust and potent inhibitors of cholinesterase (AChE and BChE) enzymes. The results presented in Table 1 indicate the potential of this hybrid scaffold to serve as a template for future investigations while making some key structural variations to obtain the cholinesterase inhibitors of desirable impact. Herein, we demonstrate some key structure–activity relationships based on the in vitro biochemical assay results. The effect of methyl substitution at the 6- (R^1^) and 8-position (R^2^) of quinoline was studied in the first instance while keeping the thiosemicarbazone chain unchanged. Donepezil was employed as a standard drug. Several compounds showed IC_50_ values less than 20 μM. 2-((6-Methyl-2-(piperidin-1-yl)quinolin-3-yl)methylene)hydrazinecarbothioamides showed moderate results with relatively higher IC_50_ values as compared to their (8-methyl-2-(piperidin-1-yl)quinolin-3-yl) analogues. Only compound **5g** demonstrated good inhibitory efficacy with IC_50_ value of 19.85 ± 0.14 μM against AChE while rest of the compounds in this series were less active (IC_50_ > 20 μM). Compound **5g** incorporates a 4-chlorophenyl ring as a R^3^ substituent on the thiosemicarbazone moiety. A dual and enhanced inhibition was noticed when the position of methyl substituent was switched from 6 to 8 on the quinoline ring (compound **6g**). Replacing the 4-chloro substituent with a more electronegative 3-fluoro group at the aryl ring (R^3^) produced diminished inhibition (**5i**), however, a combination of 3-fluoro and 8-methyl substituents produced the lead selective inhibitor of AChE (**6i**; IC_50_ = 15.8 ± 1.3 μM) (Figure 4).

**Figure 5:**
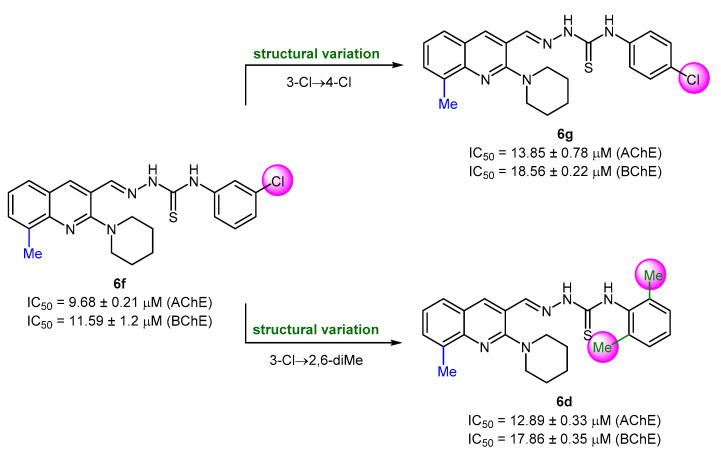
Structure–activity relationship analysis of dual inhibitors **6d**, **6f**, and **6g**.

The compounds with 8-methyl substitution exhibited good results with three compounds (**6d**, **6f**, and **6g**) acting as dual inhibitors with IC_50_ < 20 μM against AChE and BChE. Among them, **6f** emerged as the most potent inhibitor against both enzymes with IC_50_ values of 9.68 ± 0.21 μM (AChE) and 11.59 ± 1.2 μM (BChE). Compound **6f** incorporates a 3-chlorophenyl ring as R^3^ substituent. The introduction of a di-substitution (2,6-dimethyl) caused a detrimental effect on the inhibitory potency (compound **6d**). Similar trend was also noticed when chlorine substituent was moved to position 4 at the phenyl ring (compound **6g**) (Figure 5). The effect on the anti-cholinergic activity by different substituents such as chloro, fluoro, and methyl on the phenyl group of hydrazine carbothioamide moiety was also evaluated [65]. Anti-cholinesterase potential was predominant for the meta substituted chloro and fluoro molecules when 8-position of quinoline is occupied by a methyl substituent.

In case of dimethyl substituted inhibitors, 2,6-disubstituted analogues showed remarkably good results as compared to compounds bearing 2,4-dimethyl substitution on the aromatic ring (R^3^), no matter whether the quinoline ring is substituted at 6- or 8-position. Compounds bearing a benzyl substituent as R^3^ were ranked among the least active inhibitors. Moreover, morpholine substituted compound **6m** showed better inhibitory activity than **5m**. 

### 2.4. Molecular Docking Studies

**Figure 6:**
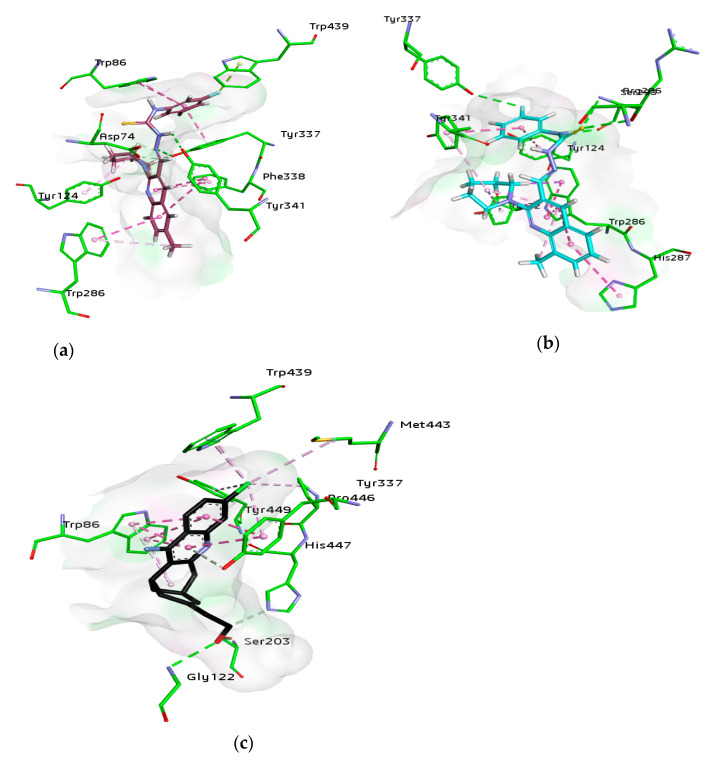
3D binding modes of **5i** (**a**), **6i** (**b**), and **Huprine W** (**c**) with the amino acid residue of AChE, hydrogen bonding is shown by green dashed line, hydrophobic interactions are shown by light purple color, dark pink dashed lines show π-T shaped, light pink color shows π-π stacked interactions, and purple color dashed lines show π-sigma type interactions.

**Figure 7:**
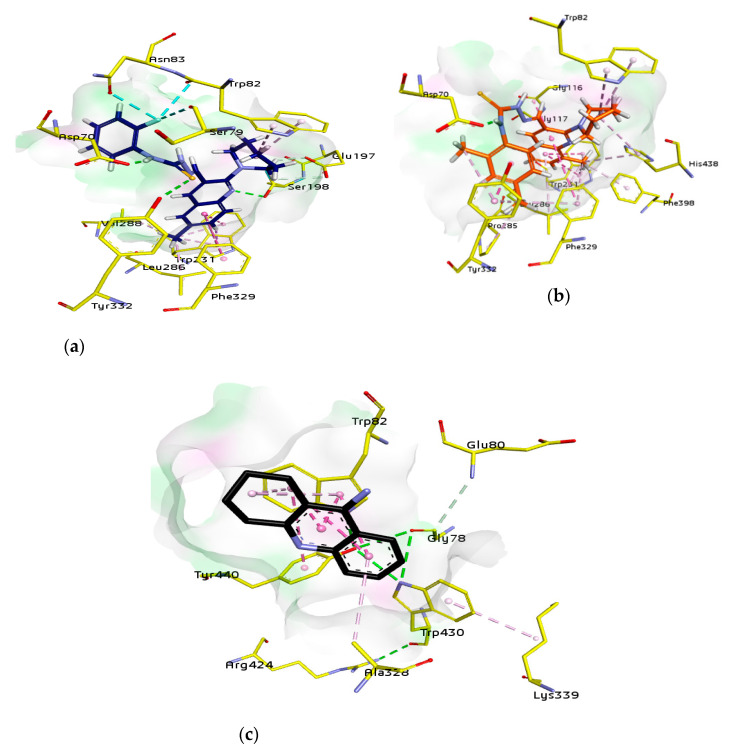
3D binding modes of **5h** (**a**), **6c** (**b**), and **tacrine** (**c**) with the amino acid residue of BChE, hydrogen bonding is shown by green dashed line, hydrophobic interactions are shown by light purple color, dark pink dashed lines show π-T shaped, light pink color shows π-π stacked interactions, and purple color dashed lines show π-sigma type interactions.

**Figure 8:**
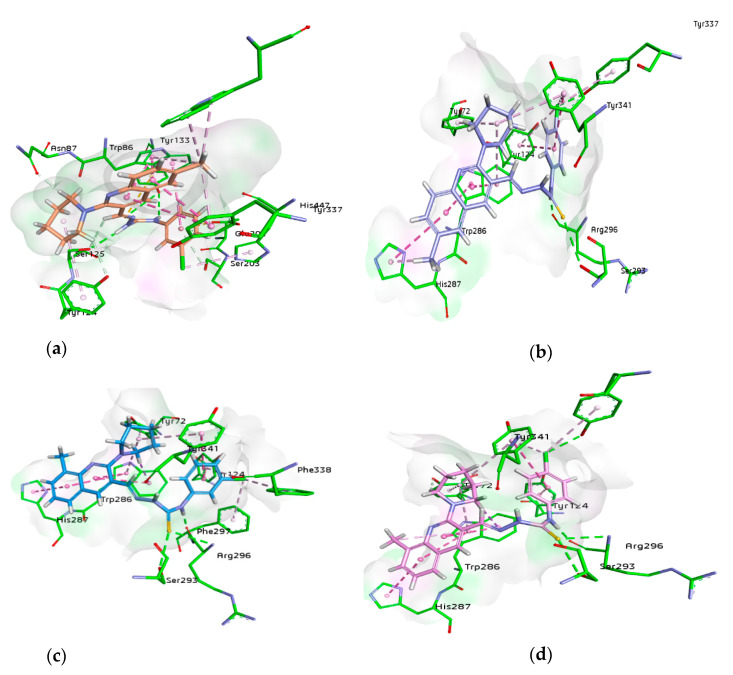
3D binding modes of **5f** (**a**), **5g** (**b**), **6f** (**c**), and **6g** (**d**) with the amino acid residue of AChE, hydrogen bonding is shown by green dashed line, hydrophobic interactions are shown by light purple color, dark pink dashed lines show π-T shaped, light pink color shows π-π stacked interactions, and purple color dashed lines show π-sigma type interactions.

**Figure 9:**
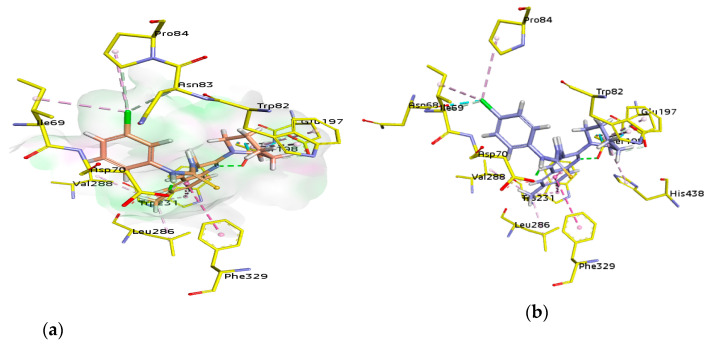

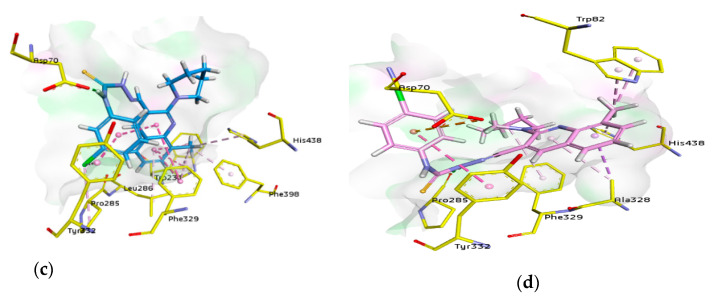
3D binding modes of **5f** (**a**), **5g** (**b**), **6f** (**c**), and **6g** (**d**) with the amino acid residue of BChE, hydrogen bonding is shown by green dashed line, hydrophobic interactions are shown by light purple color, dark pink dashed lines show π-T shaped, light pink color shows π-π stacked interactions, and purple color dashed lines show π-sigma type interactions.

For docking studies, X-ray structures of human AChE (PDB ID: 4BDT) [66] and BChE (PDB ID: 4BDS) [66] were selected as templates, because structures of electric eel AChE were available at low crystallographic resolutions (>4 Å) and a structure of equine BChE was not available. Molecular docking analysis of potent compounds was performed against AChE and BChE for the identification of possible binding modes. The orientation of the most potent and selective compounds **5i** and **6i** and crystallographic inhibitor **huprine W** were shown in the active site of AChE (Figure 6), whereas, the binding modes of **5h** and **6c**, the most potent and selective compounds and cognate ligand **tacrine** were shown in the active site of BChE (Figure 7). The orientation of dual inhibitors (**5f**, **5g**, **6f**, and **6g**) were shown in Figure 8 and Figure 9.

#### 2.4.1. Molecular Docking Studies of Acetylcholinesterase (AChE)

The active pocket of AChE was surrounded by amino acid residues Tyr124, Gly122, Tyr337, Phe297, Leu289, Val340, Ser298, Arg296, Phe338, Trp286, Ser125, Leu76, Tyr341, Tyr72, Ala204, Ser203, and His447. The hydrogen bonds and π-π interactions were formed by the potent inhibitor **5i** as well as by **huprine W**, as reported previously [66]. The cognate ligand (**huprine W**) showed two conventional hydrogen bonds with Ser203 (2.33 Å) and Gly122 (2.96 Å) and multiple π-π stacking (4.00, 4.41, 5.30, and 3.69 Å) with Trp86. Additionally, 2-alkyl linkages (4.18 and 4.87 Å) and an alkyl linkage (4.52 Å) were seen with Pro446. Moreover, Tyr337 formed two π-π stacked bonds (3.54 and 4.47 Å), one π-alkyl bond (4.45 Å), and π-donor hydrogen bond (4.01 Å) with **huprine W**. Other interactions like one π-alkyl with Tyr449 (5.38 Å), one π-alkyl with Met443 (4.89 Å) and two π-alkyl with Trp439 (3.80 and 3.46 Å) and a carbon-hydrogen bond (3.53 Å) with His447 were observed. The most potent compound **5i** displayed several important interactions with amino acids in the active pocket like π-alkyl bond (5.14 Å) with methyl substituent and π-π T shaped (5.06 Å) with phenyl ring of Trp286. Moreover, two π-π T shaped with phenyl ring (4.66 and 5.42 Å) and a conventional hydrogen bond with carbothioamide (2.32 Å) by Tyr341 and a carbon hydrogen bond (2.42 Å) with Asp74 were noted. Likewise, the compound showed π-π T shaped interactions (5.46 Å) with phenyl ring Phe338, π-lone pair (2.95 Å) with 3-fluoro substituent by Trp439 and two π-π stacked (4.98 and 4.95 Å) formed by fluorophenyl ring with Trp86. The amino acid Tyr337 showed π-π T shaped (3.92 Å) with fluorophenyl ring and a conventional hydrogen bond (2.37 Å) with carbothioamide along with a π-alkyl bond (4.64 Å) with piperidine ring by Tyr124 (Figure 6). Another potent and selective compound **6i** docked inside the AChE represented several important interactions including π-π stacking (5.67 Å) with methylquinoline ring by His287, π-π T shaped (5.51 Å) with fluorophenyl ring by Tyr124 and a conventional hydrogen bond (3.56 Å) with fluorine substituent by Tyr337. Other interactions are π-π T shaped (4.75 Å) with fluorophenyl ring and π-alkyl (5.24 Å) with piperidine ring by Tyr341, conventional hydrogen bonds (3.61 and 2.95 Å, respectively) by Arg296 and Ser293 with sulfur of thiocarbonyl moiety. Compound **6i** also formed three π-π stacked interactions with methylquinoline ring (4.30, 5.24, and 4.86 Å), a π-sigma bond (2.74 Å), and two π-alkyl bonds with methyl (5.25 Å) and piperidine (5.20 Å) by Trp286 and a π-alkyl (5.07 Å) with piperidine and one carbon hydrogen bond (2.41 Å) by Tyr72 (Figure 6).

#### 2.4.2. Molecular Docking Studies of Butyrylcholinesterase (BChE)

The active pocket of BChE was surrounded by amino acid residues Tyr332, Ser198, Met437, Glu197, Ala328, Asp70, Trp430, Phe73, Gly121, Thr120, Trp82, Ile442, Gly115, Gly117, Trp231, Ser79, His438, Tyr332, Tyr440, and Val331 [53]. The detailed analysis of **tacrine** (tetrahydroacridin-9-amine) suggested the presence of two π-alkyl (4.32 and 4.97 Å) and four π-π stacked bonds (4.25, 4.23, 5.61, and 3.59 Å) with Trp82 and a π-alkyl linkage (4.56 Å) with Ala328. All the interactions were presented by amino acid residues Trp82 and Ala328 as shown in Figure 7. 

The potent and selective compound **5h** formed a conventional hydrogen bond (2.49 Å) with quinoline ring and a carbon-hydrogen bond (2.81 Å) with piperidine ring by Ser198. Similarly, a conventional hydrogen bond (3.78 Å) by Tyr332 with sulfur atom and same bond (1.84 Å) by Asp70 with NH of carbothioamide moiety were observed. A halogen bond (2.90 Å) and a conventional hydrogen bond (3.37) with 2-fluoro group by Asn83, and a halogen bond (3.66 Å) with 2-fluoro group by Ser79 were noted. Moreover, two π-alkyl linkage with piperidine (4.41 and 5.01 Å) and a halogen bond with 2-fluoro group (3.28 Å) by Trp82, and two carbon-hydrogen bonds (2.35 and 3.06 Å) with piperidine ring by Glu197 and a π-π T shaped (5.63 Å) with quinolone ring by Phe329 were noticed. Additionally, two π-alkyl linkages (4.01 and 4.73 Å) with 8-methyl and two π-π T shaped linkages (5.03 and 4.73 Å) with quinoline ring in addition to a π-sigma bond (2.67 Å) with the same by Trp231 were formed. The alkyl linkages by Leu286 (4.07 Å) and Val288 (5.07 Å) with 8-methyl substituent were observed by the same compound (Figure 7). 

Another compound **6c** produced two π-alkyl linkages via piperidine ring (4.96 and 5.10 Å) by Trp82, a conventional hydrogen bond (1.81 Å) with carbothioamide moiety by Asp70, and a π-π stacked (3.60 Å) interaction with 2,4-dimethyphenyl ring and two π-alkyl (4.70 Å) interactions with 2- and 4-methyphenyl (4.67 and 3.79 Å, respectively) by Tyr332. Moreover, amide-π stacked interactions (4.01 Å) were noticed by Gly116 and a π-donor (3.71 Å) by Gly117 with quinoline ring, while, a π-alkyl (4.19 Å) with 4-methyphenyl and another π-alkyl (5.26 Å) with 8-methyl group and a π-π T shaped linkage with quinoline ring (5.64 Å) were formed by Phe329. An additional alkyl linkage (4.70 Å) with 4-methyphenyl by Pro285, two π-alkyl linkages via piperidine ring (4.52 Å), and another by 8-methyl of quinoline ring (4.81 Å) by His438 and a π-alkyl linkage by 8-methyl of quinoline ring (4.07 Å) by Phe398 were observed. Two π-alkyl linkages were noticed by 8-methyl of quinoline ring (4.06 and 4.08 Å) and two π-π T shaped linkages with quinoline ring (5.39 and 5.31 Å) by Trp231, while, two π-alkyl linkages by 8-methyl of quinoline ring (5.46, 5.40 Å) by Leu286 (Figure 7). 

#### 2.4.3. Dual Inhibitors of AChE and BChE

Compound **5f** being the dual inhibitor of both enzymes showed some interesting interactions inside the active site of AChE that includes two π-π stacked bonds (4.42 and 5.67 Å) with methylquinoline and a π-alkyl linkage (4 Å) with methyl group through Tyr337, π-alkyl linkage (5.38 Å), and a carbon H bond (2.61 Å) with piperidine ring by Tyr124 and π-alkyl bonds with His447 (4.51 Å) and Ser203 (3.84 Å) by 3-chloro substituent. Additionally, a carbon-hydrogen bond (2.01 Å) with piperidine ring by Asn87, π-anion (3.99 Å) with a 3-chloro group by Glu202, π-sulfur (5.59 Å) with sulfur atom and two conventional hydrogen bonds with sulfur (3.64 Å) and NH (2.76 Å) by Tyr133 and a conventional hydrogen bond (2.30 Å) and carbon-hydrogen bond (2.84 Å) with carbothioamide moiety by Ser125. However, a π-π T shaped with 3-chloro group (5.15 Å) and two π-π stacked bonds with methylquinoline (4.15 and 4.29 Å) and additionally a π-alkyl linkage with methyl group (5.20 Å) by Trp86 and two π-alkyl bonds (4.06 and 4.08 Å) with methyl group by Trp439 were formed (Figure 8). 

Similarly, compound **5g** showed a conventional hydrogen bond (2.58 Å) and π-alkyl linkage (4.80 Å) with 4-chloro group by Tyr337, and π-alkyl linkage (5.01 Å) with piperidine, π-π T shaped (4.69 Å) with chlorophenyl ring and π-alkyl (4.35 Å) with 4-chloro group by Tyr341. Other important amino acids contributed significantly by different types of interactions like three π-π stacked bonds with methylquinoline (4.89, 4.77, and 4.03 Å) and a π-alkyl bond (5.49 Å) with piperidine by Trp286, a carbon H bond (4.72 Å) and π-alkyl linkage (5.17 Å) with piperidine by Tyr72 and π-π T shaped (5.32 Å) with chlorophenyl ring and π-alkyl (5.42 Å) with 4-chloro group by Tyr124. Moreover, a conventional hydrogen bond (1.94 Å) with carbothioamide moiety by Arg296 and same bond (3.34 Å) with sulfur atom by Ser293 and a π-π stacked bond (5.70 Å) with methylquinoline and π-alkyl bonds (4.19 Å) with methyl group by His287 were noticed (Figure 8). 

Another dual and the most potent inhibitor **6f**, formed one π-π stacked (5.65 Å) interaction with 8-methylquinoline ring by His287, one π-π T shaped (5.60 Å) and a π-alkyl (4.98 Å) with 4-chlorophenyl ring by Tyr124, one π-π T shaped (4.64 Å) with 3-chlorophenyl ring and one π-alkyl (5.32 Å) with piperidine ring by Tyr341 and π-alkyl linkage (4.98 Å) with chloro group by Phe338. More interactions like two conventional hydrogen bonds with sulfur (3.58 Å) and NH (1.71 Å) of carbothioamide by Arg296, a hydrogen bond (2.95 Å) with sulfur of thiocarbonyl by Ser293, and a π-alkyl linkage (4.40 Å) with chloro group by Phe297 were noted. Three π-π stacked interactions (4.37, 5.33, and 4.95 Å) were observed with methyl quinoline ring, a π-sigma bond (2.74 Å), and a π-alkyl bond with piperidine (5.23 Å) by Trp286 in addition to a π-alkyl (5.06 Å) with piperidine ring by Tyr72 (Figure 8). 

The next dual inhibitor was **6g** that formed π-π stacked (5.67 Å) interaction with 8-methylquinoline ring by His287, a π-π T shaped (5.48 Å) with 4-chlorophenyl ring by Tyr124, and a conventional hydrogen bond (2.71 Å) and π-alkyl bond (4.83 Å) with chloro group by Tyr337. Other interactions like π-π T shaped (4.75 Å) with 4-chlorophenyl ring and one π-alkyl (4.06 Å) with chloro group and another with piperidine ring (5.34 Å) by Tyr341 were also observed. Additionally, two conventional hydrogen bonds (3.60 Å) with sulfur and another with NH (1.68 Å) of the carbothioamide by Arg296 and a conventional hydrogen bond (2.94 Å) with sulfur of the carbothioamide by Ser293 were noticed. Moreover, three π-π stacked interactions (4.28, 5.24, and 4.85 Å) with methylquinoline ring, a π-sigma bond (2.70 Å), and two π-alkyl bonds with methyl (5.24 Å) and piperidine ring (5.17 Å) by Trp286 and a π-alkyl (5.05 Å) and one carbon-hydrogen bond (2.38 Å) with piperidine ring by Tyr72 were noticed (Figure 8). These multiple interactions especially π-π and strong hydrogen bonds of potent compounds binding in the center of the active pocket may be the possible reason for the inhibitory profile of these derivatives.

Similarly, compound **5f** being a dual inhibitor showed several interactions inside the active pocket of BChE such as a conventional hydrogen bond (2.52 Å) with nitrogen atom of quinoline ring by Ser198, a π-π T shaped linkage (5.04 Å) with 6-methyl quinoline ring by Phe329, two more π-π T shaped linkages (4.74 and 5.05 Å) with 6-methyl quinoline ring, π-sigma bond with the same ring (2.69 Å), and π-alkyl bonds (4.04 and 4.74 Å) with 6-methyl of quinoline ring by Trp231. One alkyl bond (4.08 Å) by Leu286 and another by Val288 (5.05 Å) with 6-methyl of quinoline ring were noted. Asp70 formed a conventional hydrogen bond (1.82 Å) with NH of carbothioamide moiety. Moreover, an alkyl bond (4.36 Å) with 3-chloro of phenyl by Pro84, a carbon halogen bond (3.27 Å) with 3-chloro of phenyl by Asn83, an alkyl bond (4.54 Å) with 3-chloro of phenyl by Ile69 and two more π-alkyl linkages (5.00 and 4.40 Å) with piperidine ring by Trp82 were noticed (Figure 9).

Compound **5g** showed an alkyl linkage (4.97 Å) by Val288 and Leu286 (4.12 Å) with 6-methyl quinoline ring, additionally a π-donor bond (3.58 Å) with 4-chlorophenyl ring and a conventional H-bond (1.91 Å) with NH of carbothioamide by Asp70. A halogen bond (2.30 Å) was shown by 4-chloro with Asn68. Moreover, two π-alkyl linkages (4.97 and 4.31 Å) with 6-methyl and two π-π T shaped linkages (5.13 and 4.88 Å) with 6-methyl quinoline ring, in addition to π-sigma bond with the same ring (2.75 Å) were formed by Trp231. Similarly, π-π T shaped linkage (5.57 Å) with 6-methylquinoline ring by Phe329 was noted. A conventional hydrogen bond (2.48 Å) with quinoline ring and a carbon-hydrogen bond (2.80 Å) with piperidine ring by Ser198 were observed. Two carbon-hydrogen bonds (2.24 and 3.00 Å) by Glu197 and two π-alkyl linkage (5.03 and 4.38 Å) by Trp82 with piperidine ring and a π-alkyl linkage (5.30 Å) with piperidine ring by His438, an alkyl bond (4.76 Å) with 4-chloro by Pro84, carbon-hydrogen bond (3.21 Å) with 4-chloro and an alkyl bond (4.12 Å) with the same by Ile69 were also formed (Figure 9).

Compound **6f** formed π-alkyl (4.63 Å) with methyl substituent by His438, two π-alkyl (4.01 and 4.06 Å) with methyl and two π-π T shaped (5.46 and 5.71 Å) with quinoline ring by Trp231, π-alkyl with methyl (3.91 Å) by Phe398, π-alkyl (4.43 Å) with quinoline ring by Leu286, π-alkyl (5.39 Å) with 8-methyl of quinoline ring and two π-π T shaped linkages (4.68 and 5.37 Å) with quinoline ring by Phe329 were observed. Moreover, a conventional hydrogen bond (1.97 Å) with carbothioamide by Asp70, an alkyl linkage (4.59 Å) with chloro group by Pro285, and a π-π stacked (3.82 Å) interaction with 3-chlorophenyl ring and a π-alkyl (4.24 Å) with chloro group by Tyr332 were also present (Figure 9).

Compound **6g** showed π-alkyl bond (5.48 Å) by His438 and two π-alkyl bonds (3.75 and 3.96 Å) by Trp82 with 8-methyl substituent. A π-alkyl bond (5.99 Å) with piperidine ring by Phe329, a conventional hydrogen bond (2.02 Å) with carbothioamide by Pro285, a π-anion (4.46 Å) with 4-chlorophenyl by Asp70, a π-π stacked (5.39 Å) interaction with 4-chlorophenyl by Tyr332 and a π-sigma (3.88 Å) and a π-alkyl (4.40 Å) with 8-methylquinoline ring by Ala328 were also formed (Figure 9).

All the compounds were found to bind deep inside the active pocket of BChE and were involved in forming π-sigma, π-π stacked, π-anion interactions with different amino acid residues. The docking studies were in parallel to the in vitro results. Taken together, the results presented herein showed that the novel 6- and 8-methyl-2-(piperidin-1-yl)quinolin-3-yl)methylene)hydrazine-1-carbothioamide derivatives are promising inhibitors of cholinesterases.

### 2.5. HYDE Assessment of Selective Compounds against Cholinesterases (AChE and BChE)

HYDE visual affinity of all the ligands was carried out using LeadIT [67] software for top 30 ranked docked conformers within the active site of the human AChE and BChE. The binding energy and docking score by FlexX for the all the synthetic derivatives (6- and 8-methyl-2-(piperidin-1-yl)quinolin-3-yl)methylene)hydrazine-1-carbothioamides) are given in Table 2. The FlexX docking score depicted that the selective derivatives have lower energy scores as compared to nonselective inhibitors. Moreover, the binding free energies ΔG given in Table 2 showed that the potent inhibitors exhibited higher affinity towards the respective target (AChE and BChE). The docking scores of all the compounds revealed the similar pattern as was suggested by in vitro analysis. The compounds that were inactive exhibited lower docking scores while active and potent inhibitors showed significant docking scores with the significant free binding energy values.

### 2.6. ADME Properties

ADME properties predict the impact of therapeutic compounds to access the target considering some parameters. These properties include physicochemical properties, lipophilicity, water solubility, pharmacokinetics, drug-likeness, and medicinal chemistry. These properties were evaluated using several prediction tools [68,69,70]. The properties help in determining the drug-likeness of compounds being used for drug discovery and development by sorting out new druggable candidates that are safer and follow the effective rules used for determination of these parameters. Table 3 represents some important ADME properties compiled using web service and its underlying methodologies (SwissADME) [68]. The properties suggested that our selective derivatives are safer to use as a drug and have high probability of gastrointestinal absorption. Moreover, the compounds showed no violation for Lipinski, Veber, and Egan rules and these filters originate from analyses by well-known pharmaceutical companies for improving the quality of chemical entities. Additionally, these recognition methods employ identification of the problematic fragments in a molecule. PAINS (for pan assay interference compounds) are molecules containing substructures showing potent response in assays irrespective of the protein target. Such fragments may yield false positive biological output. Our selected compounds revealed no alert for PAINS.

### 2.7. In Vitro Cytotoxicity Testing

The cytotoxic effects of compounds **5(a–m)** and **6(a–m)** against HepG2 cells were evaluated using cisplatin as a standard drug with an IC_50_ of 20 µg/mL. The evaluation of the acquired data indicated that compounds **5a**, **5m**, and **6j** are non-cytotoxic. On the other hand, the viability values of **5e**, **5f**, **6a**, **6b**, and **6m** refer them as cytotoxic. The rest of the compounds show proliferation in HepG2 cells hence are indicated as proliferative ones (Table 4). The graphical representation of the cytotoxicity results is given in Figures S1 and S2 (see Supplementary Materials).

## 3. Materials and Methods

### 3.1. General

The chemicals and solvents used were of analytical grade and obtained from commercial suppliers (Scharlau (Barcelona, Spain), Merck (Darmstadt, Germany), and Fluka (St. Louis, MO, USA), and were used without further purification. Thin layer chromatography was performed using aluminum plates coated with silica gel 60F_254_ (Merck) in an appropriate eluent. The spots were visualized using ultraviolet irradiation. Melting points were recorded on Gallenkamp melting point apparatus (Sigma-Aldrich Chemie GmbH, Taufkirchen, Germany) and were uncorrected. ^1^H NMR spectra were recorded in CDCl_3_ and DMSO-d_6_ solvents on a Bruker Avance NMR (300 MHz) spectrometer (Karlsruhe, Germany) while ^13^C NMR spectra were recorded at 75 MHz. Chemical shifts are reported as δ values in parts per million (ppm) relative to tetramethylsilane as internal standard. Coupling constant (*J*) is given in Hertz. FTIR spectra were recorded on an Agilent Technologies Cary 630 FTIR (Santa Clara, CA, USA). Elemental analysis was performed on a LECO 630-200-200 TRUSPEC CHNS micro analyzer (St. Joseph, MI, USA) and the values observed were within ±0.4% of the calculated results. Compounds **2** and **3** were synthesized following the literature procedures [60].

### 3.2. Preparation of Piperidinyl Quinoline-3-carbaldehydes ***(4a,b)***

Compounds **4a** and **4b** were prepared following literature procedure [61]. 

#### 3.2.1. Preparation of 2-Chloroquinoline-3-carbaldehydes **3(a,b)**

2-Chloroquinoline-3-carbaldehydes **3(a,b)** were prepared by using method reported by Meth-Cohn and coworkers. POCl_3_ (65.3 mL, 107.45 g, 0.70 mol) was added dropwise to DMF (19.3 mL, 18.26 g, 0.25 mol) with constant stirring while maintaining the temperature of the flask at 0 °C. To the resulting Vilsmeyer reagent, acetanilide **2** (0.10 mol) was added and the reaction mixture was heated at 70–80 °C. The progress of the reaction was monitored thin layer chromatography (TLC). The reaction mixture was then poured on crushed ice (500 g) cautiously and stirred vigorously at 0–10 °C. The precipitated 2-chloroquinoline-3-carbaldehyde **3** was filtered, washed with excess water, dried, and recrystallized from ethyl acetate.

#### 3.2.2. Preparation of Piperidinyl Quinoline-3-carbaldehydes (**4a,b**)

Piperidine (11 mmol) was added to a stirred solution of 2-chloro-6-methylquinoline-3-carbaldehyde **3a** or 2-chloro-8-methylquinoline-3-carbaldehyde **3b** (10 mmol) and catalytic amount of cetyltrimethylammonium bromide (CTAB) in PEG-400 (10 mL). The resulting reaction mixture was heated at 135 °C for 2.5 h. After cooling to room temperature, the reaction mixture was poured onto crushed ice and stirred overnight. The yellow precipitates were filtered, washed with water, dried and recrystallized from ethanol. 

#### 3.2.3. 6-Methyl-2-(piperidin-1-yl)quinoline-3-carbaldehyde (**4a**) Yield 98%

Yellow solid. Mp 90–92 °C (lit. 91–93 °C). FTIR (cm^−1^) 3030 (CH-aromatic), 2936 (CH), 2852 (CH-formyl), 1691 (C=O), 1572 (C=N, aromatic), 1053 (C-N); ^1^H NMR (CDCl_3_, 300 MHz) δ = 1.66–1.73 (m, 2H, piperidinyl-CH_2_), 1.77-1.84 (m, 4H, piperidinyl-CH_2_), 2.51 (s, 3H, CH_3_), 3.41-3.45 (m, 4H, piperidinyl-N-CH_2_), 7.52 (d, *J* = 1.8 Hz, 1H, ArH), 7.54–7.56 (m, 1H, ArH), 7.75 (d, *J* = 8.4 Hz, 1H, ArH), 8.42 (s, 1H, ArH), 10.18 (s, 1H, O=CH); ^13^C NMR (DMSO-*d*_6_, 75 MHz) δ = 21.2 (Q-CH_3_), 24.5 (piperidinyl-CH_2_), 25.8 (2C, piperidinyl-CH_2_), 52.2 (piperidinyl-N-CH_2_), 122.5 (C-3), 123.9 (C-10), 127.1 (C-8), 128.7 (C-5), 134.0 (C-6), 135.0 (C-7), 142.0 (C-4), 147.5 (C-9), 159.0 (C-2), 190.8 (C=O); Anal. Calcd. for C_16_H_18_N_2_O: C, 75.56; H, 7.13; N, 11.01%, Found: C, 75.79; H, 4.19; N, 11.12%.

#### 3.2.4. 8-Methyl-2-(piperidin-1-yl)quinoline-3-carbaldehyde (**4b**)

Yield 97%. Yellow solid. Mp 82–84 °C (lit. 83–85 °C). FTIR (cm^−1^): 3023 (CH-aromatic), 2928 (CH), 2851 (CH-formyl), 1687 (C=O), 1569 (C=N, aromatic), 1050 (C-N); ^1^H NMR (CDCl_3_, 300 MHz) δ = 1.69–1.76 (m, 2H, piperidinyl-CH_2_), 1.80–1.85 (m, 4H, piperidinyl-CH_2_), 2.70 (s, 3H, CH_3_), 3.48–3.51 (m, 4H, piperidinyl-N-CH_2_), 7.26 (t, *J* = 7.5 Hz, 1H, ArH), 7.56 (d, *J* = 6.9 Hz, 1H, ArH), 7.64 (d, *J* = 8.1 Hz, 1H, ArH), 8.47 (s, 1H, ArH), 10.18 (s, 1H, O=CH); ^13^C NMR (CDCl_3_, 75 MHz) δ = 17.7 (Q-CH_3_), 24.6 (piperidinyl-CH_2_), 25.9 (2C, piperidinyl-CH_2_), 52.5 (2C, piperidinyl-N-CH_2_), 121.7 (C-3), 123.6 (C-6), 124.0 (C-10), 127.1 (C-5), 132.3 (C-7), 135.6 (C-8), 141.6 (C-4), 148.2 (C-9), 158.9 (C-2), 190.8 (C=O); Anal. Calcd. for C_16_H_18_N_2_O: C, 75.56; H, 7.13; N, 11.01%, Found: C, 75.83; H, 4.32; N, 11.19%.

### 3.3. General Procedure for the Preparation of (Piperidin-1-yl)quinolin-3-yl)methylene)hydrazinecarbothioamides (***5,6***)

#### 3.3.1. Method A: Conventional Synthesis

To a stirred solution of **4a** or **4b** (254 mg, 1 mmol) in absolute ethanol (20 mL) was added N-substituted thiosemicarbazide (1 mmol) and catalytic amount of glacial acetic acid. The reaction mixture was heated to reflux for 0.5–2 h. The precipitated product was filtered, washed with ethanol, and dried to afford **5(a–m)** and **6(a–m)**.

#### 3.3.2. Method B: Microwave-Assisted Synthesis

The equimolar quantity of **4a** or **4b** (1 mmol) and *N*-substituted thiosemicarbazide (1 mmol) with catalytic amount of glacial acetic acid in absolute ethanol (20 mL) was exposed to microwave irradiation for 3–5 min. The precipitated solid was filtered, washed with hot ethanol and dried to afford **5(a–m)** and **6(a–m)**. 

Yields of **5(a–m)** and **6(a–m)** reported herein were observed by using method B.

2-((6-Methyl-2-(piperidin-1-yl)quinolin-3-yl)methylene)hydrazinecarbothioamide (**5a**) Yield 91%. Yellow fluffy solid. Mp 220–222 °C. FTIR (cm^−1^): 3476 (NH), 3329 (NH), 2927 (CH), 2803 (CH-imine), 1609 (C=N), 1119 (C=S); ^1^H NMR (DMSO-*d*_6_, 300 MHz) δ = 1.62–1.76 (m, 6H, piperidinyl-CH_2_), 2.46 (s, 3H, CH_3_), 3.23–3.26 (m, 4H, piperidinyl-N-CH_2_), 7.53 (d, *J* = 8.7 Hz, 1H, ArH), 7.60 (s, 1H, ArH), 7.74 (d, *J* = 8.1 Hz, 1H, ArH), 8.14 (br s, 1H, NH_2_), 8.20 (s, 1H, ArH), 8.37 (br s, 1H, NH_2_), 8.92 (s, 1H, N=CH), 11.79 (s, 1H, =N-NH); ^13^C NMR (DMSO-*d*_6_, 75 MHz) δ = 21.4 (Q-CH_3_), 24.3 (piperidinyl-CH_2_), 25.8 (2C, piperidinyl-CH_2_), 52.4 (2C, piperidinyl-N-CH_2_), 122.0 (C-3), 124.9 (C-10), 127.5 (2C, C-5, C-8), 133.1 (C-6), 134.6 (C-7), 139.0 (C-9), 145.7 (N=CH), 159.4 (C-2), 178.5 (C=S); Anal. Calcd. for C_17_H_21_N_5_S: C, 62.36; H, 6.46; N, 21.39; S, 9.79%. Found: C, 62.52; H, 6.71; N, 21.63; S, 9.91%.

2-((6-Methyl-2-(piperidin-1-yl)quinolin-3-yl)methylene)-*N*-phenylhydrazinecarbothioamide (**5b**) Yield 92%. Yellow fluffy solid. Mp 210–212 °C. FTIR (cm^−1^): 3281 (NH), 3128 (NH), 3061 (CH-aromatic), 2913 (CH), 2809 (CH-imine), 1596 (C=N), 1125 (C=S); ^1^H NMR (CDCl_3_, 300 MHz) δ =1.63–1.69 (m, 2H, piperidinyl-CH_2_), 1.75–1.82 (m, 4H, piperidinyl-CH_2_), 2.51 (s, 3H, CH_3_), 3.27–3.30 (m, 4H, piperidinyl-N-CH_2_), 7.42–7.55 (m, 4H, ArH), 7.29 (t, *J* = 7.2 Hz, 1H, ArH), 7.73–7.79 (m, 3H, ArH), 8.15 (s, 1H, ArH), 8.41 (s, 1H, N=CH), 9.32 (s, 1H, NH), 10.14 (s, 1H, =N-NH); ^13^C NMR (DMSO-*d*_6_, 75 MHz) δ = 21.4 (Q-CH_3_), 24.5 (piperidinyl-CH_2_), 25.9 (2C, piperidinyl-CH_2_), 52.4 (2C, piperidinyl-N-CH_2_), 121.6 (C-3), 125.2 (C-10), 125.9 (C-4′), 126.5 (2C, C-2′, C-6′), 127.3 (C-8), 127.5 (C-5), 128.6 (2C, C-3′, C-5′), 132.7 (C-6), 134.2 (C-7), 135.4 (C-4), 139.5 (C-1′), 140.4 (C-9), 145.7 (N=CH), 160.3 (C-2), 176.4 (C=S); Anal. Calcd. for C_23_H_25_N_5_S: C, 68.46; H, 6.24; N, 17.35; S, 7.95%. Found: C, 68.55; H, 6.39; N, 17.47; S, 8.01%.

*N*-(2,4-Dimethylphenyl)-2-((6-methyl-2-(piperidin-1-yl)quinolin-3-yl)methylene)hydrazine carbothioamide (**5c**) Yield 92%. Light yellow solid. Mp 218–220 °C. FTIR (cm^−1^): 3329 (NH), 3118 (NH), 2917 (CH), 2831 (CH-imine), 1617 (C=N), 1124 (C=S); ^1^H NMR (CDCl_3_, 300 MHz) δ = 1.66–1.70 (m, 2H, piperidinyl-CH_2_), 1.75–1.82 (m, 4H, piperidinyl-CH_2_), 2.38 (s, 6H, CH_3_), 2.50 (s, 3H, CH_3_), 3.27–3.30 (m, 4H, piperidinyl-N-CH_2_), 7.10–7.13 (m, 2H, ArH), 7.4–7.54 (m, 3H, ArH), 7.78 (d, *J* = 8.4 Hz, 1H, ArH), 8.12 (s, 1H, ArH), 8.39 (s, 1H, N=CH), 9.00 (s, 1H, NH), 10.07 (s, 1H, =N-NH); ^13^C NMR (DMSO-*d*_6_, 75 MHz) δ = 18.3 (Ar-CH_3_), 21.1 (Ar-CH_3_), 21.4 (Q-CH_3_), 24.5 (piperidinyl-CH_2_), 25.9 (2C, piperidinyl-CH_2_), 52.4 (2C, piperidinyl-N-CH_2_), 121.8 (C-3), 125.3 (C-10), 127.0 (C-8), 127.3 (C-6′), 127.5 (C-5), 129.1 (C-5′), 131.1 (C-3′), 132.6 (C-6), 134.1 (C-7), 135.2 (C-4), 135.8 (C-1′), 135.9 (C-2′), 136.4 (C-4′), 139.7 (C-9), 145.7 (N=CH), 160.3 (C-2), 177.4 (C=S); Anal. Calcd. for C_25_H_29_N_5_S: C, 69.57; H, 6.77; N, 16.23; S, 7.43%. Found: C, 69.85; H, 6.92; N, 16.51; S, 7.73%.

*N*-(2,6-Dimethylphenyl)-2-((6-methyl-2-(piperidin-1-yl)quinolin-3-yl)methylene)hydrazine carbothioamide (**5d**) Yield 95%. Yellow solid. Mp 244–246 °C. FTIR (cm^−1^): 3332 (NH), 3128 (NH), 2937 (CH), 1604 (C=N), 1125 (C=S); ^1^H NMR (CDCl_3_, 300 MHz) δ = 1.67–1.71 (m, 2H, piperidinyl-CH_2_), 1.77–1.81 (m, 4H, piperidinyl-CH_2_), 2.39 (s, 6H, CH_3_), 2.50 (s, 3H, CH_3_), 3.28–3.31 (m, 4H, piperidinyl-N-CH_2_), 7.07–7.27 (m, 3H, ArH), 7.47–7.55 (m, 2H, ArH), 7.77–7.80 (m, 1H, ArH), 8.10 (s, 1H, ArH), 8.40 (s, 1H, ArH), 8.76 (s, 1H, NH), 9.86 (s, 1H, =N-NH); ^13^C NMR (CDCl_3_, 75 MHz) δ = 18.5 (Ar-CH_3_), 18.8 (Ar-CH_3_), 21.4 (Q-CH_3_), 24.5 (piperidinyl-CH_2_), 26.1 (2C, piperidinyl-CH_2_), 52.5 (2C, piperidinyl-N-CH_2_), 120.5 (C-3), 124.8 (C-4′), 125.1 (C-10), 127.0 (C-8), 128.3 (C-5), 12.5 (2C, C-3′, C-5′), 132.8 (C-6), 134.6 (C-7), 135.2 (C-4), 136.7 (2C, C-1′), 137.8 (2C, C-2′, C-6′), 140.3 (C-9), 146.2 (N=CH), 160.3 (C-2), 177.4 (C=S); Anal. Calcd. for C_25_H_29_N_5_S: C, 69.57; H, 6.77; N, 16.23; S, 7.43%. Found: C, 69.29; H, 6.58; N, 16.07; S, 7.27%.

*N*-(2-Chlorophenyl)-2-((6-methyl-2-(piperidin-1-yl)quinolin-3-yl)methylene)hydrazine carbothioamide (**5e**) Yield 91%. Yellow solid. Mp 208–210 °C. FTIR (cm^−1^): 3246 (NH), 3109 (NH), 2915 (CH), 2810 (CH-imine), 1593 (C=N), 1124 (C=S); ^1^H NMR (CDCl_3_, 300 MHz) δ = 1.63–1.68 (m, 2H, piperidinyl-CH_2_), 1.76–1.83 (m, 4H, piperidinyl-CH_2_), 2.51 (s, 3H, CH_3_), 3.27–3.30 (m, 4H, piperidinyl-N-CH_2_), 7.20 (td, *J* = 7.8, 1.2 Hz, 1H, ArH), 7.38 (td, *J* = 8.1, 1.2 Hz, 1H, ArH), 7.47–7.52 (m, 3H, ArH), 7.78 (d, *J* = 8.4 Hz, 1H, ArH), 8.18 (s, 1H, ArH), 8.46 (s, 1H, N=CH), 8.74 (dd, J = 8.4, 0.9 Hz, 1H), 9.91 (s, 1H, NH), 10.21 (s, 1H, =N-NH); ^13^C NMR (DMSO-*d*_6_, 75 MHz) δ = 21.4 (Q-CH_3_), 24.5 (piperidinyl-CH_2_), 25.9 (2C, piperidinyl-CH_2_), 52.5 (2C, piperidinyl-N-CH_2_), 121.6 (C-3), 125.2 (C-10), 127.3 (C-8), 127.5 (C-5), 127.7 (C-5′), 128.5 (C-3′), 129.8 (C-2′), 130.8 (C-4′), 131.6 (C-6′), 132.7 (C-6), 134.2 (C-7), 135.2 (C-4), 137.0 (C-1′), 140.4 (C-9), 145.8 (N=CH), 160.3 (C-2), 177.2 (C=S); Anal. Calcd. for C_23_H_24_ClN_5_S: C, 63.07; H, 5.52; N, 15.99; S, 7.32%. Found: C, 63.19; H, 5.61; N, 16.11; S, 7.52%.

*N*-(3-Chlorophenyl)-2-((6-methyl-2-(piperidin-1-yl)quinolin-3-yl)methylene)hydrazine carbothioamide (**5f**) Yield 97%. Yellow solid. Mp 216–218 °C. FTIR (cm^−1^): 3325 (NH), 3127 (NH), 2931 (CH), 2813 (CH-imine), 1600 (C=N), 1125 (C=S); ^1^H NMR (DMSO-*d*_6_, 300 MHz) δ = 1.61–1.76 (m, 6H, piperidinyl-CH_2_), 2.44 (s, 3H, CH_3_), 3.17–3.20 (m, 4H, piperidinyl-N-CH_2_), 7.29 (dd, *J* = 8.1, 0.9 Hz, 1H, ArH), 7.40–7.50 (m, 2H, ArH), 7.62–7.67 (m, 3H, ArH), 7.81 (t, *J* = 2.1 Hz, 1H, ArH), 8.36 (s, 1H, N=CH), 8.92 (s, 1H), 10.26 (s, 1H, NH), 12.78 (s, 1H, =N-NH); ^13^C NMR (DMSO-d_6_, 75 MHz) δ = 21.4 (Q-CH_3_), 24.4 (piperidinyl-CH_2_), 25.9 (2C, piperidinyl-CH_2_), 52.4 (2C, piperidinyl-N-CH_2_), 121.5 (C-3), 124.8 (C-6′), 125.2 (C-2′), 125.6 (C-4′), 125.7 (C-10), 127.4 (C-8), 127.5 (C-5), 130.1 (C-5′), 132.7 (C-3′), 132.8 (C-6), 134.3 (C-7), 135.5 (C-4), 140.9 (C-9), 141.0 (C-1′), 145.8 (N=CH), 160.3 (C-2), 176.2 (C=S); Anal. Calcd. for C_23_H_24_ClN_5_S: C, 63.07; H, 5.52; N, 15.99; S, 7.32%. Found: C, 63.39; H, 5.81; N, 16.15; S, 7.60%.

*N*-(4-Chlorophenyl)-2-((6-methyl-2-(piperidin-1-yl)quinolin-3-yl)methylene)hydrazine carbothioamide (**5g**) Yield 97%. Yellow solid. Mp 217–219 °C. FTIR (cm^−1^): 3295 (NH), 3137 (NH), 2936 (CH), 2826 (CH-imine), 1608 (C=O), 1124 (C=S); ^1^H NMR (DMSO-d_6_, 300 MHz) δ = 1.61–1.76 (m, 6H, piperidinyl-CH_2_), 2.44 (s, 3H, CH_3_), 3.17–3.20 (m, 4H, piperidinyl-N-CH_2_), 7.44–7.50 (m, 3H, ArH), 7.61–7.68 (m, 4H, ArH), 8.36 (s, 1H, ArH), 8.92 (s, 1H, N=CH), 10.23 (s, 1H, NH), 12.22 (s, 1H, =N-NH); ^13^C NMR (CDCl_3_, 75 MHz) δ = 21.4 (Q-CH_3_), 24.5 (piperidinyl-CH_2_), 26.0 (2C, piperidinyl-CH_2_), 52.5 (2C, piperidinyl-N-CH_2_), 121.6 (C-3), 125.2 (C-10), 127.3 (C-8), 127.5 (C-5), 127.7 (C-6′), 128.5 (C-2′), 129.8 (C-4′), 130.8 (C-6), 135.1 (C-7), 136.2 (C-4), 137.0 (C-1′), 140.4 (C-9), 145.5 (N=CH), 160.3 (C-2), 177.0 (C=S); Anal. Calcd. for C_23_H_24_ClN_5_S: C, 63.07; H, 5.52; N, 15.99; S, 7.32%. Found: C, 63.23; H, 5.75; N, 16.08; S, 7.45%.

*N*-(2-Fluorophenyl)-2-((6-methyl-2-(piperidin-1-yl)quinolin-3-yl)methylene)hydrazine carbothioamide (**5h**) Yield 90%. Yellow solid. Mp 207–209 °C. FTIR (cm^−1^): 3282 (NH), 3118 (NH), 2928 (CH), 2826 (CH-imine), 1620 (C=N), 1125 (C=S); ^1^H NMR (CDCl_3_, 300 MHz) δ = 1.68–1.83 (m, 6H, piperidinyl-CH_2_), 2.51 (s, 3H, CH_3_), 3.35–3.38 (m, 4H, piperidinyl-N-CH_2_), 7.15–7.26 (m, 3H, ArH), 7.53–7.56 (m, 3H, ArH), 8.24 (s, 1H, ArH), 8.42–8.49 (m, 2H, N=CH, ArH), 9.43 (s, 1H, NH), 10.15 (s, 1H, =N-NH); ^13^C NMR (CDCl_3_, 75 MHz) δ = 21.4 (Q-CH_3_), 25.1 (piperidinyl-CH_2_), 26.0 (2C, piperidinyl-CH_2_), 52.9 (2C, piperidinyl-N-CH_2_), 115.4 (C-3′, d, *J* = 19.5 Hz), 120.6 (C-3), 124.0 (C-10), 124.1 (C-6′), 125.5 (C-8), 126.0 (C-5), 126.2 (C-4′), 126.7 (C-5′), 126.9 (C-1′), 127.2 (C-8), 127.6 (C-5), 129.6 (C-4), 133.7 (C-6), 153.4 (N=CH), 156.7 (C-2), 175.5 (C-2′, C=S); Anal. Calcd. for C_23_H_24_FN_5_S: C, 65.53; H, 5.74; N, 16.61; S, 7.61%. Found: C, 65.64; H, 5.90; N, 16.81; S, 7.79%.

*N*-(3-Fluorophenyl)-2-((6-methyl-2-(piperidin-1-yl)quinolin-3-yl)methylene)hydrazine carbothioamide (**5i**) Yield 96%. Yellow solid. Mp 206–208 °C. FTIR (cm^−1^): 3336 (NH), 3133 (NH), 2934 (CH), 2813 (CH-imine), 1602 (C=N), 1163 (C=S); ^1^H NMR (DMSO-*d*_6_, 300 MHz) δ = 1.61–1.76 (m, 6H, piperidinyl-CH_2_), 2.44 (s, 3H, CH_3_), 3.17–3.20 (m, 4H, piperidinyl-N-CH_2_), 7.07 (td, *J* = 8.4, 1.8 Hz, 1H, ArH), 7.39–7.52 (m, 3H, ArH), 7.62–7.67 (m, 3H, ArH), 8.36 (s, 1H, ArH), 8.92 (s, 1H, N=CH), 10.26 (s, 1H, NH), 12.27 (s, 1H, =N-NH); ^13^C NMR (DMSO-*d*_6_, 75 MHz) δ = 21.4 (Q-CH_3_), 24.5 (piperidinyl-CH_2_), 25.9 (2C, piperidinyl-CH_2_), 52.4 (2C, piperidinyl-N-CH_2_), 112.4 (C-4′, d, *J* = 20.3 Hz), 112.8 (C-2′, d, *J* = 24.8 Hz), 121.5 (C-3), 121.9 (C-6′, d, *J* = 2.3 Hz), 125.2 (C-10), 127.4 (C-8), 127.5 (C-5), 130.1 (C-5′, d, *J* = 9.0 Hz), 132.8 (C-6), 134.3 (C-7), 135.5 (C-4), 140.9 (C-9), 141.2 (C-1′, d, *J* = 10.5 Hz), 145.8 (N=CH), 160.3 (C-2), 162.0 (C-3′, d, *J* = 240.0 Hz), 176.1 (C=S); Anal. Calcd. for C_23_H_24_FN_5_S: C, 65.53; H, 5.74; N, 16.61; S, 7.61%. Found: C, 65.80; H, 5.94; N, 16.79; S, 7.83%.

*N*-(4-Fluorophenyl)-2-((6-methyl-2-(piperidin-1-yl)quinolin-3-yl)methylene)hydrazine carbothioamide (**5j**) Yield 90%. Yellow solid. Mp 213–215 °C. FTIR (cm^−1^): 3332 (NH), 2956 (CH), 2830 (CH-imine), 1603 (C=N), 1124 (C=S); ^1^H NMR (DMSO-*d*_6_, 300 MHz) δ = 1.61–1.76 (m, 6H, piperidinyl-CH_2_), 2.44 (s, 3H, CH_3_), 3.17–3.20 (m, 4H, piperidinyl-N-CH_2_), 7.24 (td, *J* = 7.8, 2.1 Hz, 2H, ArH), 7.48 (dd, *J* = 8.4, 1.8 Hz, 1H, ArH), 7.56–7.61 (m, 3H, ArH), 7.66 (d, *J* = 8.4, 1H, ArH), 8.35 (s, 1H, ArH), 8.93 (s, 1H, N=CH), 10.19 (s, 1H, NH), 12.17 (s, 1H, =N-NH); ^13^C NMR (DMSO-*d*_6_, 75 MHz) δ = 21.4 (Q-CH_3_), 24.5 (piperidinyl-CH_2_), 25.9 (2C, piperidinyl-CH_2_), 52.4 (2C, piperidinyl-N-CH_2_), 115.3 (2C, C-3′, C-5′, d, *J* = 22.5 Hz), 121.6 (C-3), 125.2 (C-10), 127.3 (C-8), 127.5 (C-5), 128.7 (2C, C-2′, C-6′, d, *J* = 8.3 Hz), 132.7 (C-6), 134.2 (C-7), 135.4 (C-4), 135.9 (C-1′, d, *J* = 2.3 Hz), 140.5 (C-9), 145.7 (N=CH), 160.2 (C-4′, d, *J* = 240.8 Hz), 160.3 (C-2), 176.8 (C=S); Anal. Calcd. for C_23_H_24_FN_5_S: C, 65.53; H, 5.74; N, 16.61; S, 7.61%. Found: C, 65.35; H, 5.45; N, 16.40; S, 7.55%.

*N*-(4-Ethylphenyl)-2-((6-methyl-2-(piperidin-1-yl)quinolin-3-yl)methylene)hydrazine carbothioamide (**5k**) Yield 97%. Yellow solid. Mp 218–220 °C. FTIR (cm^−1^): 3274 (NH), 3112 (NH), 2964 (CH), 2914 (CH), 2820 (CH-imine), 1604 (C=N), 1125 (C=S); ^1^H NMR (DMSO-*d*_6_, 300 MHz) δ = 1.21 (t, *J* = 7.5 Hz, 3H, CH_3_), 1.61–1.76 (m, 6H, piperidinyl-CH_2_), 2.44 (s, 3H, CH_3_), 2.63 (q, *J* = 7.5 Hz, 2H, CH_2_), 3.17–3.20 (m, 4H, piperidinyl-N-CH_2_), 7.23 (d, *J* = 8.4 Hz, 2H, ArH), 7.47–7.50 (m, 3H, ArH), 7.59–7.67 (m, 2H, ArH), 8.34 (s, 1H, ArH), 8.94 (s, 1H, N=CH), 10.12 (s, 1H, NH), 12.11 (s, 1H, =N-NH); ^13^C NMR (DMSO-*d*_6_, 75 MHz) δ = 16.2 (Ar-CH_2_-CH_3_), 21.4 (Q-CH_3_), 24.5 (piperidinyl-CH_2_), 25.9 (2C, piperidinyl-CH_2_), 28.2 (Ar-CH_2_-CH_3_), 52.4 (2C, piperidinyl-N-CH_2_), 121.6 (C-3), 125.2 (C-10), 126.5 (2C, C-2′, C-6′), 127.3 (C-8), 127.5 (C-5), 127.9 (2C, C-3′, C-5′), 132.7 (C-4′), 134.2 (C-6), 135.3 (C-7), 137.1 (C-4), 140.2 (C-1′), 141.5 (C-9), 145.7 (N=CH), 160.2 (C-2), 176.4 (C=S); Anal. Calcd. for C_25_H_29_N_5_S: C, 69.57; H, 6.77; N, 16.23; S, 7.43%. Found: C, 69.61; H, 6.85; N, 16.33; S, 7.50%.

*N*-Benzyl-2-((6-methyl-2-(piperidin-1-yl)quinolin-3-yl)methylene)hydrazinecarbothioamide (**5l**) Yield 95%. Yellow solid. Mp 208–210 °C. FTIR (cm^−1^): 3382 (NH), 3116 (NH), 2971 (CH), 2932 (CH), 2841 (CH-imine), 1602 (C=N), 1107 (C=S); ^1^H NMR (DMSO-*d*_6_, 300 MHz) δ = 1.60–1.74 (m, 6H, piperidinyl-CH_2_), 2.43 (s, 3H, CH_3_), 3.15–3.18 (m, 4H, piperidinyl-N-CH_2_), 4.90 (d, *J* = 6.3 Hz, 2H, N-CH_2_), 7.25 (tt, *J* = 6.9, 1.5 Hz, 1H, ArH), 7.32–7.41 (m, 4H, ArH), 7.46 (dd, *J* = 8.4, 1.8 Hz, 1H, ArH), 7.57 (s, 1H, ArH), 7.64 (d, *J* = 8.7 Hz, 1H, ArH), 8.29 (s, 1H, ArH), 8.77 (s, 1H, N=CH), 9.17 (t, *J* = 6.3 Hz, 1H, NH), 11.92 (s, 1H, =N-NH); ^13^C NMR (DMSO-*d*_6_, 75 MHz) δ = 21.4 (Q-CH_3_), 24.5 (piperidinyl-CH_2_), 25.9 (2C, piperidinyl-CH_2_), 47.1 (Ar-CH_2_-NH), 52.4 (2C, piperidinyl-N-CH_2_), 121.8 (C-3), 125.1 (C-10), 127.2 (C-8), 127.5 (C-5), 127.7 (3C, C-2′, C-4′, C-6′), 128.7 (2C, C-3′, C-5′), 132.6 (C-6), 134.2 (C-7), 134.8 (C-4), 139.8 (C-1′), 139.9 (C-9), 145.7 (N=CH), 160.2 (C-2), 178.0 (C=S); Anal. Calcd. for C_24_H_27_N_5_S: C, 69.03; H, 6.52; N, 16.77; S, 7.68%. Found: C, 69.15; H, 6.66; N, 16.89; S, 7.75%.

2-((6-Methyl-2-(piperidin-1-yl)quinolin-3-yl)methylene)-*N*-(2-morpholinoethyl)hydrazine carbothioamide (**5m**) Yield 89%. Yellow solid. Mp 212–214 °C. FTIR (cm^−1^): 3210 (NH), 3124 (NH), 2930 (CH), 2799 (CH-imine), 1603 (C=N), 1111 (C=S); ^1^H NMR (CDCl_3_, 300 MHz) δ = 1.66–1.69 (m, 2H, piperidinyl-CH_2_), 1.74–1.81 (m, 4H, piperidinyl-CH_2_), 2.49 (s, 3H, CH_3_), 2.78–2.90 (m, 6H, NCH_2_ linked to morpholine ring), 3.23–3.26 (m, 4H, piperidinyl-N-CH_2_), 3.93–3.97 (m, 6H, NCH_2_, OCH_2_ of morpholine ring), 7.47 (d, J = 8.4 Hz, 1H, ArH), 7.52 (s, 1H, ArH), 7.75 (d, *J* = 8.4 Hz, 1H, ArH), 8.04 (s, 1H, ArH), 8.43 (s, 1H, N=CH), 8.52 (t, *J* = 6.0 Hz, 1H, NH), 9.71 (s, 1H, =N-NH); ^13^C NMR (CDCl_3_, 75 MHz) δ = 21.5 (Q-CH_3_), 24.5 (piperidinyl-CH_2_), 26.1 (2C, piperidinyl-CH_2_), 40.1 (NH-CH_2_-CH_2_-N(morpholine)), 52.4 (2C, piperidinyl-N-CH_2_), 53.2 (NH-CH_2_-CH_2_-N(morpholine)), 56.7 (-N-CH_2_-CH_2_-O(morpholine)), 66.5 (N-CH_2_-CH_2_-O(morpholine)),120.7 (C-3), 125.0 (C-10), 127.0 (C-8), 127.4 (C-5), 132.7 (C-6), 134.5 (C-7), 135.0 (C-4), 140.3 (C-9), 146.2 (N=CH), 160.3 (C-2), 177.1 (C=S); Anal. Calcd. for C_23_H_32_N_6_OS: C, 62.70; H, 7.32; N, 19.07; S, 7.28%. Found: C, 62.59; H, 7.21; N, 19.00; S, 7.19%.

2-((8-Methyl-2-(piperidin-1-yl)quinolin-3-yl)methylene)hydrazinecarbothioamide (**6a**) Yield 90%. Yellow solid. Mp 222–224 °C. FTIR (cm^−1^): 3471 (NH), 3330 (NH), 2929 (CH), 2803 (CH-imine), 1608 (C=N), 1119 (C=S); ^1^H NMR (DMSO-*d*_6_, 300 MHz) δ = 1.62–1.76 (m, 6H, piperidinyl-CH_2_), 2.61 (s, 3H, CH_3_), 3.22–3.24 (m, 4H, piperidinyl-N-CH_2_), 7.30 (t, *J* = 7.5 Hz, 1H, ArH), 7.51 (d, *J* = 6.9 Hz, 1H, ArH), 7.65 (d, *J* = 7.8 Hz, 1H, ArH), 8.13 (br s, 1H, NH), 8.24 (s, 1H, ArH), 8.33 (br s, 1H, NH), 8.89 (s, 1H, N=CH), 11.74 (s, 1H, =N-NH); ^13^C NMR (DMSO-*d*_6_, 75 MHz) δ = 17.8 (Q-CH_3_), 24.5 (piperidinyl-CH_2_), 25.8 (2C, piperidinyl-CH_2_), 52.2 (2C, piperidinyl-N-CH_2_), 121.1 (C-3), 124.6 (C-6), 124.9 (C-10), 126.3 (C-5), 130.5 (C-7), 135.0 (C-8), 135.9 (C-4), 139.7 (C-9), 145.8 (N=CH), 159.5 (C-2), 178.4 (C=S); Anal. Calcd. for C_17_H_21_N_5_S: C, 62.36; H, 6.46; N, 21.39; S, 9.79%. Found: C, 62.51; H, 6.65; N, 21.51; S, 9.91%.

2-((8-Methyl-2-(piperidin-1-yl)quinolin-3-yl)methylene)-*N*-phenylhydrazinecarbothioamide (**6b**) Yield 93%. Yellow solid. Mp 208–210 °C. FTIR (cm^−1^): 3283 (NH), 3128 (NH), 3060 (CH-aromatic), 2910 (CH), 2807 (CH-imine), 1595 (C=N), 1125 (C=S); ^1^H NMR (DMSO-*d*_6_, 300 MHz) δ = 1.64–1.79 (m, 6H, piperidinyl-CH_2_), 2.63 (s, 3H, CH_3_), 3.25–3.28 (m, 4H, piperidinyl-N-CH_2_), 7.24 (tt, *J* = 7.5, 1.2 Hz, 1H, ArH), 7.31 (t, *J* = 7.5 Hz, 1H, ArH), 7.38–7.43 (m, 2H, ArH), 7.52 (d, *J* = 6.9 Hz, 1H, ArH), 7.60 (d, *J* = 7.5 Hz, 2H, ArH), 7.68 (d, *J* = 7.8 Hz, 1H, ArH), 8.36 (s, 1H, ArH), 8.99 (s, 1H, N=CH), 10.22 (s, 1H, NH), 12.15 (s, 1H, =N-NH); ^13^C NMR (DMSO-*d*_6_, 75 MHz) δ = 17.8 (Q-CH_3_), 24.5 (piperidinyl-CH_2_), 25.9 (2C, piperidinyl-CH_2_), 52.2 (2C, piperidinyl-N-CH_2_), 121.0 (C-3), 124.7 (C-6), 124.9 (C-10), 126.0 (C-4′), 126.4 (C-5), 126.6 (2C, C-2′, C-6′), 128.6 (2C, C-3′, C-5′), 130.6 (C-7), 135.1 (C-8), 136.3 (C-4), 139.5 (C-9), 140.4 (C-1′), 145.9 (N=CH), 159.5 (C-2), 176.4 (C=S); Anal. Calcd. for C_23_H_25_N_5_S: C, 68.46; H, 6.24; N, 17.35; S, 7.95%. Found: C, 68.70; H, 6.49; N, 17.53; S, 8.07%.

*N*-(2,4-Dimethylphenyl)-2-((8-methyl-2-(piperidin-1-yl)quinolin-3-yl)methylene)hydrazine carbothioamide (**6c**) Yield 91%. Yellow solid. Mp 218–220 °C. FTIR (cm^−1^): 3329 (NH), 3120 (NH), 2927 (CH), 2831 (CH-imine), 1616 (C=N), 1124 (C=S); ^1^H NMR (DMSO-d_6_, 300 MHz) δ = 1.64–1.79 (m, 6H, piperidinyl-CH_2_), 2.23 (s, 3H, CH_3_), 2.32 (s, 3H, CH_3_), 2.62 (s, 3H, CH_3_), 3.25–3.28 (m, 4H, piperidinyl-N-CH_2_), 7.05 (dd, J = 8.1, 1.5 Hz, 1H, ArH), 7.11–7.17 (m, 2H, ArH), 7.30 (t, *J* = 7.5 Hz, 1H, ArH), 7.51 (d, *J* = 6.9 Hz, 1H, ArH), 7.63 (d, *J* = 7.8 Hz, 1H, ArH), 8.34 (s, 1H, ArH), 9.02 (s, 1H, N=CH), 10.00 (s, 1H, NH), 12.07 (s, 1H, =N-NH); ^13^C NMR (DMSO-*d*_6_, 75 MHz) δ = 17.8 (Q-CH_3_), 18.3 (Ar-CH_3_), 21.1 (Ar-CH_3_), 24.6 (piperidinyl-CH_2_), 25.9 (2C, piperidinyl-CH_2_), 52.3 (2C, piperidinyl-N-CH_2_), 121.1 (C-3), 124.6 (C-6), 125.0 (C-10), 126.3 (C-5), 127.0 (C-6′), 129.2 (C-5′), 130.5 (C-7), 131.1 (C-3′), 135.1 (C-8), 135.9 (C-4), 136.0 (C-2′), 136.1 (C-4′), 136.4 (C-1′), 139.7 (C-9), 145.9 (N=CH), 159.6 (C-2), 177.4 (C=S); Anal. Calcd. for C_25_H_29_N_5_S: C, 69.57; H, 6.77; N, 16.23; S, 7.43%. Found: C, 69.30; H, 6.59; N, 16.09; S, 7.20%.

*N*-(2,6-Dimethylphenyl)-2-((8-methyl-2-(piperidin-1-yl)quinolin-3-yl)methylene)hydrazine carbothioamide (**6d**) Yield 98%. Lemon yellow solid. Mp 226–228 °C. FTIR (cm^−1^): 3333 (NH), 3128 (NH), 2931 (CH), 1606 (C=N), 1124 (C=S); ^1^H NMR (DMSO-*d*_6_, 300 MHz) δ = 1.64–1.79 (m, 6H, piperidinyl-CH_2_), 2.62 (s, 3H, CH_3_), 2.23 (s, 6H, CH_3_), 3.25–3.27 (m, 4H, piperidinyl-N-CH_2_), 7.07–7.15 (m, 3H, ArH), 7.29 (t, *J* = 7.6 Hz, 1H, ArH), 7.50 (d, *J* = 6.9 Hz, 1H, ArH), 7.61 (d, *J* = 7.8 Hz, 1H, ArH), 8.34 (s, 1H, ArH), 9.07 (s, 1H, N=CH), 9.96 (s, 1H, NH), 12.07 (s, 1H, =N-NH); ^13^C NMR (DMSO-*d*_6_, 75 MHz) δ = 17.8 (Q-CH_3_), 18.6 (2C, Ar-CH_3_), 24.5 (piperidinyl-CH_2_), 25.9 (2C, piperidinyl-CH_2_), 52.3 (2C, piperidinyl-N-CH_2_), 121.2 (C-3), 124.6 (C-6), 125.0 (C-10), 126.2 (C-5), 127.5 (C-4′), 128.1 (2C, C-3′, C-5′), 135.1 (C-8), 136.0 (C-4), 136.5 (C-1′), 137.0 (2C, C-2′, C-6′), 137.6 (C-7), 139.5 (C-9), 145.8 (N=CH), 159.6 (C-2), 177.1 (C=S); Anal. Calcd. for C_25_H_29_N_5_S: C, 69.57; H, 6.77; N, 16.23; S, 7.43%. Found: C, 69.69; H, 6.85; N, 16.31; S, 7.50%.

*N*-(2-Chlorophenyl)-2-((8-methyl-2-(piperidin-1-yl)quinolin-3-yl)methylene)hydrazine carbothioamide (**6e**) Yield 93%. Lemon yellow solid. Mp 202–204 °C. FTIR (cm^−1^): 3241 (NH), 3108 (NH), 2915 (CH), 2810 (CH-imine), 1593 (C=N), 1125 (C=S); ^1^H NMR (DMSO-*d*_6_, 300 MHz) δ = 1.63–1.78 (m, 6H, piperidinyl-CH_2_), 2.62 (s, 3H, CH_3_), 3.25–3.28 (m, 4H, piperidinyl-N-CH_2_), 7.30 (t, *J* = 7.5 Hz, 1H, ArH), 7.35–7.44 (m, 2H, ArH), 7.51 (d, *J* = 6.9 Hz, 1H, ArH), 7.58 (dd, *J* = 7.8, 1.5 Hz, 1H, ArH), 7.62–7.66 (m, 2H, ArH), 8.36 (s, 1H, ArH), 8.95 (s, 1H, N=CH), 10.18 (s, 1H, NH), 12.27 (s, 1H, =N-NH); ^13^C NMR (DMSO-*d*_6_, 75 MHz) δ = 17.8 (Q-CH_3_), 24.5 (piperidinyl-CH_2_), 25.9 (2C, piperidinyl-CH_2_), 52.3 (2C, piperidinyl-N-CH_2_), 120.9 (C-3), 124.7 (C-6), 124.9 (C-10), 126.3 (C-5), 127.7 (C-5′), 128.6 (C-2′), 129.9 (C-3′), 130.7 (C-7), 131.0 (C-4′), 131.8 (C-6′), 135.1 (C-8), 136.1 (C-4), 137.1 (C-1′), 140.4 (C-9), 145.9 (N=CH), 159.6 (C-2), 177.3 (C=S); Anal. Calcd. for C_23_H_24_ClN_5_S: C, 63.07; H, 5.52; N, 15.99; S, 7.32%. Found: C, 63.25; H, 5.77; N, 16.11; S, 7.53%.

*N*-(3-Chlorophenyl)-2-((8-methyl-2-(piperidin-1-yl)quinolin-3-yl)methylene)hydrazine carbothioamide (**6f**) Yield 93%. Lemon yellow solid. Mp 198–200 °C. FTIR (cm^−1^): 3321 (NH), 3128 (NH), 2933 (CH), 2813 (CH-imine), 1601 (C=N), 1125 (C=S); ^1^H NMR (DMSO-*d*_6_, 300 MHz) δ = 1.63–1.78 (m, 6H, piperidinyl-CH_2_), 2.62 (s, 3H, CH_3_), 3.24–3.28 (m, 4H, piperidinyl- N-CH_2_), 7.28–7.33 (m, 2H, ArH), 7.42 (t, *J* = 8.1 Hz, 1H, ArH), 7.52 (d, *J* = 6.9 Hz, 1H, ArH), 7.63 (d, *J* = 8.1 Hz, 1H, ArH), 7.69 (d, *J* = 7.8 Hz, 1H, ArH), 7.79 (t, *J* = 2.1 Hz, 1H, ArH), 8.36 (s, 1H, ArH), 8.97 (s, 1H, N=CH), 10.28 (s, 1H, NH), 12.26 (s, 1H, =N-NH); ^13^C NMR (DMSO-*d*_6_, 75 MHz) δ = 17.8 (Q-CH_3_), 24.5 (piperidinyl-CH_2_), 25.9 (2C, piperidinyl-CH_2_), 52.3 (2C, piperidinyl-N-CH_2_), 120.8 (C-3), 124.7 (C-6), 124.8 (C-10), 124.9 (C-6′), 125.6 (C-2′), 125.8 (C-4′), 126.4 (C-5), 130.1 (C-5′), 130.7 (C-7), 132.7 (C-3′), 135.1 (C-8), 136.4 (C-4), 140.9 (C-9), 141.0 (C-1′), 146.0 (N=CH), 159.5 (C-2), 176.2 (C=S); Anal. Calcd. for C_23_H_24_ClN_5_S: C, 63.07; H, 5.52; N, 15.99; S, 7.32%. Found: C, 62.99; H, 5.37; N, 15.88; S, 7.20%.

*N*-(4-Chlorophenyl)-2-((8-methyl-2-(piperidin-1-yl)quinolin-3-yl)methylene)hydrazine carbothioamide (**6g**) Yield 95%. Yellow solid. Mp 190–192 °C. FTIR (cm^−1^): 3291 (NH), 3137 (NH), 2937 (CH), 2826 (CH-imine), 1609 (C=O), 1124 (C=S); ^1^H NMR (DMSO-*d*_6_, 300 MHz) δ = 1.63–1.77 (m, 6H, piperidinyl-CH_2_), 2.62 (s, 3H, CH_3_), 3.25–3.27 (m, 4H, piperidinyl-N-CH_2_), 7.30 (t, *J* = 7.5 Hz, 1H, ArH), 7.43–7.47 (m, 2H, ArH), 7.51 (d, *J* = 6.9 Hz, 1H, ArH), 7.63–7.69 (m, 3H, ArH), 8.36 (s, 1H, ArH), 8.96 (s, 1H, N=CH), 10.28 (s, 1H, NH), 11.81 (s, 1H, =N-NH); ^13^C NMR (DMSO-*d*_6_, 75 MHz) δ = 17.8 (Q-CH_3_), 24.5 (piperidinyl-CH_2_), 25.9 (2C, piperidinyl-CH_2_), 52.2 (2C, piperidinyl-N-CH_2_), 120.9 (C-3), 124.7 (C-6), 124.9 (C-10), 126.4 (C-5), 128.1 (C-3′, C-5′), 128.5 (C-2′, C-6′), 129.9 (C-4′), 130.7 (C-7), 135.1 (C-8), 136.3 (C-4), 138.5 (C-1′), 140.8 (C-9), 146.0 (N=CH), 159.5 (C-2), 176.4 (C=S); Anal. Calcd. for C_23_H_24_ClN_5_S: C, 63.07; H, 5.52; N, 15.99; S, 7.32%. Found: C, 63.00; H, 5.45; N, 15.91; S, 7.26%.

*N*-(2-Fluorophenyl)-2-((8-methyl-2-(piperidin-1-yl)quinolin-3-yl)methylene)hydrazine carbothioamide (**6h**) Yield 96%. Lemon yellow solid. Mp 202–204 °C. FTIR (cm^−1^): 3280 (NH), 3120 (NH), 2928 (CH), 2826 (CH-imine), 1620 (C=N), 1125 (C=S); ^1^H NMR (DMSO-*d*_6_, 300 MHz) δ = 1.61–1.78 (m, 6H, piperidinyl-CH_2_), 2.62 (s, 3H, CH_3_), 3.24–3.28 (m, 4H, piperidinyl-N-CH_2_), 7.25–7.36 (m, 4H, ArH), 7.50–7.52 (m, 2H, ArH), 7.64 (d, *J* = 7.8 Hz, 1H, ArH), 8.35 (s, 1H, ArH), 8.97 (s, 1H, N=CH), 10.09 (s, 1H, NH), 12.28 (s, 1H, =N-NH); ^13^C NMR (DMSO-*d*_6_, 75 MHz) δ = 17.8 (Q-CH_3_), 24.5 (piperidinyl-CH_2_), 25.9 (2C, piperidinyl-CH_2_), 52.3 (2C, piperidinyl-N-CH_2_), 116.3 (C-3′, d, *J* = 19.5 Hz), 120.9 (C-3), 124.6, (C-6′, d, *J* = 6.8 Hz), 124.9 (C-6), 126.3 (C-5), 127.5 (C-10), 127.7 (C-7), 128.9 (C-1′, d, *J* = 8.3 Hz), 130.6 (C-5′), 131.1 (C-4′), 135.1 (C-8), 136.2 (C-4), 140.5 (C-9), 145.9 (N=CH), 159.6 (C-2), 158.0 (C-2′, d, *J* = 245.3 Hz), 177.8 (C=S); Anal. Calcd. for C_23_H_24_FN_5_S: C, 65.53; H, 5.74; N, 16.61; S, 7.61%. Found: C, 65.70; H, 5.88; N, 16.76; S, 7.71%.

*N*-(3-Fluorophenyl)-2-((8-methyl-2-(piperidin-1-yl)quinolin-3-yl)methylene)hydrazine carbothioamide (**6i**) Yield 90%. Yellow solid. Mp 200–202 °C. FTIR (cm^−1^): 3334 (NH), 3137 (NH), 2933 (CH), 2813 (CH-imine), 1601 (C=N), 1163 (C=S); ^1^H NMR (DMSO-*d*_6_, 300 MHz) δ = 1.63–1.78 (m, 6H, piperidinyl-CH_2_), 2.62 (s, 3H, CH_3_), 3.25–3.28 (m, 4H, piperidinyl-N-CH_2_), 7.07 (td, *J* = 8.4, 1.5 Hz, 1H, ArH), 7.31 (t, *J* = 7.5 Hz, 1H, ArH), 7.39–7.53 (m, 3H, ArH), 7.62–7.71 (m, 2H, ArH), 8.37 (s, 1H, ArH), 8.97 (s, 1H, N=CH), 10.28 (s, 1H, NH), 12.26 (s, 1H, =N-NH); ^13^C NMR (DMSO-*d*_6_, 75 MHz) δ = 17.8 (Q-CH_3_), 24.5 (piperidinyl-CH_2_), 25.9 (2C, piperidinyl-CH_2_), 52.3 (2C, piperidinyl-N-CH_2_), 112.4 (C-4′, d, *J* = 21.0 Hz), 112.9 (C-2′, d, *J* = 24.0 Hz), 120.8 (C-3), 122.0 (C-6′, d, *J* = 2.3 Hz), 124.7 (C-6), 124.8 (C-10), 126.4 (C-5), 130.0 (C-5′, d, *J* = 9.0 Hz), 130.7 (C-7), 135.1 (C-8), 136.5 (C-4), 140.9 (C-9), 141.3 (C-1′, d, *J* = 10.5 Hz), 145.9 (N=CH), 159.5 (C-2), 162.0 (C-3′, d, *J* = 240.0 Hz), 176.1 (C=S). Anal. Calcd. for C_23_H_24_FN_5_S: C, 65.53; H, 5.74; N, 16.61; S, 7.61%. Found: C, 65.40; H, 5.61; N, 16.52; S, 7.49%.

*N*-(4-Fluorophenyl)-2-((8-methyl-2-(piperidin-1-yl)quinolin-3-yl)methylene)hydrazine carbothioamide (**6j**) Yield 91%. Yellow solid. Mp 200–202 °C. FTIR (cm^−1^): 3333 (NH), 2954 (CH), 2831 (CH-imine), 1603 (C=N), 1125 (C=S); ^1^H NMR (DMSO-*d*_6_, 300 MHz) δ = 1.63–1.78 (m, 6H, piperidinyl-CH_2_), 2.62 (s, 3H, CH_3_), 3.24–3.28 (m, 4H, piperidinyl-N-CH_2_), 7.21–7.27 (m, 2H, ArH), 7.31 (t, *J* = 7.5 Hz, 1H, ArH), 7.51–7.59 (m, 3H, ArH), 7.67 (d, *J* = 7.8 Hz, 1H, ArH), 8.35 (s, 1H, ArH), 8.98 (s, 1H, N=CH), 10.22 (s, 1H, NH), 12.16 (s, 1H, =N-NH); ^13^C NMR (DMSO-*d*_6_, 75 MHz) δ = 17.8 (Q-CH_3_), 24.5 (piperidinyl-CH_2_), 25.9 (2C, piperidinyl-CH_2_), 52.2 (2C, piperidinyl-N-CH_2_), 115.3 (2C, C-3′, C-5′, d, *J* = 22.5 Hz), 120.9 (C-3), 124.7 (C-6), 124.9 (C-10), 126.3 (C-5), 128.9 (2C, C-2′, C-6′, d, *J* = 8.3 Hz), 130.6 (C-7), 135.1 (C-8), 135.9 (C-1′, d, *J* = 3.0 Hz), 136.3 (C-4), 140.5 (C-9), 145.9 (N=CH), 159.5 (C-2), 160.2 (C-4′, d, *J* = 240.8 Hz), 176.8 (C=S); Anal. Calcd. for C_23_H_24_FN_5_S: C, 65.53; H, 5.74; N, 16.61; S, 7.61%. Found: C, 65.74; H, 5.91; N, 16.82; S, 7.85%.

*N*-(4-Ethylphenyl)-2-((8-methyl-2-(piperidin-1-yl)quinolin-3-yl)methylene)hydrazine carbothioamide (**6k**) Yield 95%. Yellow solid. Mp 198–200 °C. FTIR (cm^−1^): 3272 (NH), 3112 (NH), 2964 (CH), 2914 (CH), 2820 (CH-imine), 1604 (C=N), 1125 (C=S); ^1^H NMR (DMSO-*d*_6_, 300 MHz) δ = 1.20 (t, J = 7.5 Hz, 3H, CH_3_), 1.63–1.78 (m, 6H, piperidinyl-CH_2_), 2.59–2.66 (m, 5H, Ar-CH_3_, -CH_2_CH_3_), 3.25–3.27 (m, 4H, piperidinyl-N-CH_2_), 7.23 (d, *J* = 8.1 Hz, 2H, ArH), 7.30 (t, *J* = 7.5 Hz, 1H, ArH), 7.46–7.52 (m, 3H, ArH), 7.67 (d, *J* = 8.1 Hz, 1H, ArH), 8.35 (s, 1H, ArH), 8.98 (s, 1H, N=CH), 10.14 (s, 1H, NH), 12.09 (s, 1H, =N-NH); ^13^C NMR (DMSO-*d*_6_, 75 MHz) δ = 16.2 (Ar-CH_2_-CH_3_), 17.8 (Q-CH_3_), 24.5 (piperidinyl-CH_2_), 25.9 (2C, piperidinyl-CH_2_), 28.2 (Ar-CH_2_-CH_3_), 52.2 (2C, piperidinyl-N-CH_2_), 121.0 (C-3), 124.6 (C-6), 124.9 (C-10), 126.3 (C-5), 126.6 (2C, C-2′, C-6′), 127.9 (2C, C-3′, C-5′), 130.6 (C-7), 135.1 (C-8), 136.3 (C-4), 137.1 (C-1′), 140.2 (C-9), 141.5 (C-4′), 145.9 (N=CH), 159.5 (C-2), 176.5 (C=S); Anal. Calcd. for C_25_H_29_N_5_S: C, 69.57; H, 6.77; N, 16.23; S, 7.43%. Found: C, 69.65; H, 6.87; N, 16.31; S, 7.50%.

*N*-Benzyl-2-((8-methyl-2-(piperidin-1-yl)quinolin-3-yl)methylene)hydrazinecarbothioamide (**6l**) Yield 91%. Yellow solid. Mp 188–190 °C. FTIR (cm^−1^): 3378 (NH), 3119 (NH), 2967 (CH), 2930 (CH), 2843 (CH-imine), 1602 (C=N), 1110 (C=S); ^1^H NMR (DMSO-*d*_6_, 300 MHz) δ = 1.62–1.76 (m, 6H, piperidinyl-CH_2_), 2.62 (s, 3H, CH_3_), 3.23–3.26 (m, 4H, piperidinyl-N-CH_2_), 4.91 (d, *J* = 6.3 Hz, 2H, N-CH_2_), 7.23–7.42 (m, 6H, ArH), 7.50 (d, *J* = 6.9 Hz, 1H, ArH), 7.65 (d, *J* = 7.8 Hz, 1H, ArH), 8.30 (s, 1H, ArH), 8.82 (s, 1H, N=CH), 9.20 (t, *J* = 6.3 Hz, 1H, NH), 11.92 (s, 1H, =N-NH); ^13^C NMR (DMSO-*d*_6_, 75 MHz) δ = 17.8 (Q-CH_3_), 24.5 (piperidinyl-CH_2_), 25.8 (2C, piperidinyl-CH_2_), 47.1 (Ar-CH_2_-NH), 52.2 (2C, piperidinyl-N-CH_2_), 121.2 (C-3), 124.7 (C-6), 124.8 (C-10), 126.2 (C-5), 127.2 (2C, C-2′, C-6′), 127.7 (2C, C-3′, C-5′), 128.7 (C-4′), 130.5 (C-7), 135.1 (C-8), 135.8 (C-4), 139.9 (C-9, C-1′), 145.9 (N=CH), 159.5 (C-2), 178.0 (C=S); Anal. Calcd. for C_24_H_27_N_5_S: C, 69.03; H, 6.52; N, 16.77; S, 7.68%. Found: C, 69.17; H, 6.70; N, 16.93; S, 7.77%.

2-((8-Methyl-2-(piperidin-1-yl)quinolin-3-yl)methylene)-*N*-(2-morpholinoethyl)hydrazine carbothioamide (**6m**) Yield 90%. Yellow solid. Mp 200–202 °C. FTIR (cm^−1^): 3216 (NH), 3122 (NH), 2929 (CH), 2801 (CH-imine), 1601 (C=N), 1111 (C=S); ^1^H NMR (CDCl_3_, 300 MHz) δ = 1.65–1.69 (m, 2H, piperidinyl-CH_2_), 1.78–1.83 (m, 4H, piperidinyl-CH_2_), 2.70–2.84 (m, 9H, CH_3_, NCH_2_ linked to morpholine nitrogen), 3.29–3.32 (m, 4H, piperidinyl-N-CH_2_), 3.89–3.95 (m, 6H, N-CH_2_, OCH_2_ of morpholine ring), 7.27 (t, *J* = 7.5 Hz, 1H, ArH), 7.49 (d, *J* = 6.9 Hz, 1H, ArH), 7.58 (d, *J* = 7.8 Hz, 1H, ArH), 8.06 (s, 1H, ArH), 8.40 (s, 1H, N=CH), 8.54 (t, *J* = 6.0 Hz, 1H, NH), 9.90 (s, 1H, =N-NH); ^13^C NMR (CDCl_3_, 75 MHz) δ = 17.7 (Q-CH_3_), 24.6 (piperidinyl-CH_2_), 26.0 (2C, piperidinyl-CH_2_), 40.2 (NH-CH_2_-CH_2_-N(morpholine)), 52.3 (2C, piperidinyl-N-CH_2_), 53.2 (NH-CH_2_-CH_2_-N(morpholine)), 56.5 (-N-CH_2_-CH_2_-O(morpholine)), 66.7 (N-CH_2_-CH_2_-O(morpholine)), 119.9 (C-3), 124.4 (C-6), 124.6 (C-10), 125.8 (C-5), 130.5 (C-7), 135.8 (C-4), 140.3 (C-9), 146.5 (N=CH), 159.3 (C-2), 177.0 (C=S); Anal. Calcd. for C_23_H_32_N_6_OS: C, 62.70; H, 7.32; N, 19.07; S, 7.28%. Found: C, 62.55; H, 7.19; N, 18.93; S, 7.09%.

### 3.4. In Vitro Cholinesterase Inhibition Assay

Modified Ellman’s method was adopted to investigate the inhibitory potential of newly synthesized compounds against cholinesterase enzymes [71]. The assay protocol involved 96-well plates (100 μL per well). Each well contained 20 μL of assay buffer solution, 10 μL of test sample, and 10 μL of 0.5 U/mg of AChE (for AChE inhibition assay) or 10 μL of 3.4 U/mg of BChE (for BChE inhibition assay). This mixture was incubated at 25 °C for 10 min followed by the addition of substrate (10 μL). For AChE inhibition assay, acetylthiocholine iodide (ATCI, 1 mM) while for BChE assay, butyrylthiocholine chloride (BTCCl, 1 mM), was added. This mixture was incubated for 15 min at 37 °C. Ellman reagent (5,5′-dithio-bis(2-nitrobenzoic acid), DTNB, 3 mM) was added. The change in color of the mixture showed indication of inhibition. The absorbance was measured at 405 nm using microplate reader BioTek Elx800TM, Inc. USA. Blank assay was also performed. All the analyses were performed in triplicate. IC_50_ values were also calculated for the synthesized compounds in GraphPad prism 5.0. using nonlinear regression method.

### 3.5. Molecular Docking Studies

Initially 3D structures of synthesized compounds and cognate ligands were drawn and protonated with the help of molecular operating environment (MOE) [72]. Energy minimization of selected compounds was performed with the help of MMFF94x forcefield (New Jersey, USA) through adjustment of hydrogens [73]. The crystal structure of human AChE (PDB ID: 4BDT) [66] in complex with huprine W and BChE (PDB ID: 4BDS) [66] in complex with tacrine were obtained from the RCSB Protein Bank. The docking method was able to reproduce the experimentally bound conformation of ligand in the active site with an RMSD of <1.0 Å. Active sites within AChE and BChE receptor were carefully chosen around 9.0 and 10.5 Å radius of cocrystallized ligand, respectively. The solvent handling and amino acid flip parameters were set as default. With the help of LeadIT program [67], the best 50 scoring docked poses were nominated for further analysis. For visual analysis, the Discovery Studio Visualizer v19 was employed [74]. The mode of binding of docked poses was evaluated with the help of HYDE assessment tool of LeadIT [75]. Binding free energy (∆G) for each pose was determined to explore the degree of interaction with receptor. The minimum energy value reflects high stability and affinity of molecules to bind with receptor.

### 3.6. Cytotoxicity

#### 3.6.1. Sampling of Cell Lines

HepG2 cell line was obtained from the cell culture laboratory established in The University of Lahore. These cell lines were preserved in cryo vials present in liquid nitrogen. Cryo vials were revived for further processing.

#### 3.6.2. Culturing of Cell Lines

The cryo vials obtained from liquid nitrogen cylinder were thawed. Then HepG2 cells were cultured in the culturing flask in which DMEM-HG along with 10% fetal bovine serum (FBS), supplemented with 100 mg/mL penicillin G (Sigma) and 100 U/mL streptomycin (Sigma) were added. Cultures were maintained in a humidified incubator supplied with 5% CO_2_ at 37 °C. Experiments were performed in triplicates. When cultured HepG2 cells achieved 70–80% confluence their subculturing was conducted. For splitting, the cells attached to the walls of the culturing flask were washed with 1× phosphate buffer saline (PBS) and incubated with 0.05% trypsin-EDTA until cells detached from the surface of culturing flask. The detachment of the cells was confirmed by observing the flask under the inverted microscope. A few drops of FBS were added to the flask and mixed well by stirring. The mixture was centrifuged at 2000 rpm for 5 min. After centrifugation the supernatant was removed, and pellet was resuspended. HepG2 cells were cultured onto 96-well plates for measurement of cell viability. Treatment was given for both compounds at a concentration of 0–1000 µg/mL for 24 h.

#### 3.6.3. Cytotoxicity Calculation via MTT Assay 

After 24 h of treatment with different concentrations of compounds **5** and **6**, cells were washed with phosphate buffer saline (PBS) (Invitrogen Inc., Carlsbad, CA, USA), and then incubated with 100 µL of DMEM and 25 µL MTT solution (Invitrogen Inc.) for 3–4 h. After 4 h formazan crystals were solubilized with 10% sodium dodecylsulphate (SDS) (Invitrogen Inc.) and absorbance was taken at 570 nm [76]. Percentage viability was calculated according to the following formula:

*% Viable cells* = ((*abs _sample_ − abs _blank_*) / (*abs _control_ − abs _blank_*)) × 100 (1)

where *abs* stands for absorbance.

Experiments were repeated three times for the average calculations.

## 4. Conclusions

In summary, the present study described an efficient multistep synthetic route for the preparation of a library of 2-((6/8-methyl-2-(piperidin-1-yl)quinolin-3-yl)methylene)hydrazinecarbothioamides. The synthesized compounds contain a substantial degree of structural variation in the form of electron-donating as well as electron-withdrawing groups on different positions of the aromatic rings. Both quinoline motif and thiosemicarbazone moiety were endowed with a variety of functional groups. The synthesized hybrid thiosemicarbazones were evaluated for their inhibitory potential against AChE and BChE enzymes. Compounds possessing an 8-methyl substituted quinoline ring were found to be more effective cholinergic inhibitors with relatively lower IC_50_ values as compared to the 6-substituted analogues. Several compounds displayed good inhibitory activity among which **6d**, **6f**, and **6g** were identified as potent dual inhibitors of AChE and BChE with IC_50_ < 20 μM. The hybrid thiosemicarbazone **6f** was concluded as the most potent dual inhibitor against both the enzymes (IC_50_ = 9.68 μM for AChE and IC_50_ = 11.59 μM for BChE). Moreover, compound **6i** appeared to be the selective inhibitor of AChE with an IC_50_ value of 15.8 ± 1.3 μM. In vitro biochemical assay results were rationalized using molecular docking approach where the binding site analysis of potent compounds revealed similar interactions to cognate ligands within the active sites of enzymes. Furthermore, these compounds have also shown weak to moderate cytotoxic activity. Physicochemical properties, lipophilicity, water solubility, pharmacokinetics, drug-likeness, and medicinal chemistry properties were also calculated for the synthesized hybrid scaffold suggesting the safer profile to be investigated as drug molecules and have high probability of blood–brain penetration and absorption. Collectively, the identification of these *N*-heterocyclic hybrid molecules presents significant implications for the design of new AChE and BChE inhibitors. Further alterations in the structural framework of these compounds could be a determining factor to improve their anticholinergic potential which may complement the drug-discovery process against Alzheimer’s disease.

## Figures and Tables

**Table 1:** Comparative synthetic yields and anti-cholinesterase (AChE and BChE) potential of synthesized compounds **5(a**–**m)** and **6(a**–**m)**.

Compound	Conventional Approach	Microwave-Assisted Approach	Acetylcholinesterase (AChE)	Butyrylcholinesterse (BChE)
	Yield (%)		IC_50_ ± SEM (μM)	
**5a**	80	91	27.6 ± 0.08	35.3 ± 0.01
**5b**	86	92	25.1 ± 0.25	33.1 ± 0.09
**5c**	85	92	42.3 ± 0.99	37.2 ± 0.83
**5d**	90	95	25.9 ± 0.96	29.8 ± 1.2
**5e**	85	91	35.2 ± 0.54	40.26 ± 0.17
**5f**	94	97	23.9 ± 0.25	24.6 ± 0.57
**5g**	93	97	19.85 ± 0.14	23.1 ± 0.11
**5h**	80	90	31.28 ± 0.37	25.12 ± 0.99
**5i**	90	96	24.09 ± 0.43	36.23 ± 0.02
**5j**	83	90	28.21 ± 0.94	42.36 ± 0.44
**5k**	94	97	30.65 ± 0.56	32.01 ± 0.87
**5l**	91	95	35.09 ± 1.2	45.02 ± 0.38
**5m**	78	89	62.3 ± 0.68	59.35 ± 0.13
**6a**	81	90	36.25 ± 0.36	60.02 ± 0.04
**6b**	89	93	25.89 ± 0.45	28.77 ± 0.63
**6c**	85	91	39.12 ± 0.27	24.59 ± 0.09
**6d**	92	98	12.89 ± 0.33	17.86 ± 0.35
**6e**	89	93	32.11 ± 0.15	37.06 ± 0.59
**6f**	88	93	9.68 ± 0.21	11.59 ± 1.2
**6g**	92	95	13.85 ± 0.78	18.56 ± 0.22
**6h**	92	96	40.23 ± 0.25	45.12 ± 0.19
**6i**	87	90	15.8 ± 1.3	51.11 ± 0.28
**6j**	83	91	56.66 ± 0.41	51.03 ± 0.52
**6k**	90	95	39.91 ± 0.85	32.01 ± 0.31
**6l**	82	91	57.25 ± 0.03	41.02 ± 0.89
**6m**	82	90	21.01 ± 2.3	35.14 ± 0.77
**Donepezil**	―	―	2.98 ± 0.62	7.21 ± 0.39

**Table 2:** Docking score of the top pose of selected compounds and their ranks after HYDE visual inspection in the AChE and BChE.

Compounds	Acetylcholinesterase		Butyrylcholinesterase	
	Docking Score by FlexX for Top Pose (kcal mol^−1^)	Free Energy of Binding ΔG (kJ mol^−1^)	Docking Score by FlexX for Top Pose (kcal mol^−1^)	Free Energy of Binding ΔG (kJ mol^−1^)
**5a**	−23.1892	−15	−27.4250	−13
**5b**	−27.3759	−18	−33.8532	−18
**5c**	−15.1888	−15	−38.2179	−19
**5d**	−34.0828	−17	−36.4835	−20
**5e**	−21.2287	−19	−35.5422	−17
**5f**	−30.9532	−23	−32.4958	−21
**5g**	−24.9478	−22	−31.3943	−20
**5h**	−25.3819	−14	−33.4593	−22
**5i**	−26.3914	−25	−31.8917	−18
**5j**	−27.7381	−13	−33.3263	−19
**5k**	−18.8869	−10	−31.3938	−17
**5l**	−23.4789	−11	−30.7439	−14
**5m**	−25.4734	−17	−29.6099	−13
**6a**	−25.2746	−14	−28.9743	−17
**6b**	−24.0306	−13	−32.3915	−14
**6c**	−14.5216	−10	−34.1889	−22
**6d**	−21.9230	−12	−34.6624	−20
**6e**	−26.5830	−15	−32.5630	−19
**6f**	−25.5518	−24	−31.0974	−20
**6g**	−25.8729	−24	−28.6461	−19
**6h**	−24.8538	−15	−28.7235	−14
**6i**	−26.5146	−26	−31.4126	−18
**6j**	−30.6048	−14	−29.3584	−17
**6k**	−19.5944	−16	−27.7883	−11
**6l**	−22.5508	−11	−27.0438	−16
**6m**	−28.8842	−17	−28.7614	−15
**Huprine W**	−16.29	−23	―	―
**Tacrine**	―	―	−17.70	−18

**Table 3:** ADMET prediction scores for the selected compounds [68].

Properties	Compounds							
	5f	5g	5h	5i	6c	6f	6g	6i
**Physicochemical properties**								
Mol. Wt. (g/mol)	437.99	437.99	421.53	421.53	431.60	437.99	437.99	421.53
No. of atoms	30	30	30	30	31	0	30	30
No. of aromatic atoms	16	16	16	16	16	16	16	16
Fraction C_sp3_	0.26	0.26	0.26	0.26	0.32	0.26	0.26	0.26
No. of rotatable bonds	6	6	6	6	6	6	6	6
No. of H-bond acceptors	2	2	3	3	2	2	2	3
No. of H-bond donors	2	2	2	2	2	2	2	2
Molar refractivity	134.05	134.05	128.99	128.99	138.97	134.05	134.05	128.99
TPSA	84.64	84.64	84.64	84.64	84.64	84.64	84.64	84.64
**Lipophilicity**								
Log *P*_o/w_ (iLOGP)	4.45	4.45	4.28	4.25	4.43	3.96	4.38	4.00
Log *P*_o/w_ (XLOGP3)	5.72	5.72	5.19	5.19	5.82	5.72	5.72	5.19
Log *P*_o/w_ (WLOGP)	4.94	4.94	4.84	4.84	4.90	4.94	4.94	4.84
Log *P*_o/w_ (MLOGP)	4.13	4.13	4.02	4.02	4.07	4.13	4.13	4.02
Log *P*_o/w_ (SILICOS-IT)	5.84	5.84	5.62	5.62	6.26	5.84	5.84	5.62
Consensus Log *P*_o/w_	5.02	5.02	4.79	4.78	5.10	4.92	5.00	4.74
**Water solubility**								
Log S (ESOL)	−6.16	−6.16	−5.72	−5.72	−6.17	−6.16	−6.16	−5.72
Solubility (mg/mL; mol/L)	3.05 × 10^−^^4^; 6.95 × 10^−7^	3.05 × 10^−^^4^; 6.95 × 10^−7^	8.00 × 10^−^^4^; 1.90 × 10^−6^	8.00 × 10^−^^4^; 1.90 × 10^−6^	2.93 × 10^−^^4^; 6.79 × 10^−7^	3.05 × 10^−^^4^; 6.95 × 10^−7^	3.05 × 10^−^^4^; 6.95 × 10^−7^	8.00 × 10^−^^4^; 1.90 × 10^−6^
Class	Poorly soluble	Poorly soluble	Moderately soluble	Moderately soluble	Poorly soluble	Poorly soluble	Poorly soluble	Moderately soluble
Log S (ALi)	−7.26	−7.26	−6.71	−6.71	−7.37	−7.26	−7.26	−6.71
Solubility (mg/mL; mol/L)	2.38 × 10^−5^; 5.44 × 10^−8^	2.38 × 10^−5^; 5.44 × 10^−8^	8.14 × 10^−5^; 1.93 × 10^−7^	8.14 × 10^−5^; 1.93 × 10^−7^	1.85 × 10^−5^; 4.28 × 10^−8^	2.38 × 10^−5^; 5.44 × 10^−8^	2.38 × 10^−5^; 5.44 × 10^−8^	8.14 × 10^−5^; 1.93 × 10^−7^
Class	Poorly soluble	Poorly soluble	Poorly soluble	Poorly soluble	Poorly soluble	Poorly soluble	Poorly soluble	Poorly soluble
Log S (SILICOS-IT)	−8.18	−8.18	−7.86	−7.86	−8.35	−8.18	−8.18	−7.86
Solubility (mg/mL; mol/L)	2.89 × 10^−6^; 6.59 × 10^−9^	2.89 × 10^−6^; 6.59 × 10^−9^	5.82 × 10^−6^; 1.38 × 10^−8^	5.82 × 10^−6^; 1.38 × 10^−8^	1.95 × 10^−6^; 4.51 × 10^−9^	2.89 × 10^−6^; 6.59 × 10^−9^	2.89 × 10^−6^; 6.59 × 10^−9^	5.82 × 10^−6^; 1.38 × 10^−8^
Class	Poorly soluble	Poorly soluble	Poorly soluble	Poorly soluble	Poorly soluble	Poorly soluble	Poorly soluble	Poorly soluble
**Pharmacokinetics**								
GI absorption	High	High	High	High	High	High	High	High
BBB permeant	No	No	No	No	No	No	No	No
P-gp substrate	No	No	No	No	Yes	No	No	No
CYP1A2 inhibitor	No	No	No	No	No	No	No	No
CYP2C19 inhibitor	Yes	Yes	Yes	Yes	Yes	Yes	Yes	Yes
CYP2C9 inhibitor	Yes	Yes	Yes	Yes	Yes	Yes	Yes	Yes
CYP2D6 inhibitor	No	No	No	No	No	No	No	No
CYP3A4 inhibitor	Yes	Yes	Yes	Yes	Yes	Yes	Yes	Yes
Log *K*_p_ (skin permeation) (cm/s)	−4.91	−4.91	−5.19	−5.19	−4.80	−4.91	−4.91	−5.19
**Drug-likeness**								
Lipinski	Yes; 0 violation	Yes; 0 violation	Yes; 0 violation	Yes; 0 violation	Yes; 0 violation	Yes; 1 violation: MLOGP > 4.15	Yes; 0 violation	Yes; 1 violation: MLOGP > 4.15
Ghose	No; 1 violation: MR > 130	No; 1 violation: MR > 130	Yes; 0 violation	Yes; 0 violation	No; 1 violation: MR > 130	No; 1 violation: MR > 130	No; 1 violation: MR > 130	Yes; 0 violation
Veber	Yes; 0 violation	Yes; 0 violation	Yes; 0 violation	Yes; 0 violation	Yes; 0 violation	Yes; 0 violation	Yes; 0 violation	Yes; 0 violation
Egan	Yes; 0 violation	Yes; 0 violation	Yes; 0 violation	Yes; 0 violation	Yes; 0 violation	Yes; 0 violation	Yes; 0 violation	Yes; 0 violation
Muegge	No; 1 violation: XLOGP3 > 5	No; 1 violation: XLOGP3 > 5	No; 1 violation: XLOGP3 > 5	No; 1 violation: XLOGP3 > 5	No; 1 violation: XLOGP3 > 5	No; 1 violation: XLOGP3 > 5	No; 1 violation: XLOGP3 > 5	No; 1 violation: XLOGP3 > 5
Bioavailability score	0.55	0.55	0.55	0.55	0.55	0.55	0.55	0.55
**Medicinal chemistry**								
PAINS	0 alert	0 alert	0 alert	0 alert	0 alert	0 alert	0 alert	0 alert
Brenk	2 alerts: imine_1, thiocarbonyl_group	2 alerts: imine_1, thiocarbonyl_group	2 alerts: imine_1, thiocarbonyl_group	2 alerts: imine_1, thiocarbonyl_group	2 alerts: imine_1, thiocarbonyl_group	2 alerts: imine_1, thiocarbonyl_group	2 alerts: imine_1, thiocarbonyl_group	2 alerts: imine_1, thiocarbonyl_group
Lead-likeness	No; 1 Violation; XLOGP3 > 3.5	No; 1 Violation; XLOGP3 > 3.5	No; 1 Violation; XLOGP3 > 3.5	No; 1 Violation; XLOGP3 > 3.5	No; 1 Violation; XLOGP3 > 3.5	No; 1 Violation; XLOGP3 > 3.5	No; 1 Violation; XLOGP3 > 3.5	No; 2 Violations; XLOGP3 > 3.5
Synthetic accessibility	3.36	3.35	3.47	3.40	3.62	3.39	3.38	3.43

**Table 4:** Cytotoxicity results of the newly synthesized compounds.

Cell Viability at Concentrations (μg/mL)							
Compound	Control	50 μg/mL	100 μg/mL	200 μg/mL	500 μg/mL	1000 μg/mL	Status
**5a**	100	95.4	88.9	92.8	95.8	102	Non-cytotoxic
**5b**	100	106	107	139	169	227	Proliferative
**5c**	100	105	109	122	188	262	Proliferative
**5d**	100	105	108	110	134	131	Proliferative
**5e**	100	94.2	97.2	158	196	76.3	Cytotoxic
**5f**	100	84.8	94.9	93.9	93.2	56.9	Cytotoxic
**5g**	100	84.3	96.4	94.1	122	135	Proliferative
**5h**	100	113	121	83.8	151	209	Proliferative
**5i**	100	118	99.4	97.2	99.4	149	Proliferative
**5j**	100	93.4	98.1	107	138	166	Proliferative
**5k**	100	111	110	99.6	106	132	Proliferative
**5l**	100	101	101	107	109	133	Proliferative
**5m**	100	94.0	102	109	107	115	Non-cytotoxic
**6a**	100	82.5	86.3	80.6	83.3	77.9	Cytotoxic
**6b**	100	89.91	95.72	92.82	112.3	69.21	Cytotoxic
**6c**	100	87.46	115.7	153.7	186.7	233.1	Proliferative
**6d**	100	75.00	93.87	91.26	128.7	173.4	Proliferative
**6e**	100	102.2	112.5	167.4	179.7	212.1	Proliferative
**6f**	100	109.7	110.1	108.8	139.0	188.7	Proliferative
**6g**	100	113.1	106.3	110.7	149.5	214.1	Proliferative
**6h**	100	100.4	110.0	125.6	151.1	201.9	Proliferative
**6i**	100	95.55	102.5	131.4	112.2	186.2	Proliferative
**6j**	100	102.9	93.71	94.81	107.9	114.4	Non-cytotoxic
**6k**	100	103.2	104.6	107.3	137.7	156.6	Proliferative
**6l**	100	103.2	104.6	107.3	137.7	156.6	Proliferative
**6m**	100	97.87	96.75	95.29	97.41	87.58	Cytotoxic

## Data Availability

The data presented in this study are available in supplementary material.

## Supplementary Materials

The following are available online, ^1^H and ^13^C NMR spectra of all the synthesized compounds and graphical representation of the cytotoxicity results.

## Sample Availability

Samples of the synthetic compounds are available from the authors on reasonable request.
