# Supplementary material for: Microwave-Assisted Synthesis of (Piperidin-1-yl)quinolin-3-yl)methylene)hydrazinecarbothioamides as Potent Inhibitors of Cholinesterases: A Biochemical and In Silico Approach
Source: Molecules. 2021 Jan 27;26(3):656. doi: 10.3390/molecules26030656 (PMC7866225; doi:10.3390/molecules26030656)
Supplement: Supplementary file 1 [file molecules-26-00656-s001.pdf]

molecules

MDPI

Supporting Information

Microwave-assisted synthesis of (piperi-din-1-yl)quinolin-3-yl)methylene)hydrazinecarbothioamides as potent inhibitors of cholinesterases: A biochemical and in silico approach

Rubina Munir [1,*], Muhammad Zia-ur-Rehman [2,*], Shahzad Murtaza [3], Sumera Zaib [4], Noman Javid [1,5], Sana Javaid Awan [6,7], Kiran Iftikhar [3], Muhammad Makshoof Athar [1] and Imtiaz Khan [8,*]

1. Institute of Chemistry, University of the Punjab, Lahore 54590, Pakistan; organist94@gmail.com (R.M.); atharmakshoof@gmail.com (M.M.A.)
2. Applied Chemistry Research Centre, PCSIR Laboratories Complex, Lahore 54600, Pakistan; rehman_pcsir@yahoo.com (M.Z.R.)
3. Department of Chemistry, University of Gujrat, Gujrat, Pakistan; shahzad.murtaza@uog.edu.pk (S.M.); kiran.iffi@gmail.com (K.I.)
4. Department of Biochemistry, Faculty of Life Sciences, University of Central Punjab, Lahore 54590, Pakistan; sumera.biochem@gmail.com (S.Z.)
5. Department of Chemistry (C-Block), Forman Christian College, Ferozepur Road Lahore, Pakistan; noumanjavid@gmail.com (N.J.)
6. The University of Lahore, Lahore, Pakistan; sana.javaidawan@yahoo.com (S.J.A.)
7. Department of Zoology, Kinnaird College for Women, Lahore 54000, Pakistan
8. Department of Chemistry and Manchester Institute of Biotechnology, The University of Manchester, 131 Princess Street, Manchester M1 7DN, UK; imtiaz.khan@manchester.ac.uk (I.K.)

Correspondence: organist94@gmail.com (R.M.); rehman_pcsir@yahoo.com (M.Z.R.); imtiaz.khan@manchester.ac.uk (I.K.)

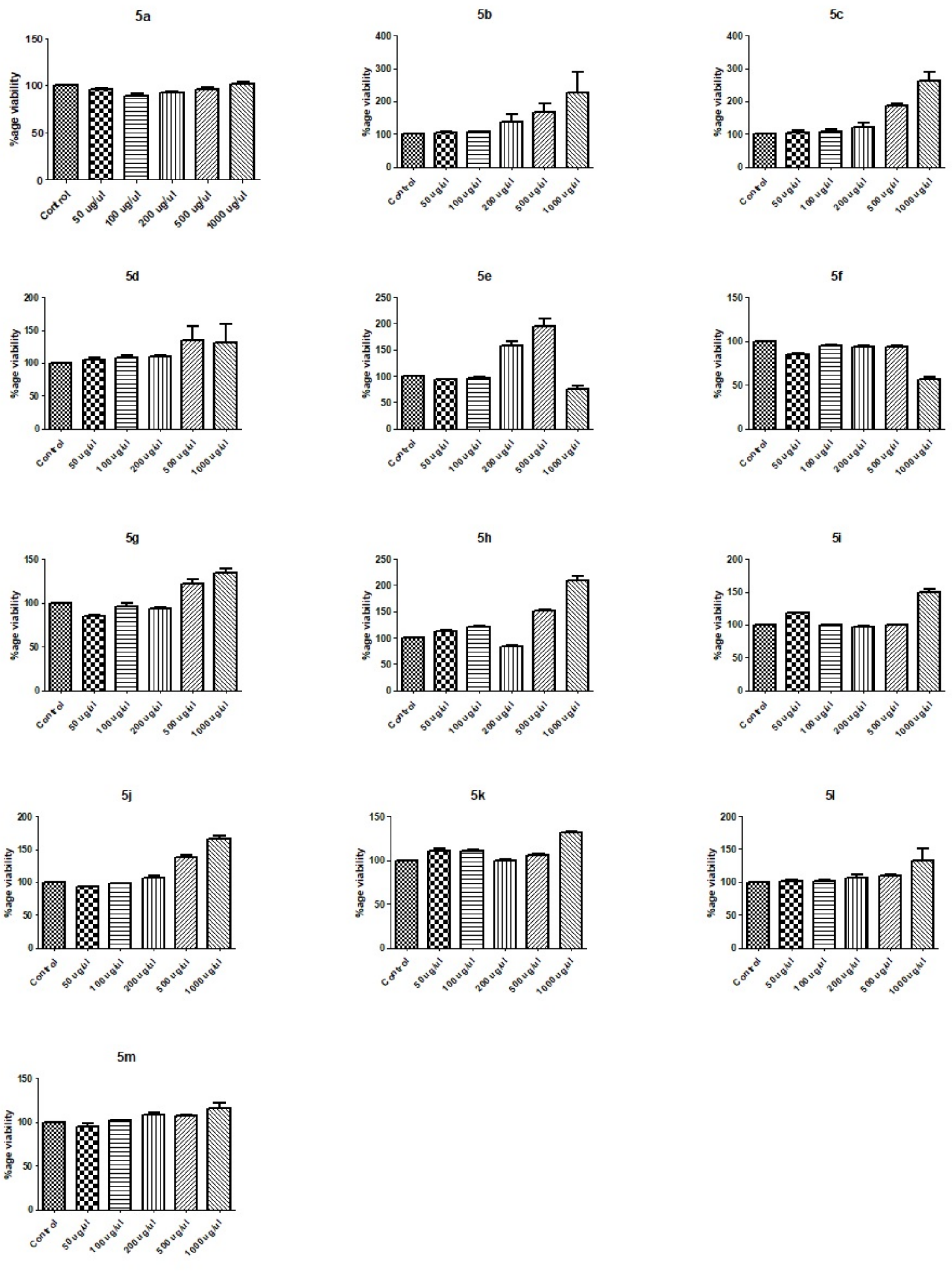

Figure S1. Graphical representation of the cytotoxicity of compound **5(a-m)** at a concentration of 0–1000 μg/mL. Graph were constructed by using graph pad prism 5 considering $p > 0.05$.

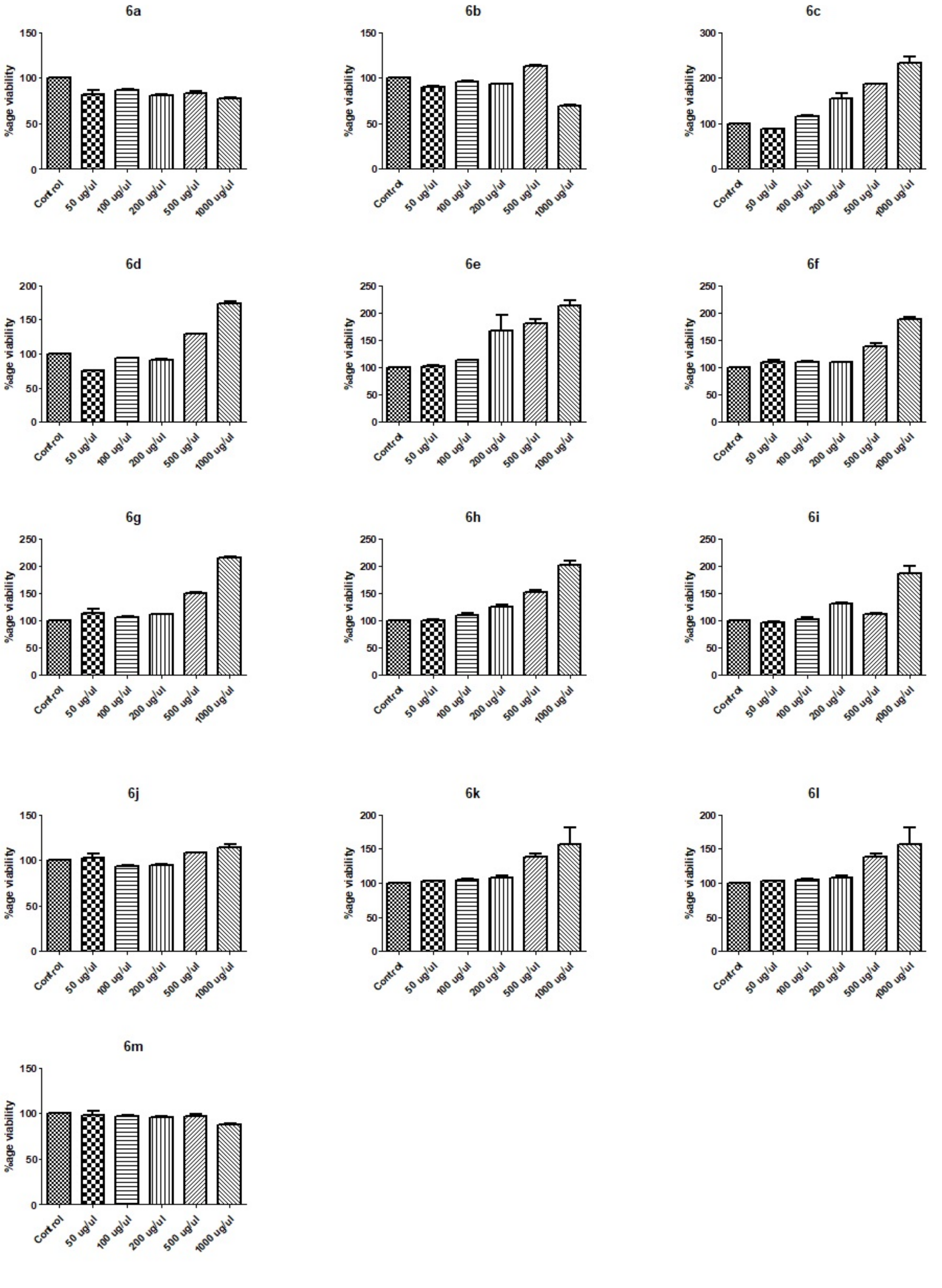

Figure S2. Graphical representation of the cytotoxicity of compound **6(a-m)** at a concentration of 0–1000 μg/mL. Graph were constructed by using graph pad prism 5 considering $p > 0.05$.

1. Preparation of 2-chloroquinoline-3-carbaldehydes 3(a,b)

2-Chloroquinoline-3-carbaldehydes **3(a,b)** were prepared by using method reported by Meth-Cohn and coworkers [1]. $POCl_3$ (65.3 mL, 107.45 g, 0.70 mol) was added drop-wise to DMF (19.3 mL, 18.26 g, 0.25 mol) with constant stirring while maintaining the

temperature of the flask at 0 °C. To the resulting Vilsmeyer reagent, acetanilide **2** (0.10 mol) was added and the reaction mixture was heated at 70–80 °C. The progress of the reaction was monitored thin layer chromatography (TLC). The reaction mixture was then poured on crushed ice (500 g) cautiously and stirred vigorously at 0–10 °C. The precipitated 2-chloroquinoline-3-carbaldehyde **3** was filtered, washed with excess water, dried, and recrystallized from ethyl acetate.

2. Preparation of piperidinyl quinoline-3-carbaldehydes (4a,b)

Piperidine (11 mmol) was added to a stirred solution of 2-chloro-6-methylquinoline-3-carbaldehyde **3a** or 2-chloro-8-methylquinoline-3-carbaldehyde **3b** (10 mmol) and catalytic amount of cetyltrimethylammonium bromide (CTAB) in PEG-400 (10 mL). The resulting reaction mixture was heated at 135 °C for 2.5 h. After cooling to room temperature, the reaction mixture was poured onto crushed ice and stirred overnight. The yellow precipitates were filtered, washed with water, dried and recrystallized from ethanol [2].

3. 6-Methyl-2-(piperidin-1-yl)quinoline-3-carbaldehyde (4a) Yield 98%.

Yellow solid. Mp 90–92 °C (lit. 91–93 °C) [3]. FTIR (cm^{-1}) 3030 (CH-aromatic), 2936 (CH), 2852 (CH-formyl), 1691 (C=O), 1572 (C=N, aromatic), 1053 (C-N); ^1H NMR ($CDCl_3$, 300 MHz) δ = 1.66–1.73 (m, 2H, piperidinyl-CH_2), 1.77-1.84 (m, 4H, piperidinyl-CH_2), 2.51 (s, 3H, CH_3), 3.41-3.45 (m, 4H, piperidinyl-N-CH_2), 7.52 (d, J = 1.8 Hz, 1H, ArH), 7.54–7.56 (m, 1H, ArH), 7.75 (d, J = 8.4 Hz, 1H, ArH), 8.42 (s, 1H, ArH), 10.18 (s, 1H, O=CH); ^{13}C NMR (DMSO-d_6, 75 MHz) δ = 21.2 (Q-$\underline{C}H_3$), 24.5 (piperidinyl-$\underline{C}H_2$), 25.8 (2C, piperidinyl-$\underline{C}H_2$), 52.2 (piperidinyl-N-$\underline{C}H_2$), 122.5 (C-3), 123.9 (C-10), 127.1 (C-8), 128.7 (C-5), 134.0 (C-6), 135.0 (C-7), 142.0 (C-4), 147.5 (C-9), 159.0 (C-2), 190.8 (C=O); Anal. Calcd. for $C_{16}H_{18}N_2O$: C, 75.56; H, 7.13; N, 11.01%, Found: C, 75.79; H, 4.19; N, 11.12%.

4. 8-Methyl-2-(piperidin-1-yl)quinoline-3-carbaldehyde (4b)

Yield 97%. Yellow solid. Mp 82–84 °C (lit. 83–85 °C) [3]. FTIR (cm^{-1}): 3023 (CH-aromatic), 2928 (CH), 2851 (CH-formyl), 1687 (C=O), 1569 (C=N, aromatic), 1050 (C-N); ^1H NMR ($CDCl_3$, 300 MHz) δ = 1.69–1.76 (m, 2H, piperidinyl-CH_2), 1.80–1.85 (m, 4H, piperidinyl-CH_2), 2.70 (s, 3H, CH_3), 3.48–3.51 (m, 4H, piperidinyl-N-CH_2), 7.26 (t, J = 7.5 Hz, 1H, ArH), 7.56 (d, J = 6.9 Hz, 1H, ArH), 7.64 (d, J = 8.1 Hz, 1H, ArH), 8.47 (s, 1H, ArH), 10.18 (s, 1H, O=CH); ^{13}C NMR ($CDCl_3$, 75 MHz) δ = 17.7 (Q-$\underline{C}H_3$), 24.6 (piperidinyl-$\underline{C}H_2$), 25.9 (2C, piperidinyl-$\underline{C}H_2$), 52.5 (2C, piperidinyl-N-$\underline{C}H_2$), 121.7 (C-3), 123.6 (C-6), 124.0 (C-10), 127.1 (C-5), 132.3 (C-7), 135.6 (C-8), 141.6 (C-4), 148.2 (C-9), 158.9 (C-2), 190.8 (C=O); Anal. Calcd. for $C_{16}H_{18}N_2O$: C, 75.56; H, 7.13; N, 11.01%, Found: C, 75.83; H, 4.32; N, 11.19%.

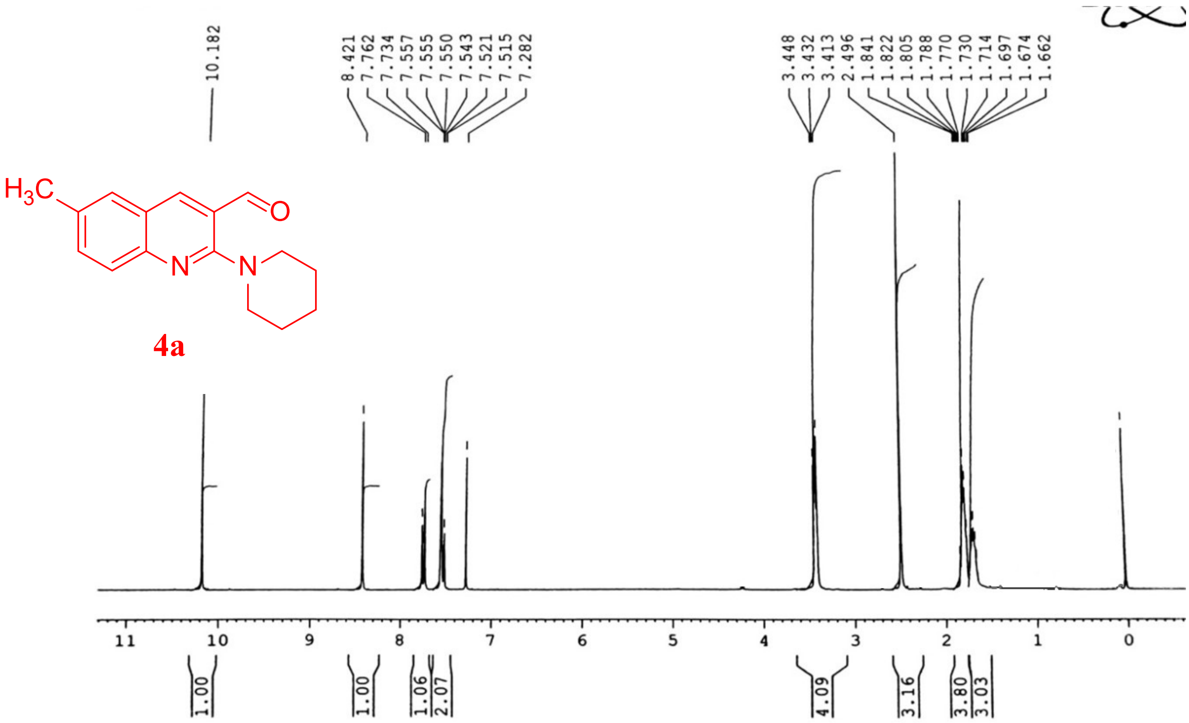

10.182
8.421
7.762
7.734
7.557
7.555
7.550
7.543
7.521
7.515
7.282
3.448
3.432
3.413
2.496
1.841
1.822
1.805
1.788
1.770
1.730
1.714
1.697
1.674
1.662
H3C
N
N
O
4a
11
10
9
8
7
6
5
4
3
2
1
0
1.00
1.00
1.06
2.07
4.09
3.16
3.80
3.03

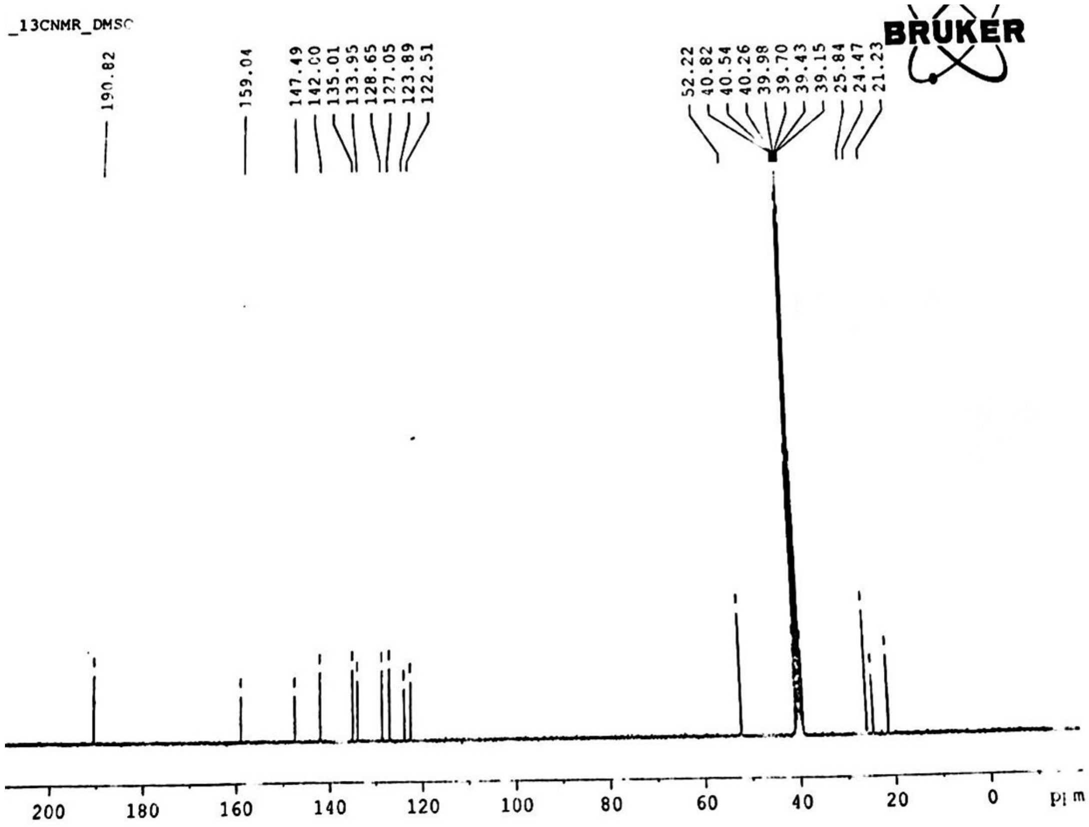

Figure S3. NMR Spectra of 6/8-methyl-2-(piperidin-1-yl)quinoline-3-carbaldehydes (**4a** and **4b**).

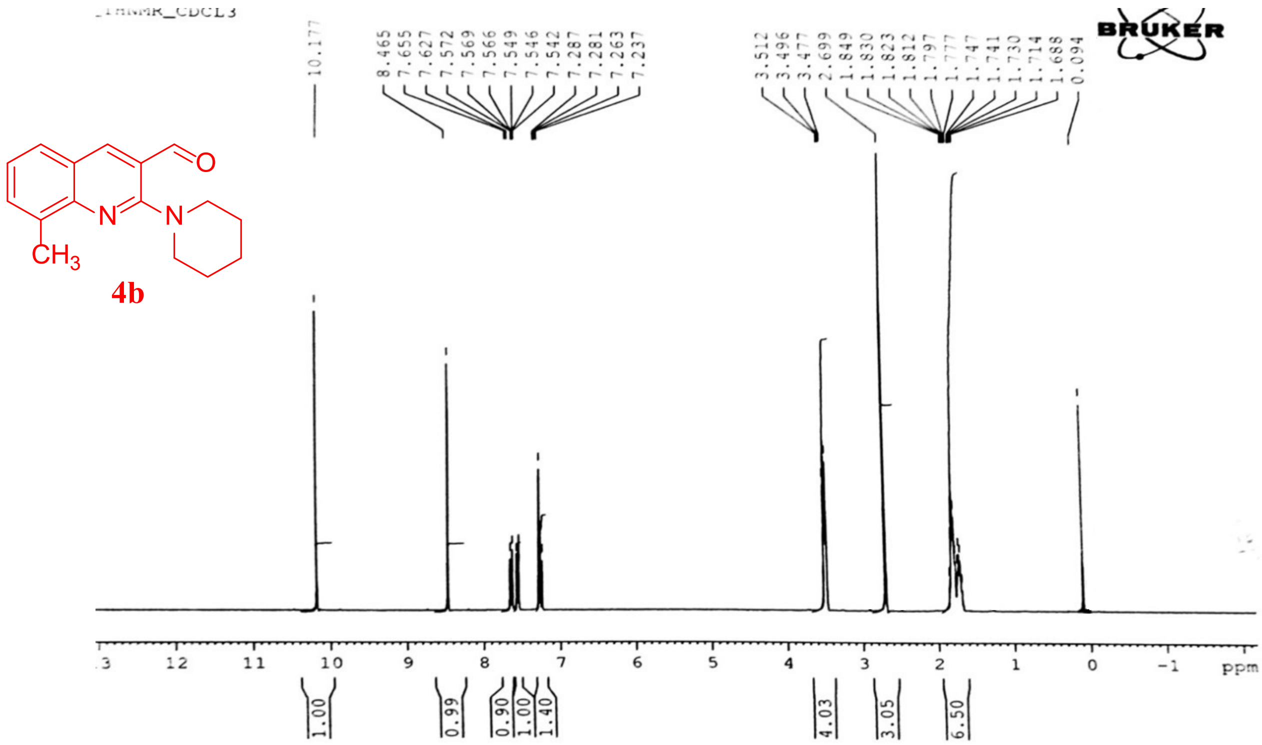

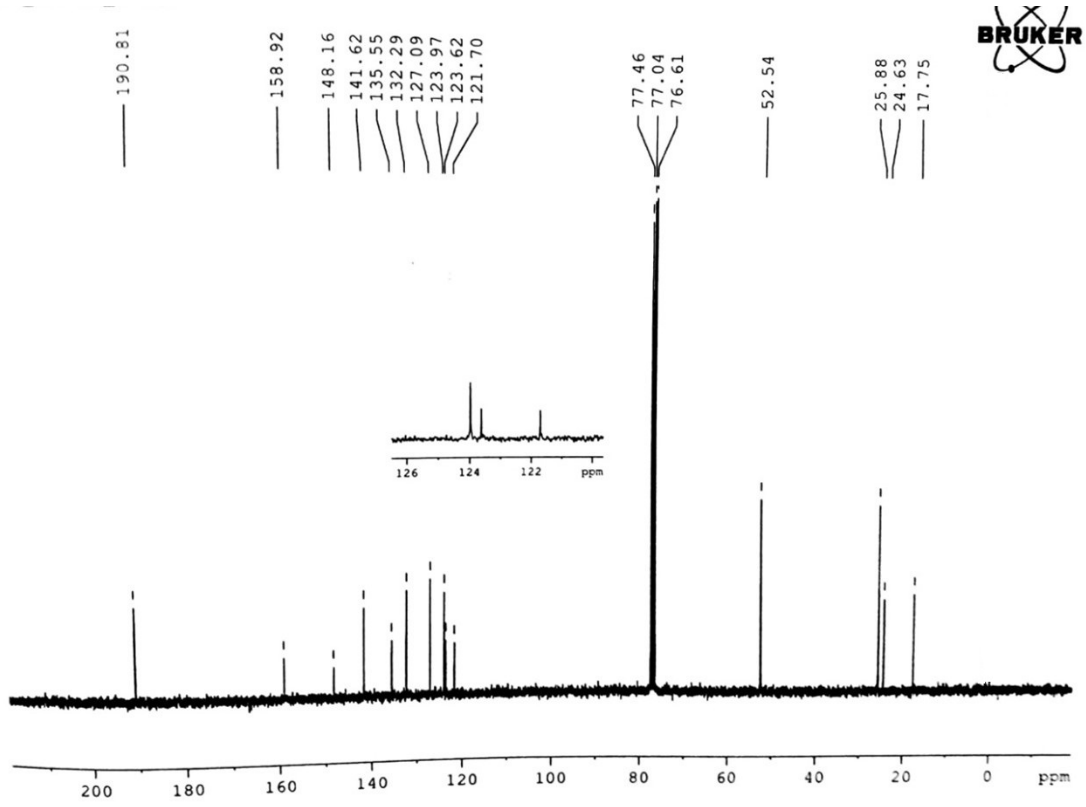

Figure S4. NMR spectra of thiosemicarbazones **5(a-m).**

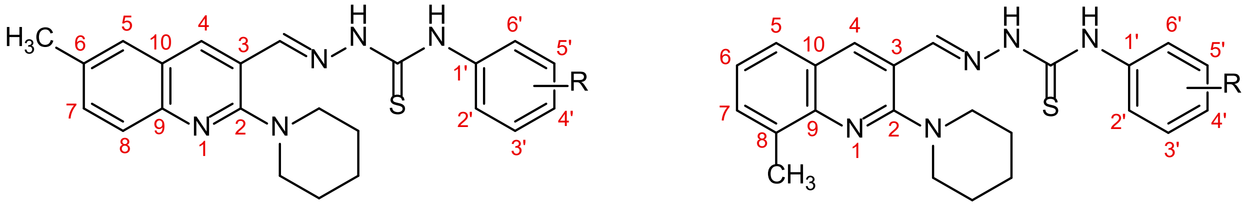

Figure S5. Numbering of Carbon atoms for Compounds **5(a-m)** and **6(a-m)**.

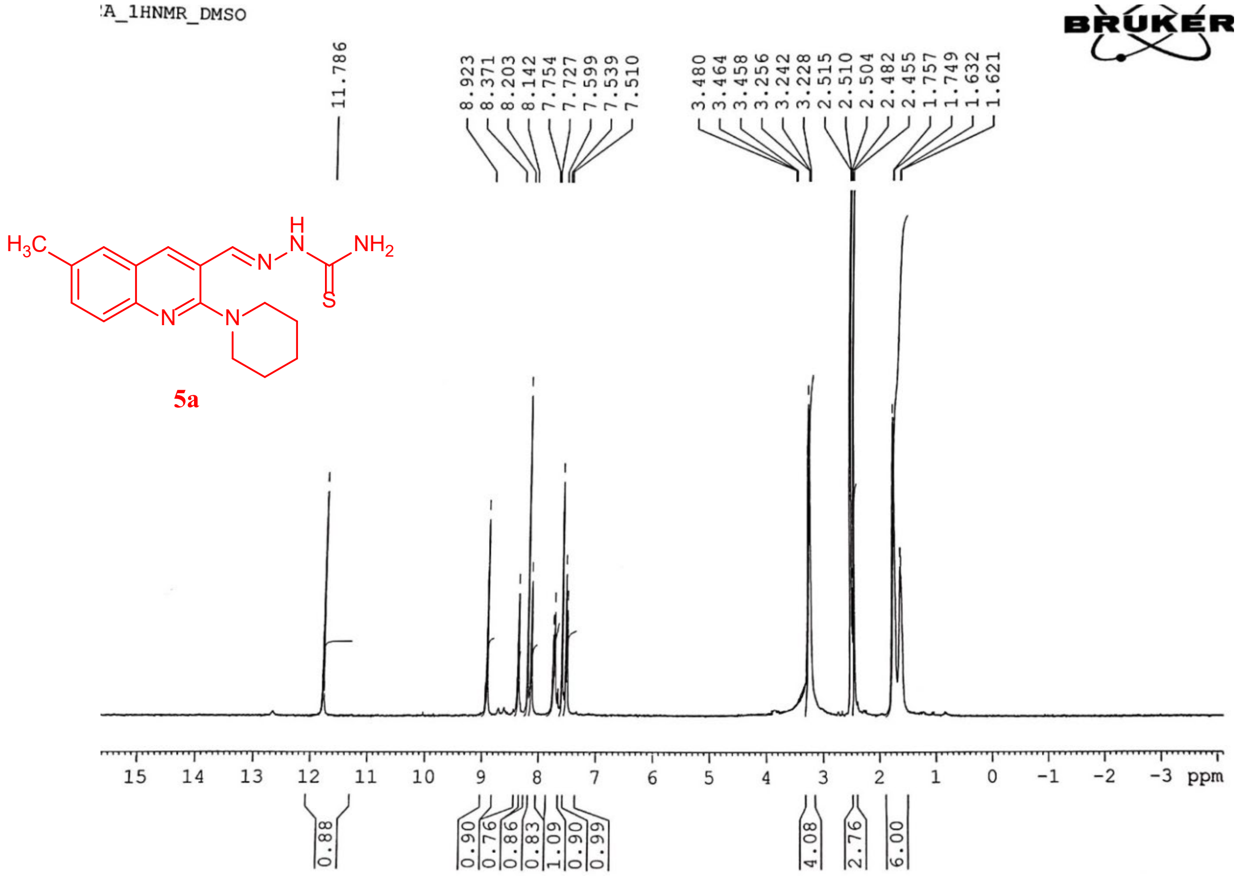

A_1HNMR_DMSO
11.786
8.923
8.371
8.203
8.142
7.754
7.727
7.599
7.539
7.510
3.480
3.464
3.458
3.256
3.242
3.228
2.515
2.510
2.504
2.482
2.455
1.757
1.749
1.632
1.621
BRUKER
H3C
H
N
NH2
N
S
N
N
5a
15 14 13 12 11 10 9 8 7 6 5 4 3 2 1 0 -1 -2 -3 ppm
0.88
0.90
0.76
0.86
0.83
1.09
0.90
0.99
4.08
2.76
6.00

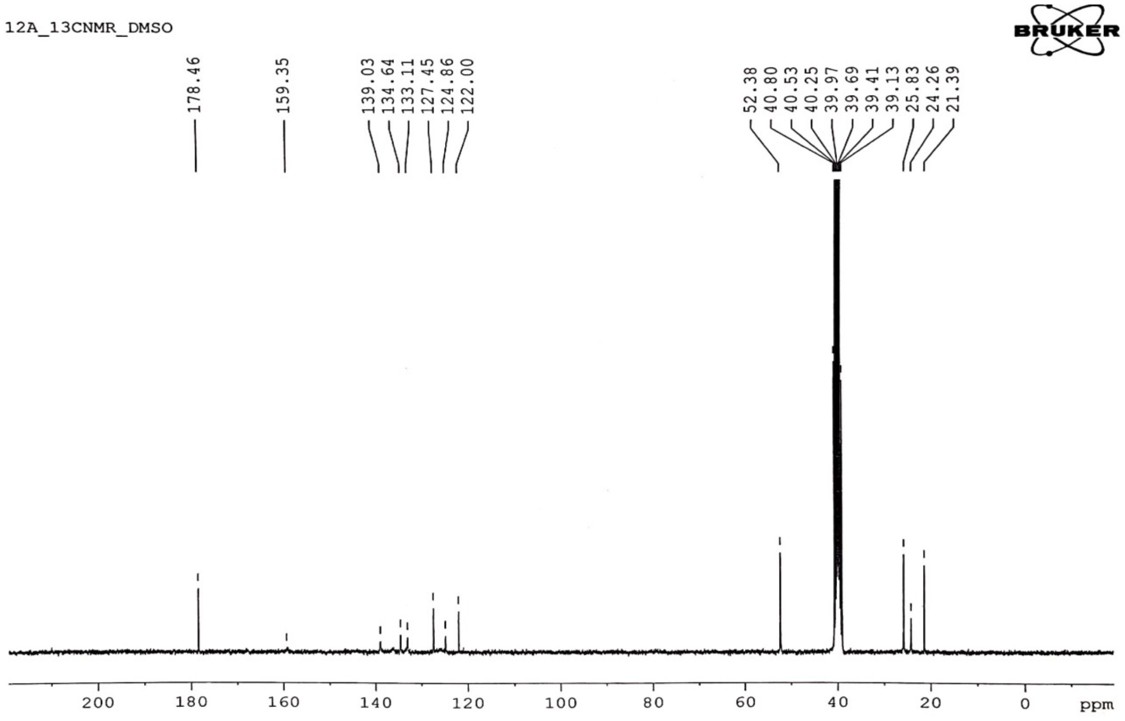

12A_13CNMR_DMSO
178.46
159.35
139.03
134.64
133.11
127.45
124.86
122.00
52.38
40.80
40.53
40.25
39.97
39.69
39.41
39.13
25.83
24.26
21.39
BRUKER
200 180 160 140 120 100 80 60 40 20 0 ppm

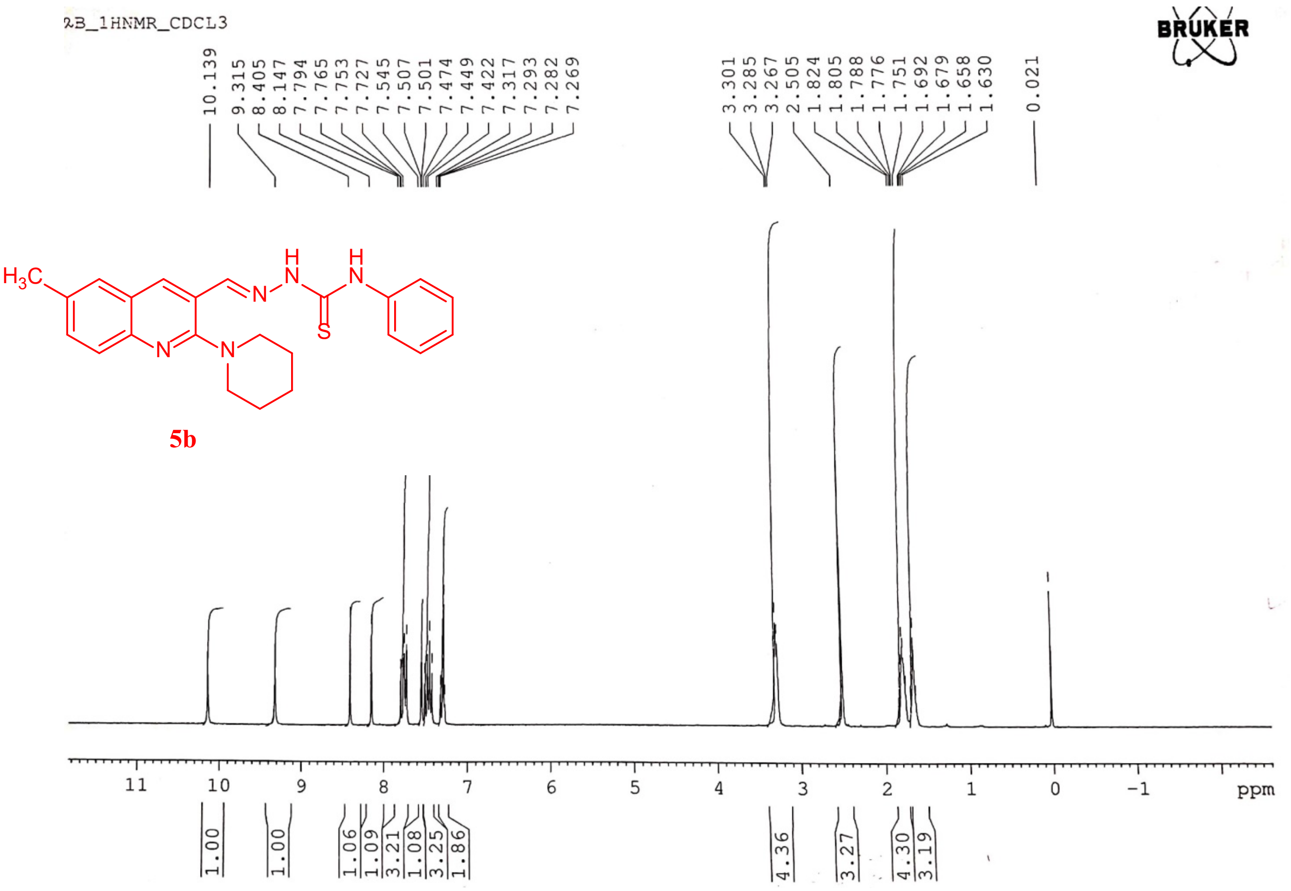
2B_1HNMR_CDCL3
10.139
9.315
8.405
8.147
7.794
7.765
7.753
7.727
7.545
7.507
7.501
7.474
7.449
7.422
7.317
7.293
7.282
7.269
3.301
3.285
3.267
2.505
1.824
1.805
1.788
1.776
1.751
1.692
1.679
1.658
1.630
0.021
BRUKER
11 10 9 8 7 6 5 4 3 2 1 0 −1 ppm
1.00
1.00
1.06
1.09
3.21
1.08
3.25
1.86
4.36
3.27
4.30
3.19
H3C
N
N
N
H
N
H
N
S

5b

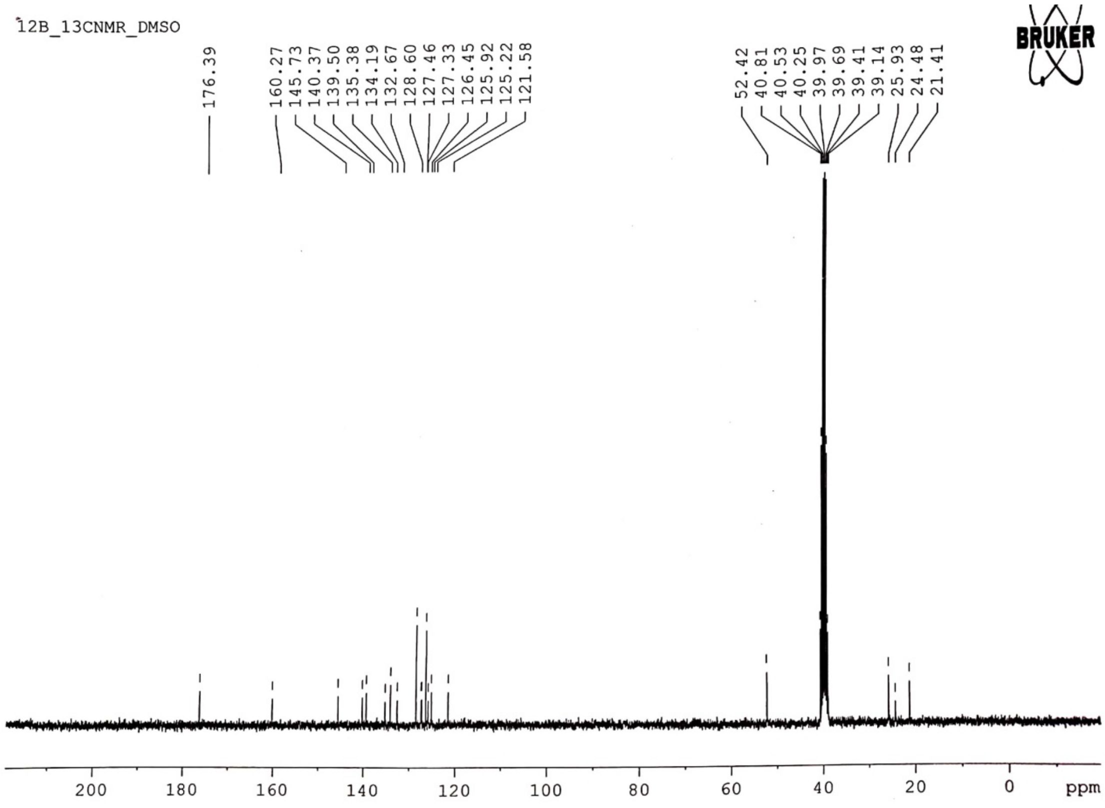
12B_13CNMR_DMSO
176.39
160.27
145.73
140.37
139.50
135.38
134.19
132.67
128.60
127.46
127.33
126.45
125.92
125.22
121.58
52.42
40.81
40.53
40.25
39.97
39.69
39.41
39.14
25.93
24.48
21.41
BRUKER
200 180 160 140 120 100 80 60 40 20 0 ppm

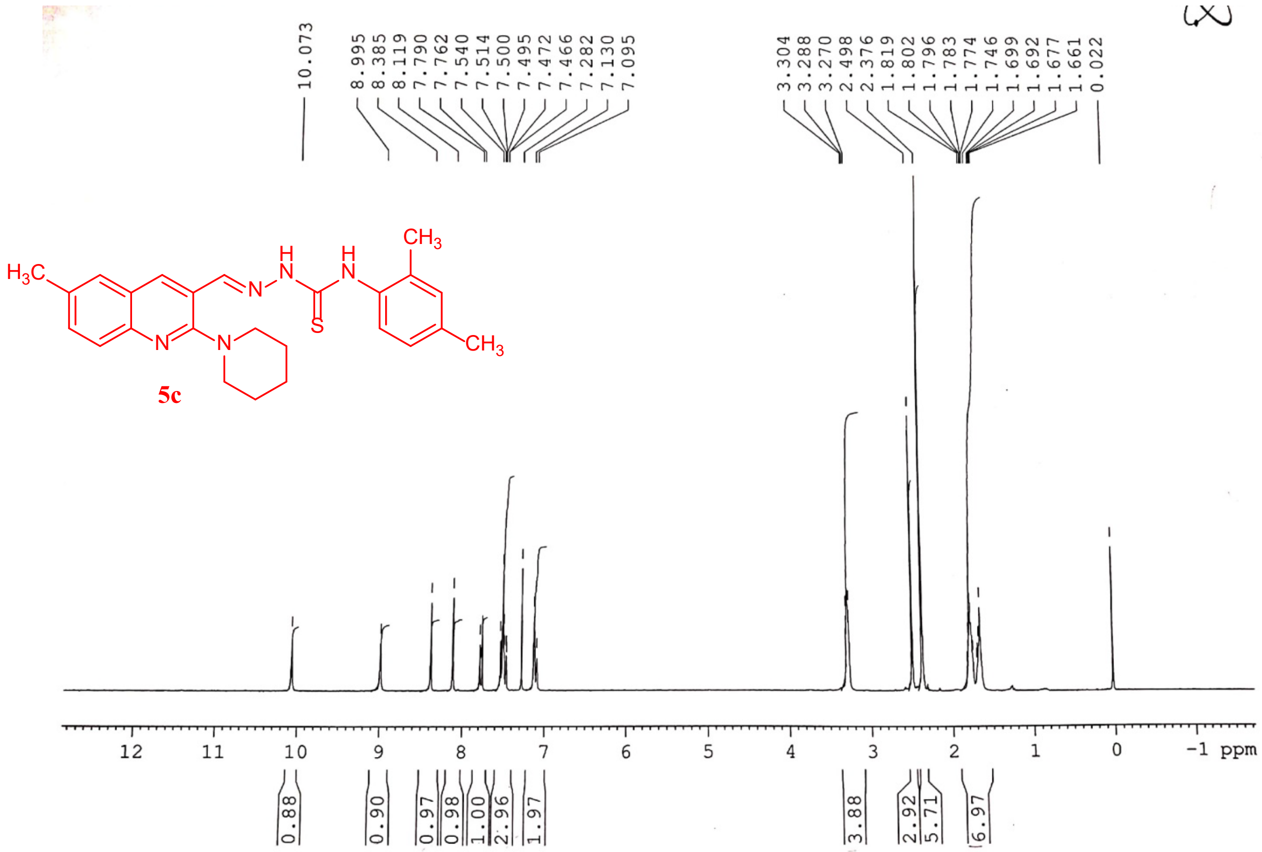
10.073
8.995
8.385
8.119
7.790
7.762
7.540
7.514
7.500
7.495
7.472
7.466
7.282
7.130
7.095
3.304
3.288
3.270
2.498
2.376
1.819
1.802
1.796
1.783
1.774
1.746
1.699
1.692
1.677
1.661
0.022
H3C
CH3
CH3
N
N
N
H
N
H
S
5c
12 11 10 9 8 7 6 5 4 3 2 1 0 -1 ppm
0.88
0.90
0.97
0.98
1.00
2.96
1.97
3.88
2.92
5.71
6.97

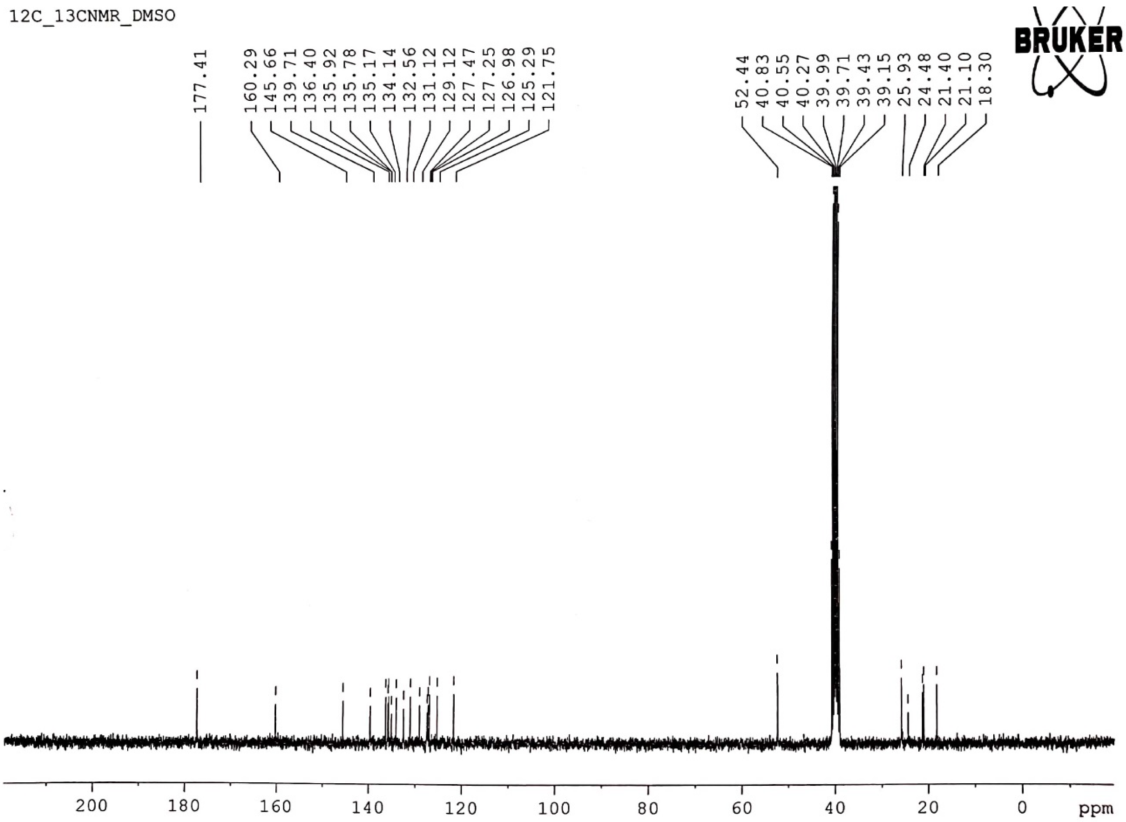
12C_13CNMR_DMSO
177.41
160.29
145.66
139.71
136.40
135.92
135.78
135.17
134.14
132.56
131.12
129.12
127.47
127.25
126.98
125.29
121.75
52.44
40.83
40.55
40.27
39.99
39.71
39.43
39.15
25.93
24.48
21.40
21.10
18.30
BRUKER
200 180 160 140 120 100 80 60 40 20 0 ppm

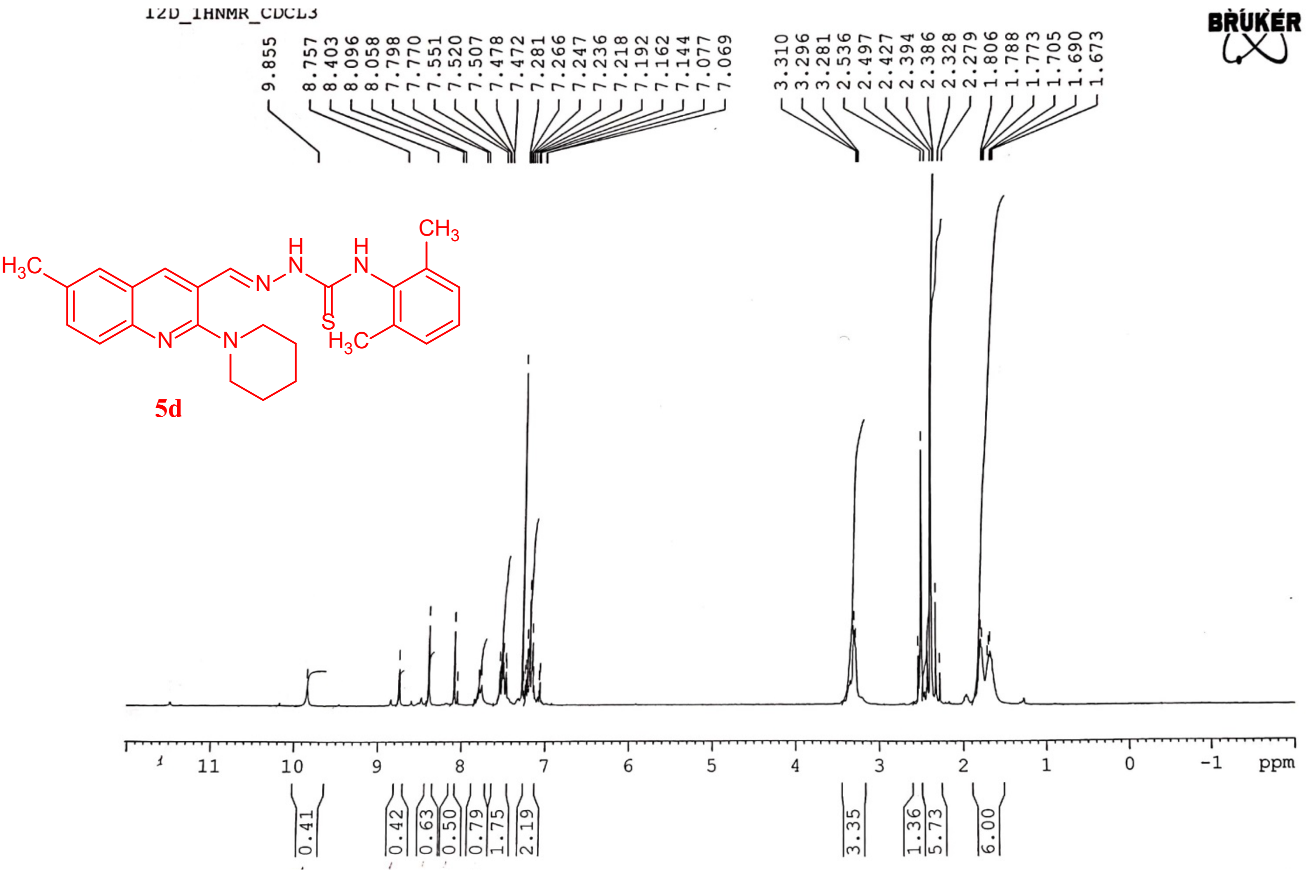
12D_1HNMR_CDCL3
9.855 8.757 8.403 8.096 8.058 7.798 7.770 7.551 7.520 7.507 7.478 7.472 7.281 7.266 7.247 7.236 7.218 7.192 7.162 7.144 7.077 7.069
3.310 3.296 3.281 2.536 2.497 2.427 2.394 2.386 2.328 2.279 1.806 1.788 1.773 1.705 1.690 1.673
BRUKER
H3C
H
N
H
N
CH3
N
S
H3C
N
N
11 10 9 8 7 6 5 4 3 2 1 0 -1 ppm
0.41 0.42 0.63 0.50 0.79 1.75 2.19 3.35 1.36 5.73 6.00

5d

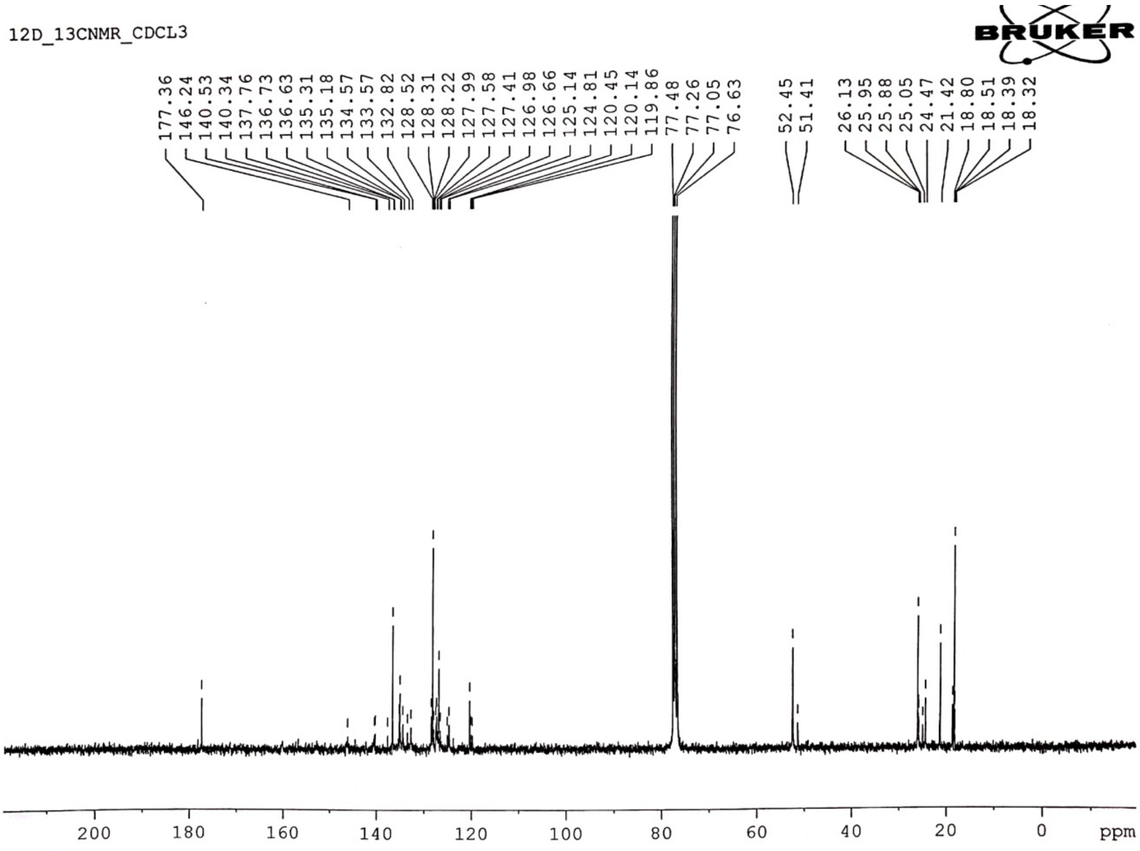
12D_13CNMR_CDCL3
177.36 146.24 140.53 140.34 137.76 136.73 136.63 135.31 135.18 134.57 133.57 132.82 128.52 128.31 128.22 127.99 127.58 127.41 126.98 126.66 125.14 124.81 120.45 120.14 119.86 77.48 77.26 77.05 76.63
52.45 51.41
26.13 25.95 25.88 25.05 24.47 21.42 18.80 18.51 18.39 18.32
BRUKER
200 180 160 140 120 100 80 60 40 20 0 ppm

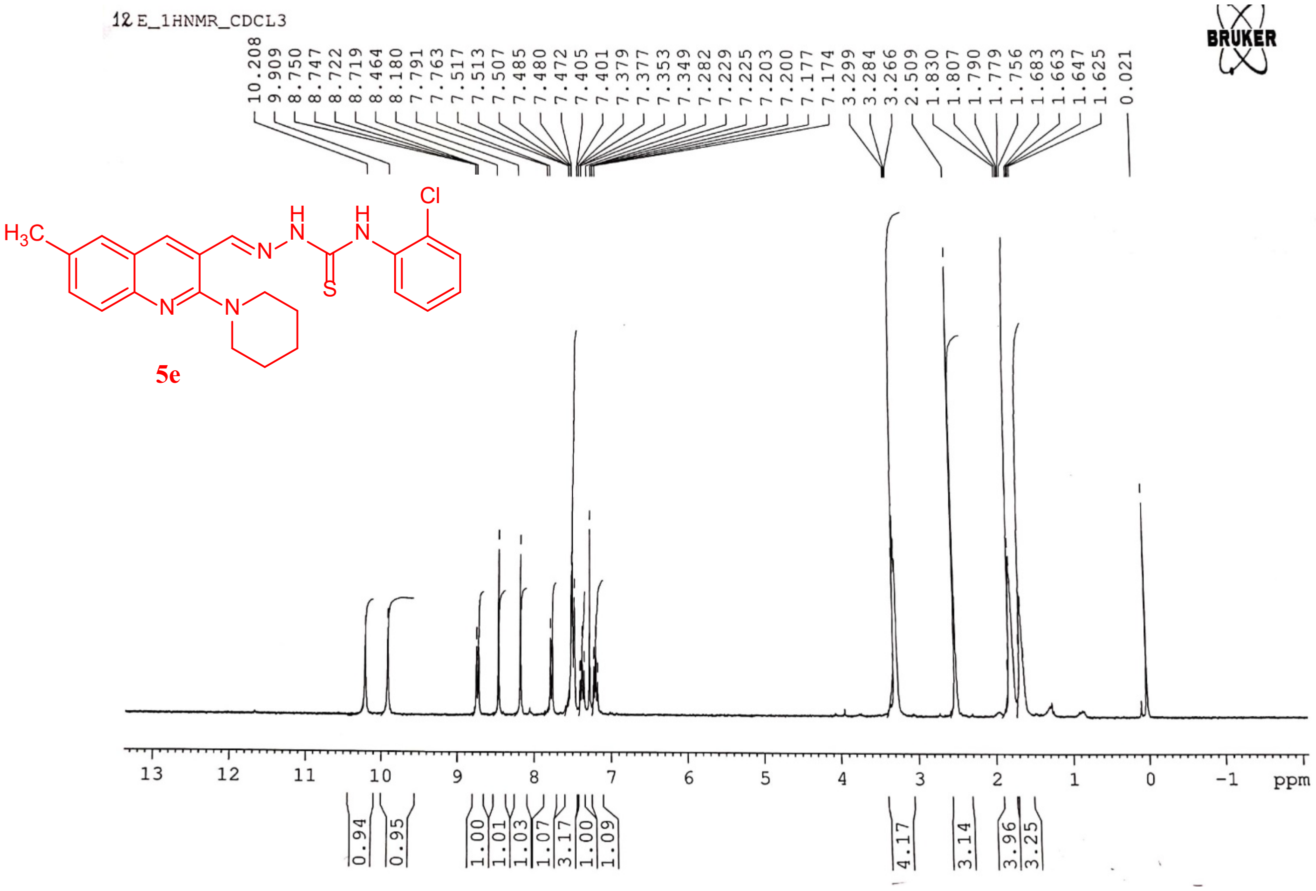
12E_1HNMR_CDCL3
10.208
9.909
8.750
8.747
8.722
8.719
8.464
8.180
7.791
7.763
7.517
7.513
7.507
7.485
7.480
7.472
7.405
7.401
7.379
7.377
7.353
7.349
7.282
7.229
7.225
7.203
7.200
7.177
7.174
3.299
3.284
3.266
2.509
1.830
1.807
1.790
1.779
1.756
1.683
1.663
1.647
1.625
0.021
BRUKER
H3C
N
N
H
N
H
N
S
Cl
N
5e
ppm
0.94
0.95
1.00
1.01
1.03
1.07
3.17
1.00
1.09
4.17
3.14
3.96
3.25

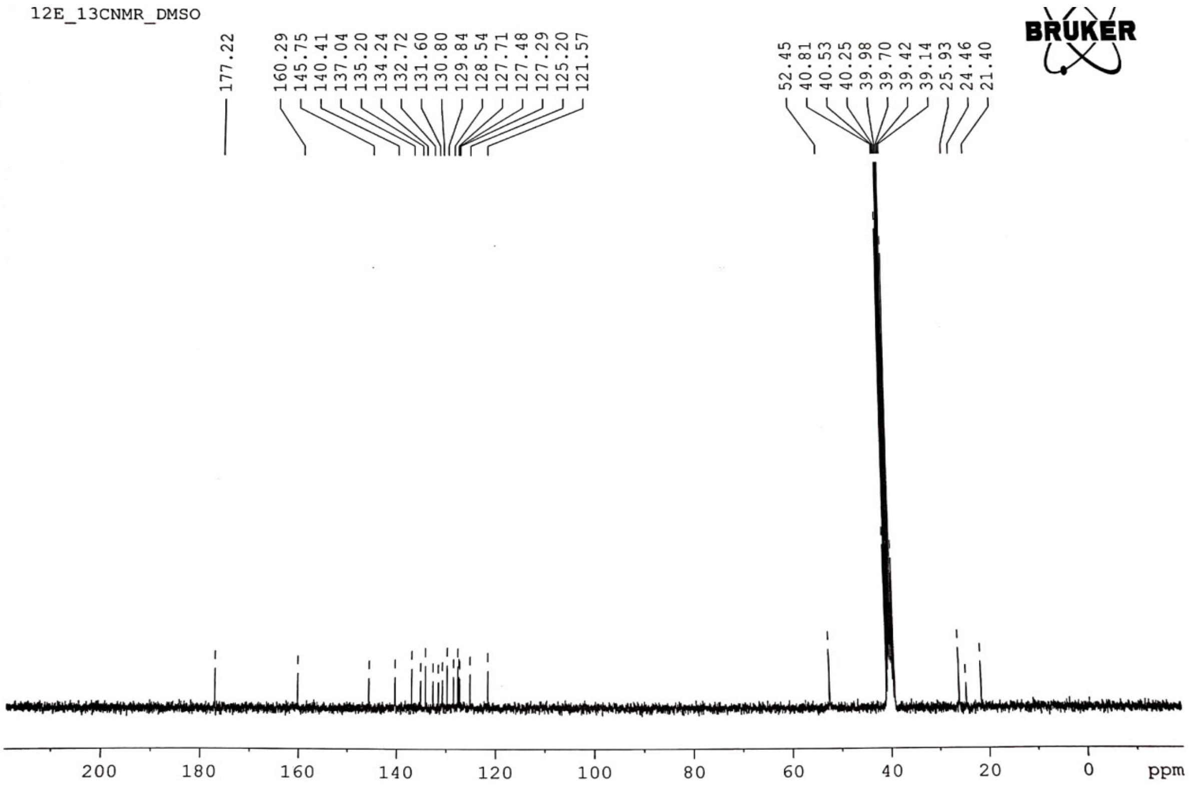
12E_13CNMR_DMSO
177.22
160.29
145.75
140.41
137.04
135.20
134.24
132.72
131.60
130.80
129.84
128.54
127.71
127.48
127.29
125.20
121.57
52.45
40.81
40.53
40.25
39.98
39.70
39.42
39.14
25.93
24.46
21.40
BRUKER
ppm

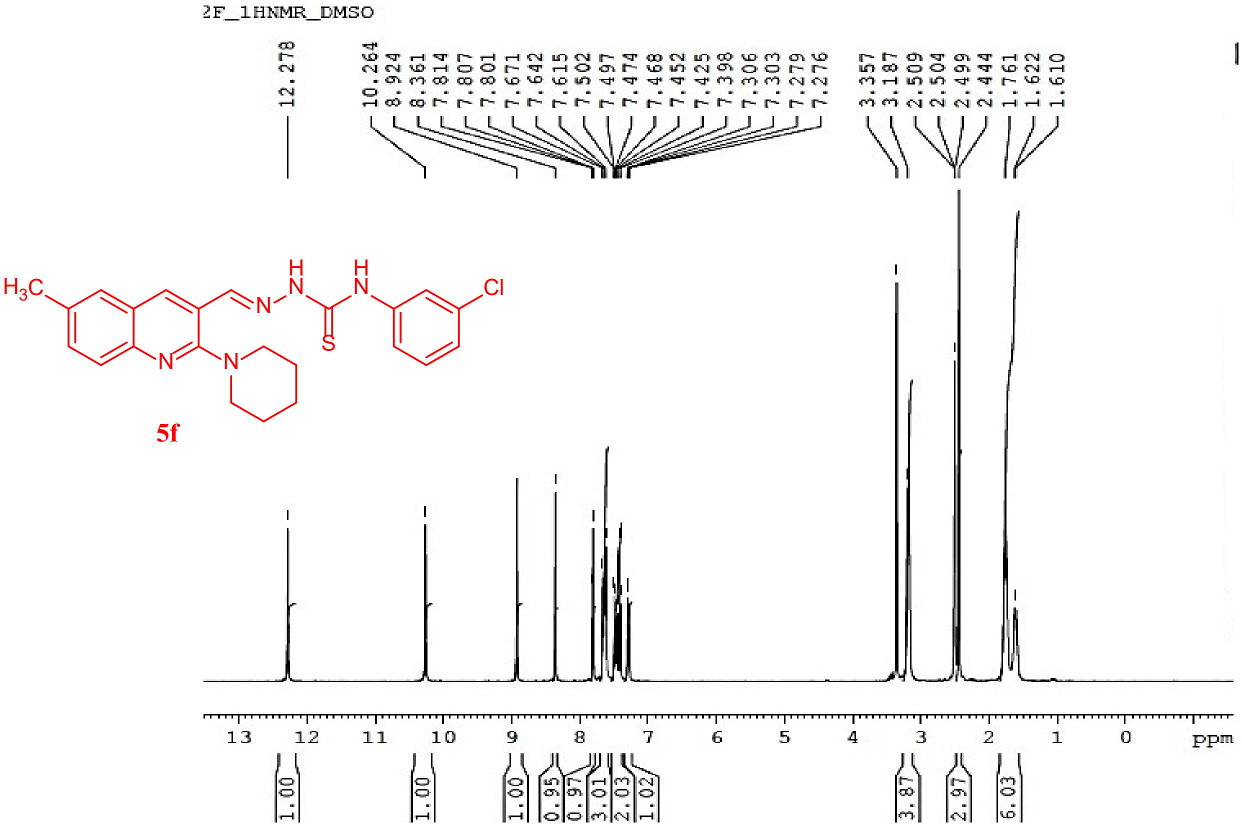
2F_1HNMR_DMSO
12.278
10.264
8.924
8.361
7.814
7.807
7.801
7.671
7.642
7.615
7.502
7.497
7.474
7.468
7.452
7.425
7.398
7.306
7.303
7.279
7.276
3.357
3.187
2.509
2.504
2.499
2.444
1.761
1.622
1.610
H3C
H
N
H
N
N
S
Cl
N
N
5f
13 12 11 10 9 8 7 6 5 4 3 2 1 0 ppm
1.00
1.00
1.00
0.95
0.97
3.01
2.03
1.02
3.87
2.97
6.03

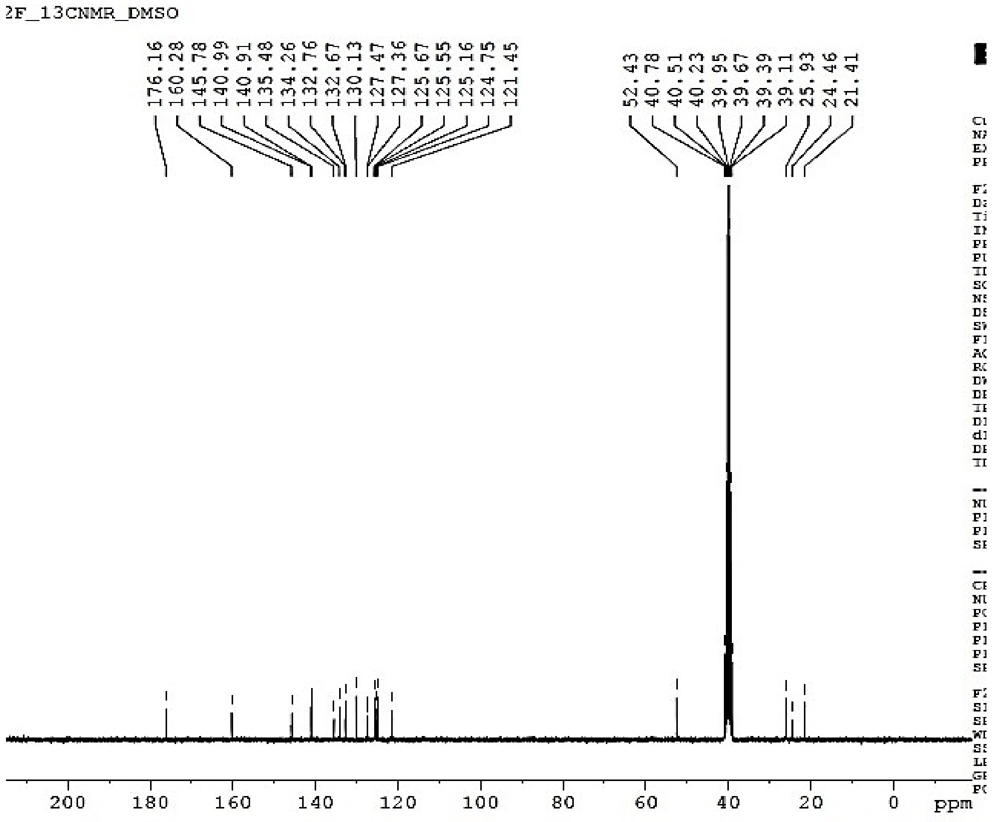
2F_13CNMR_DMSO
176.16
160.28
145.78
140.99
140.91
135.48
134.26
132.76
132.67
130.13
127.47
127.36
125.67
125.55
125.16
124.75
121.45
52.43
40.78
40.51
40.23
39.95
39.67
39.39
39.11
25.93
24.46
21.41
200 180 160 140 120 100 80 60 40 20 0 ppm

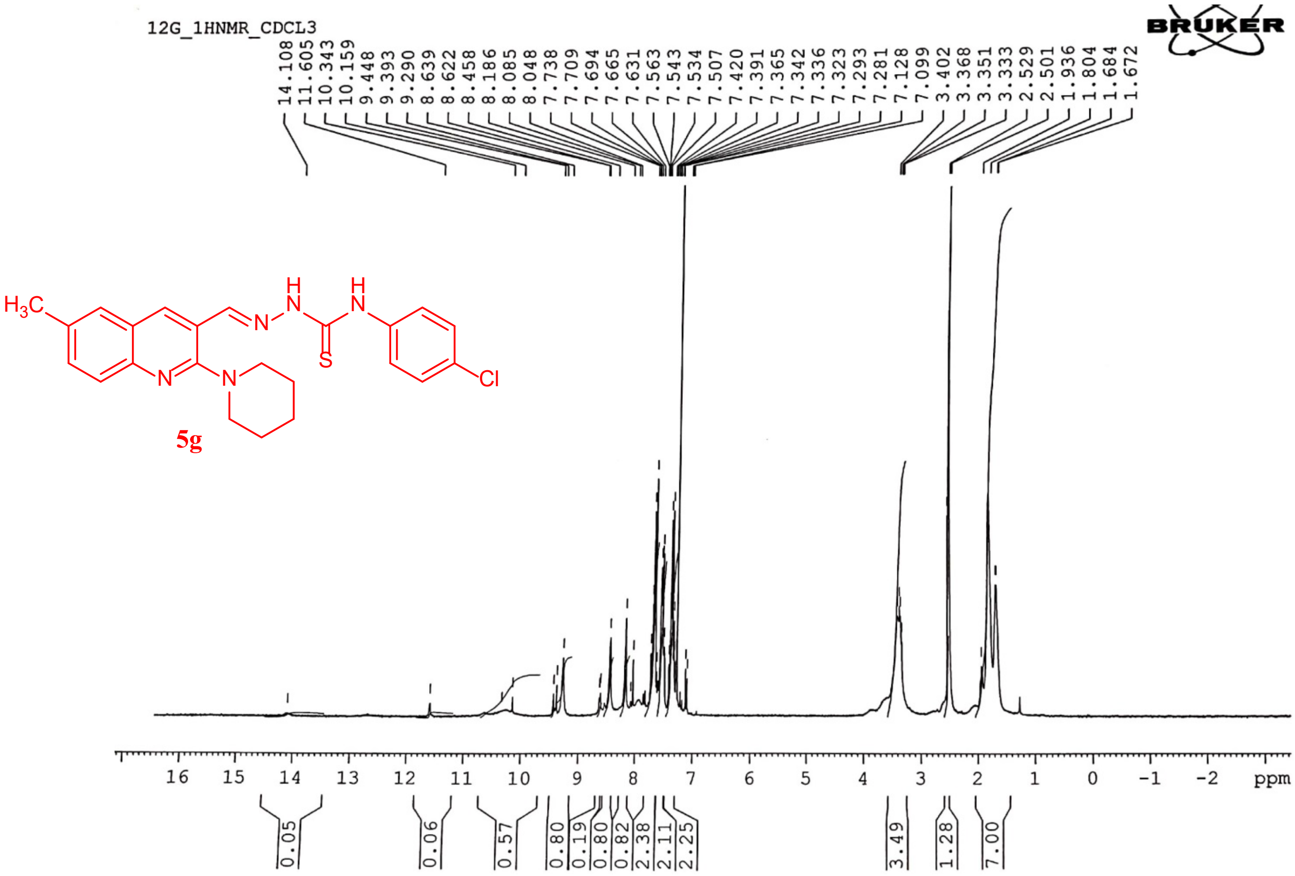

12G_1HNMR_CDCL3
14.108
11.605
10.343
10.159
9.448
9.393
9.290
8.639
8.622
8.458
8.186
8.085
8.048
7.738
7.709
7.694
7.665
7.631
7.563
7.543
7.534
7.507
7.420
7.391
7.365
7.342
7.336
7.323
7.293
7.281
7.128
7.099
3.402
3.368
3.351
3.333
2.529
2.501
1.936
1.804
1.684
1.672
ppm
0.05
0.06
0.57
0.80
0.19
0.80
0.82
2.38
2.11
2.25
3.49
1.28
7.00
H3C
H
N
N
H
N
S
N
N
Cl

5g

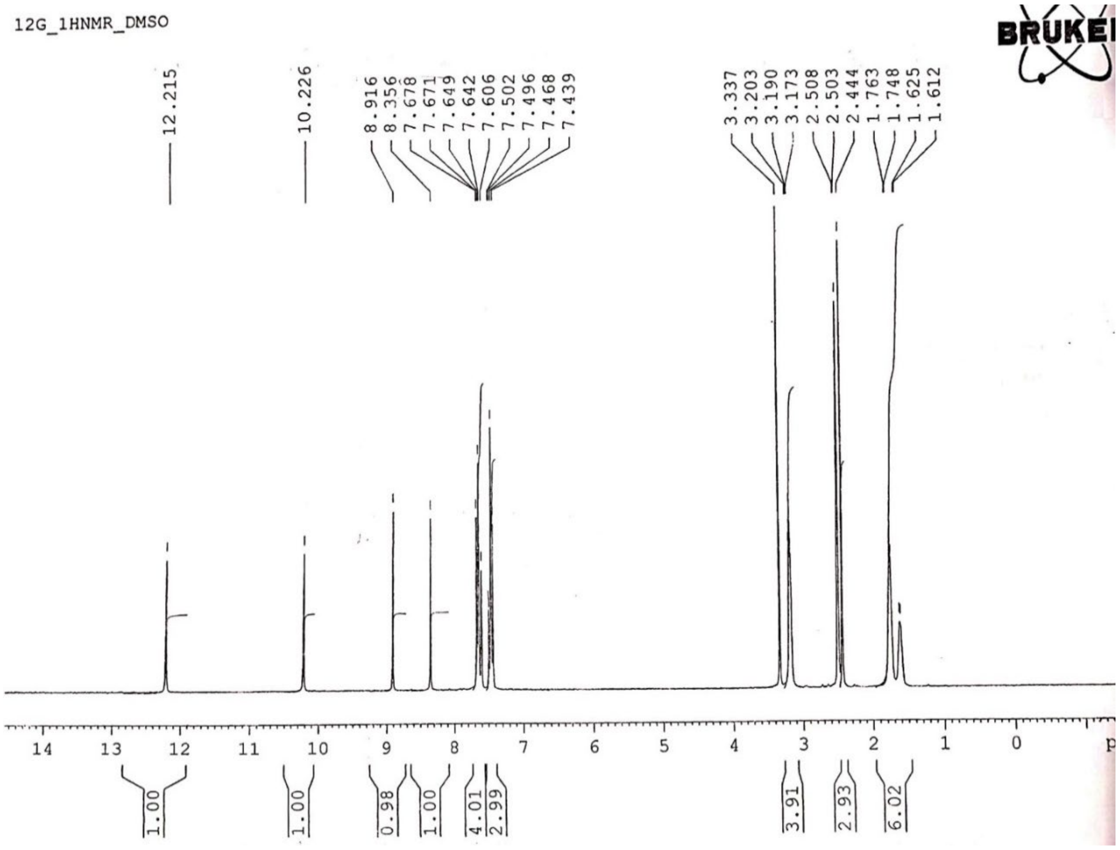

12G_1HNMR_DMSO
12.215
10.226
8.916
8.356
7.678
7.671
7.649
7.642
7.606
7.502
7.496
7.468
7.439
3.337
3.203
3.190
3.173
2.508
2.503
2.444
1.763
1.748
1.625
1.612
1.00
1.00
0.98
1.00
4.01
2.99
3.91
2.93
6.02

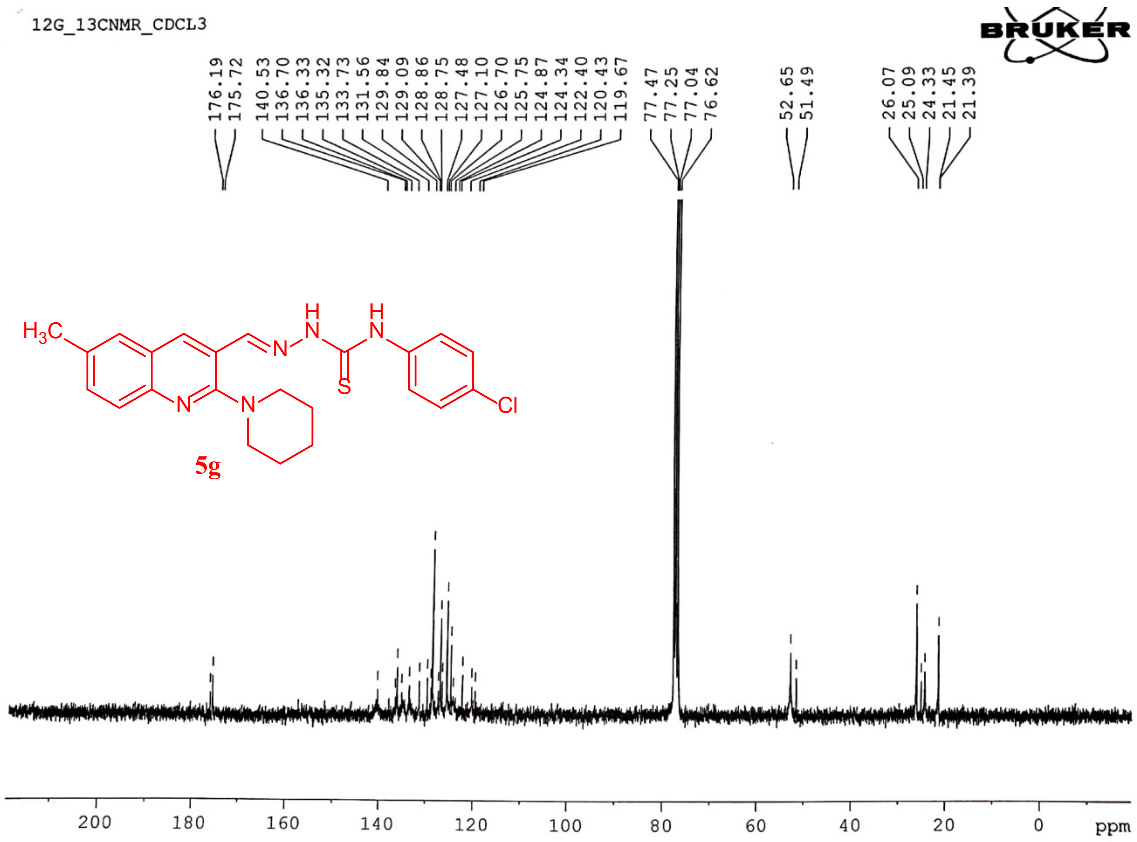
12G_13CNMR_CDCL3
BRUKER
176.19
175.72
140.53
136.70
136.33
135.32
133.73
131.56
129.84
129.09
128.86
128.75
127.48
127.10
126.70
125.75
124.87
124.34
122.40
120.43
119.67
77.47
77.25
77.04
76.62
52.65
51.49
26.07
25.09
24.33
21.45
21.39
H3C
H
N
N
H
N
S
N
N
Cl
5g
200
180
160
140
120
100
80
60
40
20
0
ppm

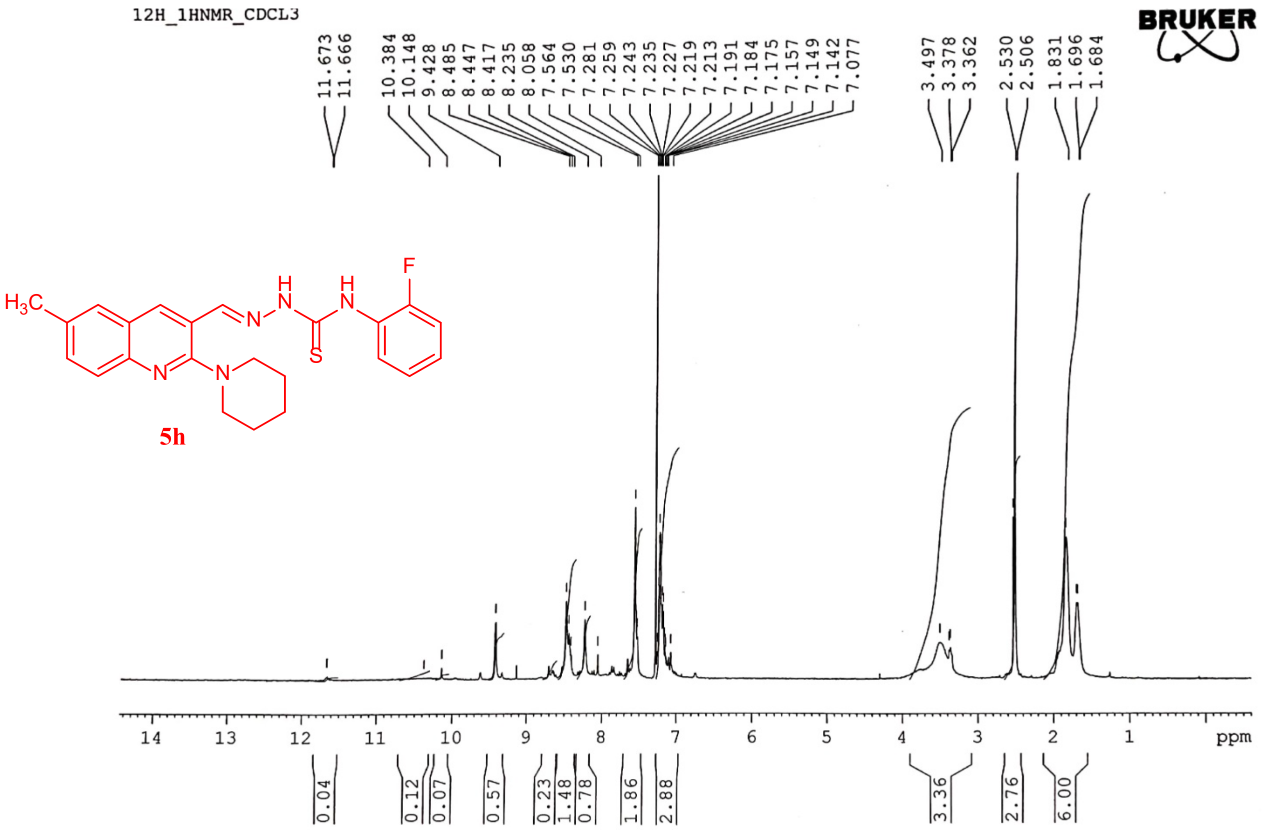
12H_1HNMR_CDCL3
BRUKER
11.673
11.666
10.384
10.148
9.428
8.485
8.447
8.417
8.235
8.058
7.564
7.530
7.281
7.259
7.243
7.235
7.227
7.219
7.213
7.191
7.184
7.175
7.157
7.149
7.142
7.077
3.497
3.378
3.362
2.530
2.506
1.831
1.696
1.684
H_3C
N
N
N
H
N
H
N
F
S
5h
14 13 12 11 10 9 8 7 6 5 4 3 2 1 ppm
0.04
0.12
0.07
0.57
0.23
1.48
0.78
1.86
2.88
3.36
2.76
6.00

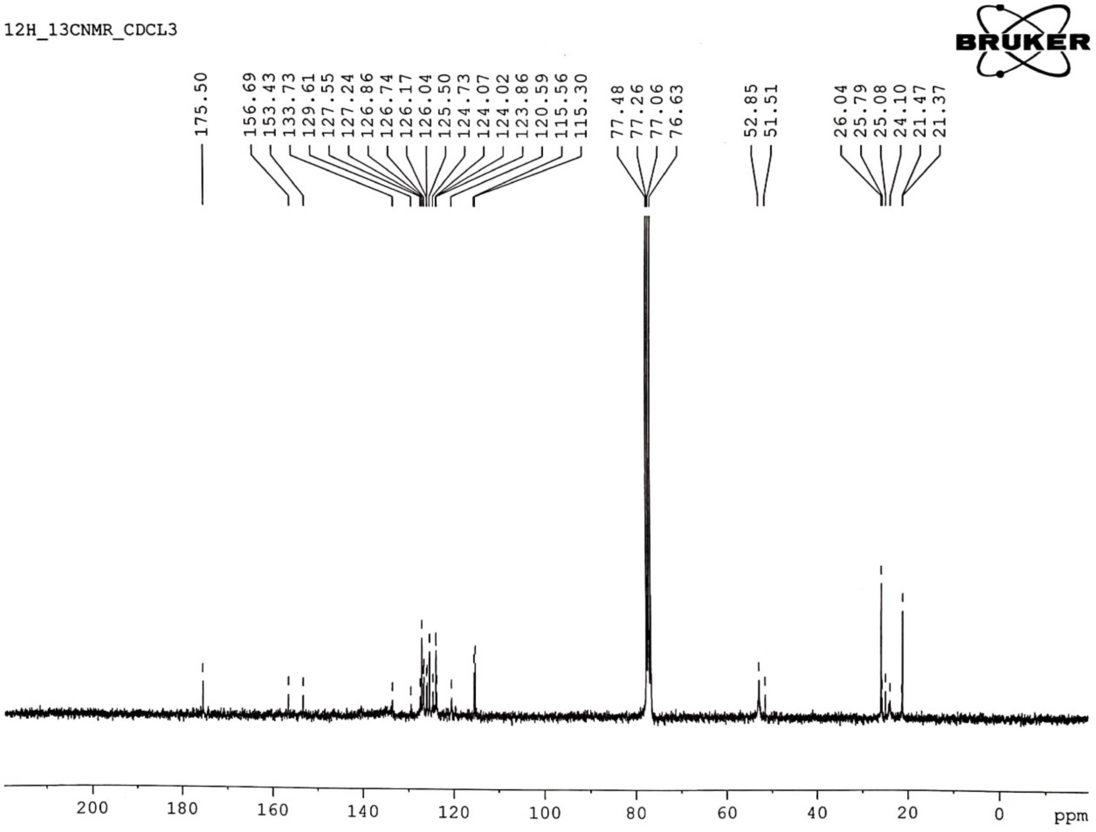
12H_13CNMR_CDCL3
BRUKER
175.50
156.69
153.43
133.73
129.61
127.55
127.24
126.86
126.74
126.17
126.04
125.50
124.73
124.07
124.02
123.86
120.59
115.56
115.30
77.48
77.26
77.06
76.63
52.85
51.51
26.04
25.79
25.08
24.10
21.47
21.37
200 180 160 140 120 100 80 60 40 20 0 ppm

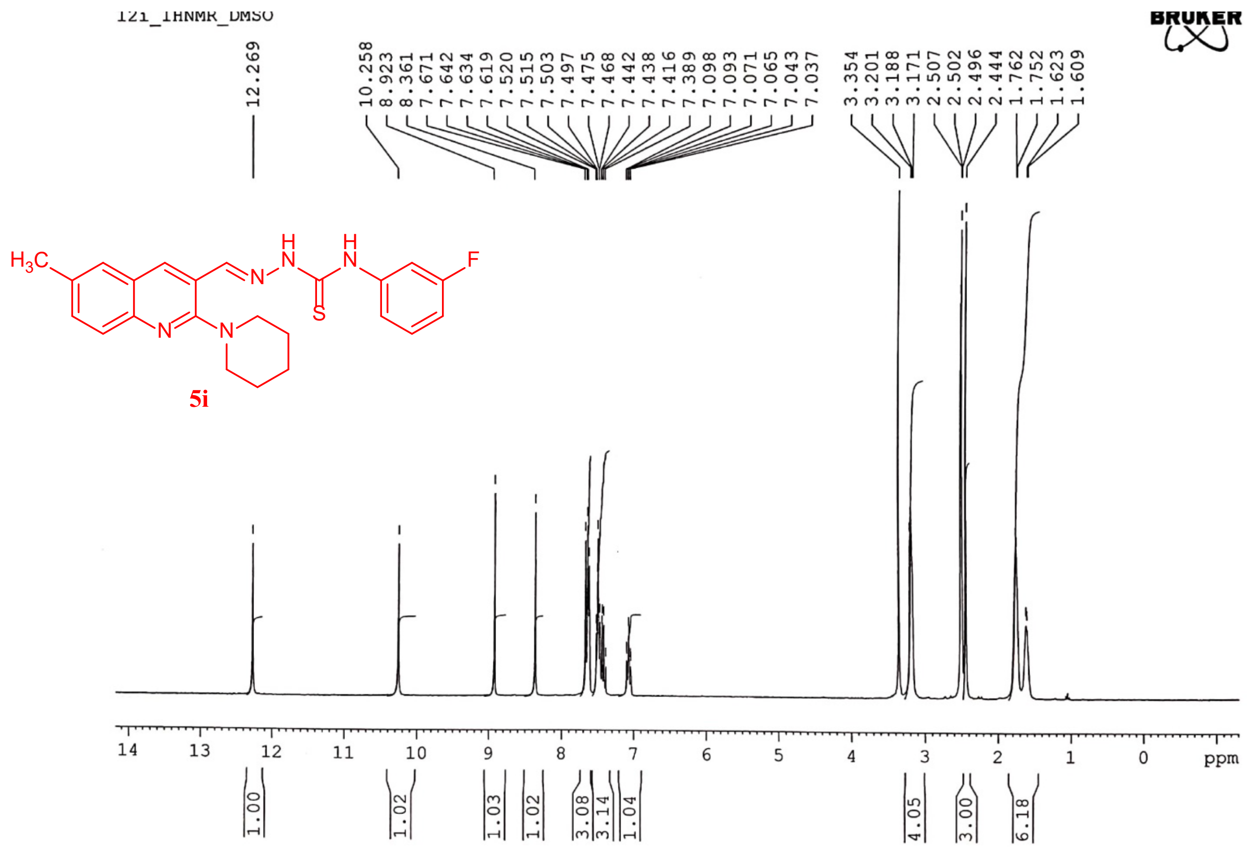
12i_1HNMR_DMSO
BRUKER
12.269
10.258 8.923 8.361 7.671 7.642 7.634 7.619 7.520 7.515 7.503 7.497 7.475 7.468 7.442 7.438 7.416 7.389 7.098 7.093 7.071 7.065 7.043 7.037
3.354 3.201 3.188 3.171 2.507 2.502 2.496 2.444 1.762 1.752 1.623 1.609
H_3C
H
N
S
F
5i
14 13 12 11 10 9 8 7 6 5 4 3 2 1 0 ppm
1.00 1.02 1.03 1.02 3.08 3.14 1.04 4.05 3.00 6.18

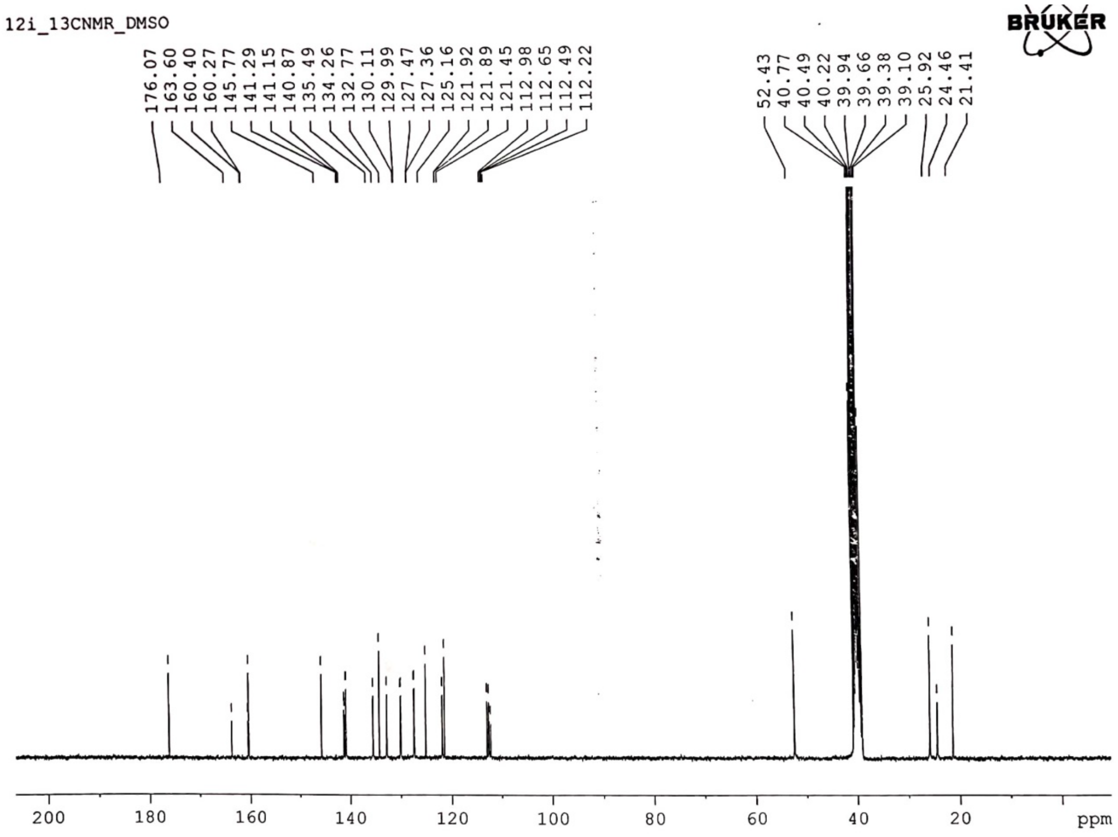
12i_13CNMR_DMSO
BRUKER
176.07 163.60 160.40 160.27 145.77 141.29 141.15 140.87 135.49 134.26 132.77 130.11 129.99 127.47 127.36 125.16 121.92 121.89 121.45 112.98 112.65 112.49 112.22
52.43 40.77 40.49 40.22 39.94 39.66 39.38 39.10 25.92 24.46 21.41
200 180 160 140 120 100 80 60 40 20 ppm

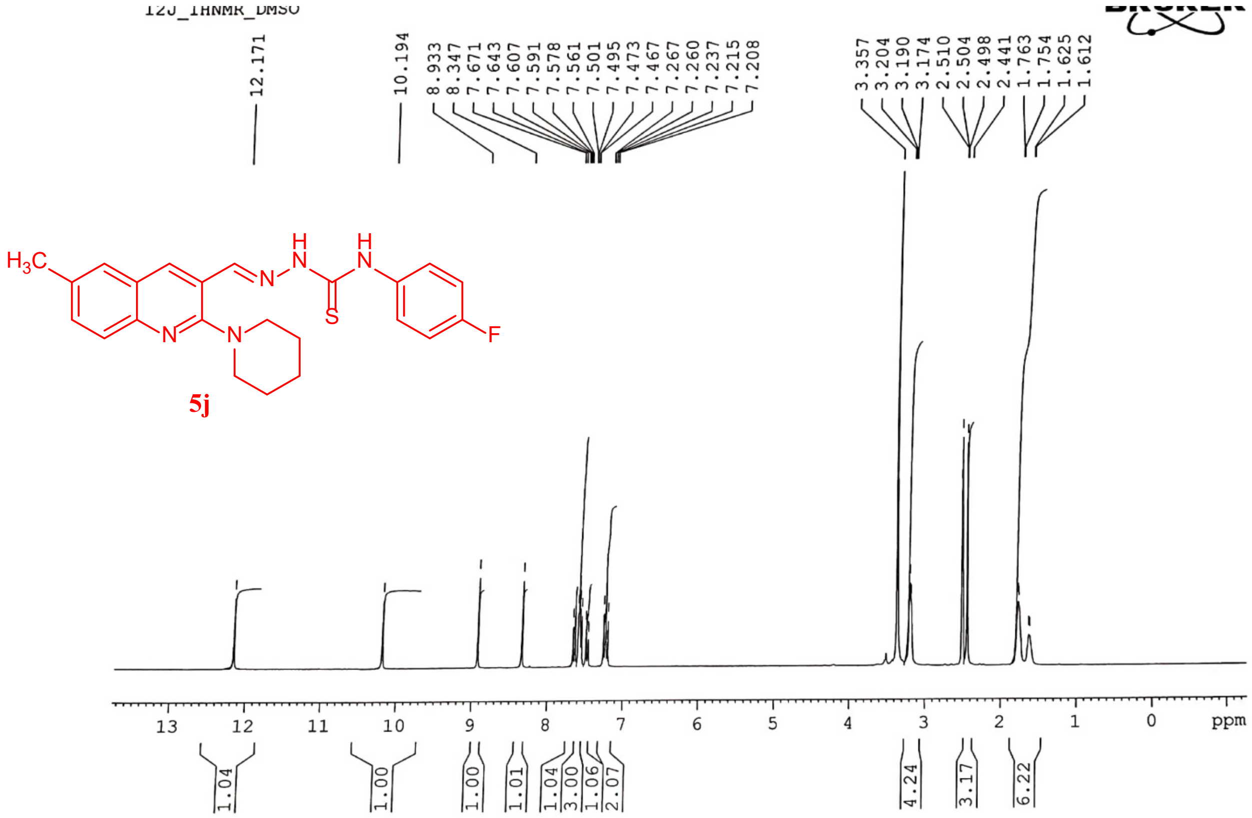
12J_1HNMR_DMSO
12.171
10.194
8.933
8.347
7.671
7.643
7.607
7.591
7.578
7.561
7.501
7.495
7.473
7.467
7.267
7.260
7.237
7.215
7.208
3.357
3.204
3.190
3.174
2.510
2.504
2.498
2.441
1.763
1.754
1.625
1.612
H3C
N
N
H
N
H
N
S
F
N
5j
13
12
11
10
9
8
7
6
5
4
3
2
1
0
ppm
1.04
1.00
1.00
1.01
1.04
3.00
1.06
2.07
4.24
3.17
6.22

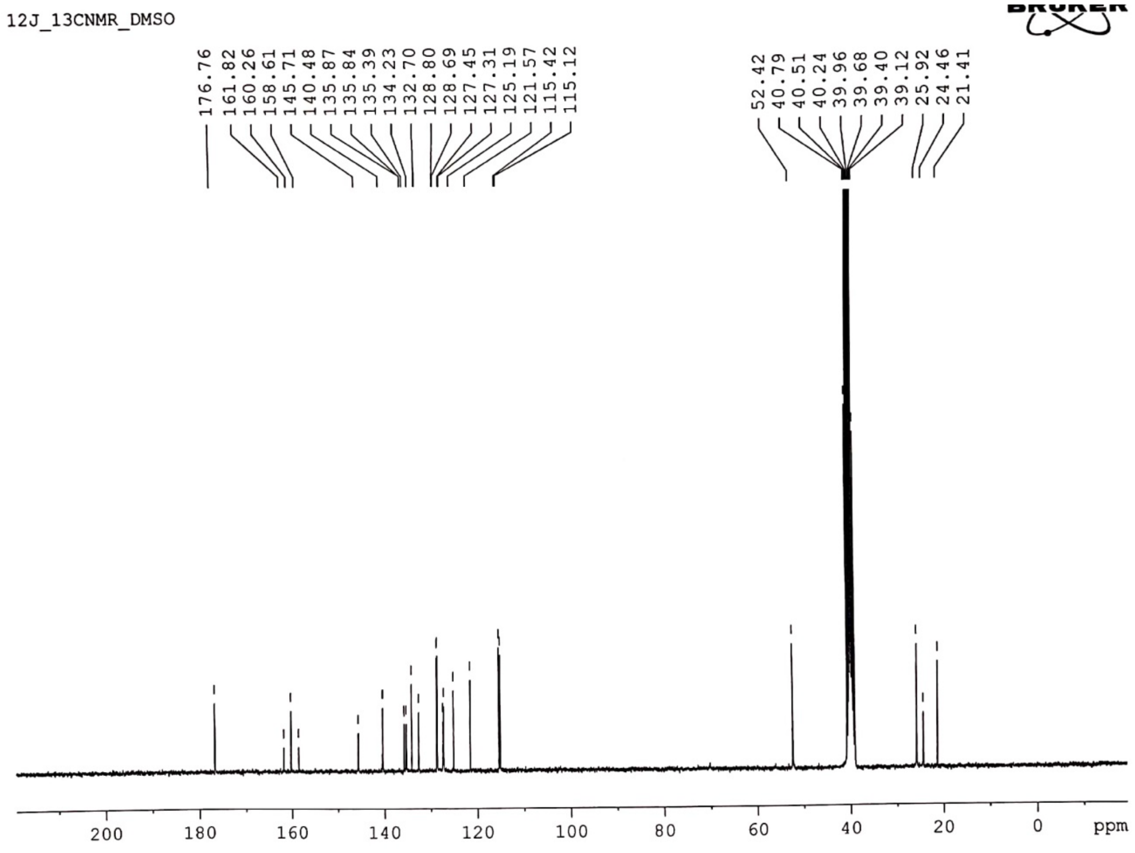
12J_13CNMR_DMSO
176.76
161.82
160.26
158.61
145.71
140.48
135.87
135.84
135.39
134.23
132.70
128.80
128.69
127.45
127.31
125.19
121.57
115.42
115.12
52.42
40.79
40.51
40.24
39.96
39.68
39.40
39.12
25.92
24.46
21.41
200
180
160
140
120
100
80
60
40
20
0
ppm

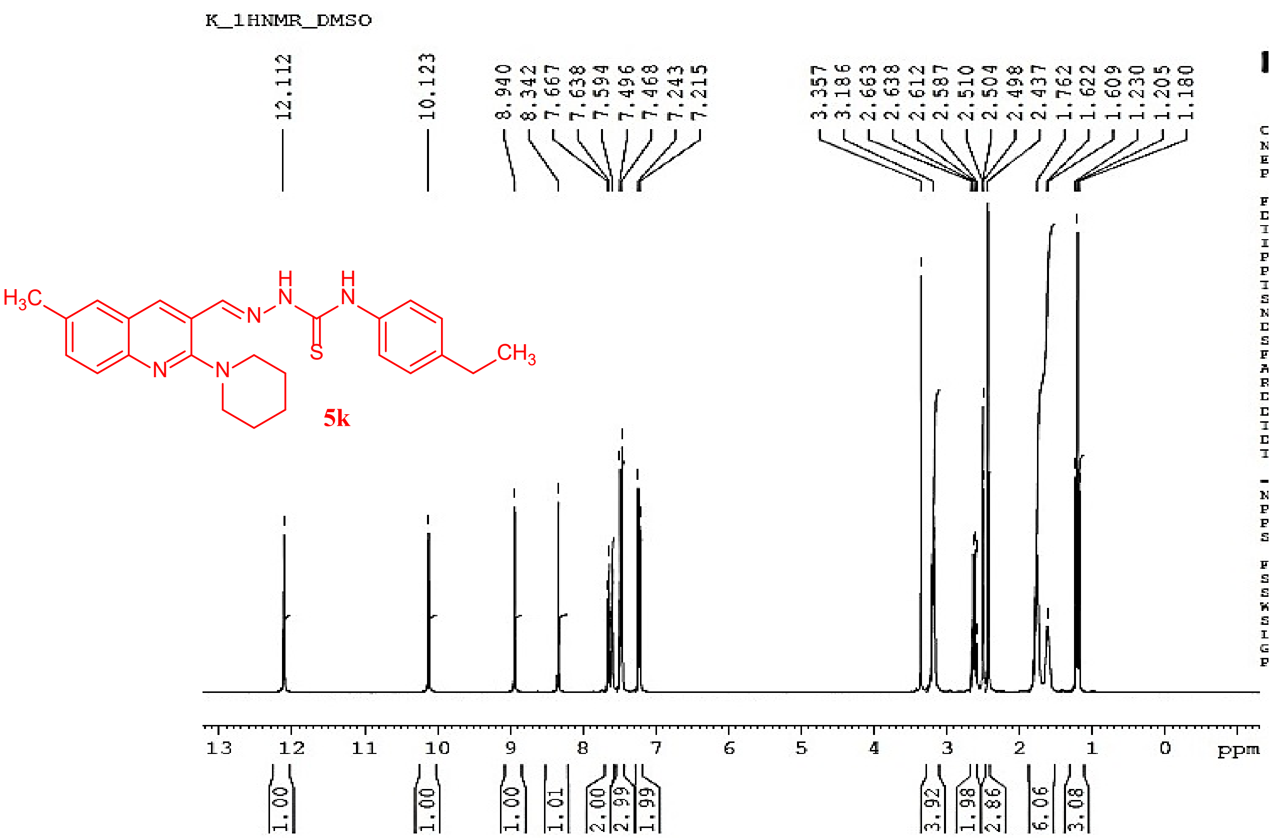
K_1HNMR_DMSO
12.112
10.123
8.940
8.342
7.667
7.638
7.594
7.496
7.468
7.243
7.215
3.357
3.186
2.663
2.638
2.612
2.587
2.510
2.504
2.498
2.437
1.762
1.622
1.609
1.230
1.205
1.180
H_3C
H
N
N
H
N
S
N
N
CH_3
5k
13 12 11 10 9 8 7 6 5 4 3 2 1 0 ppm
1.00
1.00
1.00
1.01
2.00
2.99
1.99
3.92
1.98
2.86
6.06
3.08

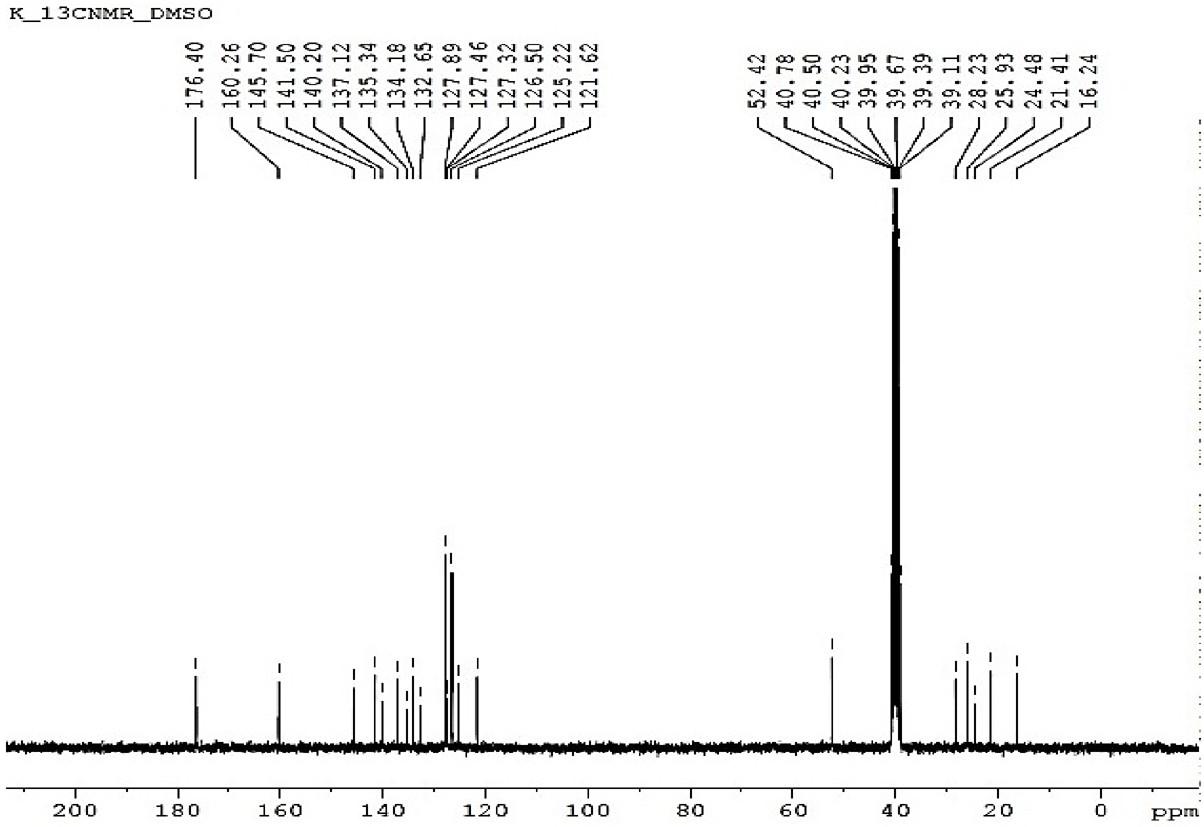
K_13CNMR_DMSO
176.40
160.26
145.70
141.50
140.20
137.12
135.34
134.18
132.65
127.89
127.46
127.32
126.50
125.22
121.62
52.42
40.78
40.50
40.23
39.95
39.67
39.39
39.11
28.23
25.93
24.48
21.41
16.24
200 180 160 140 120 100 80 60 40 20 0 ppm

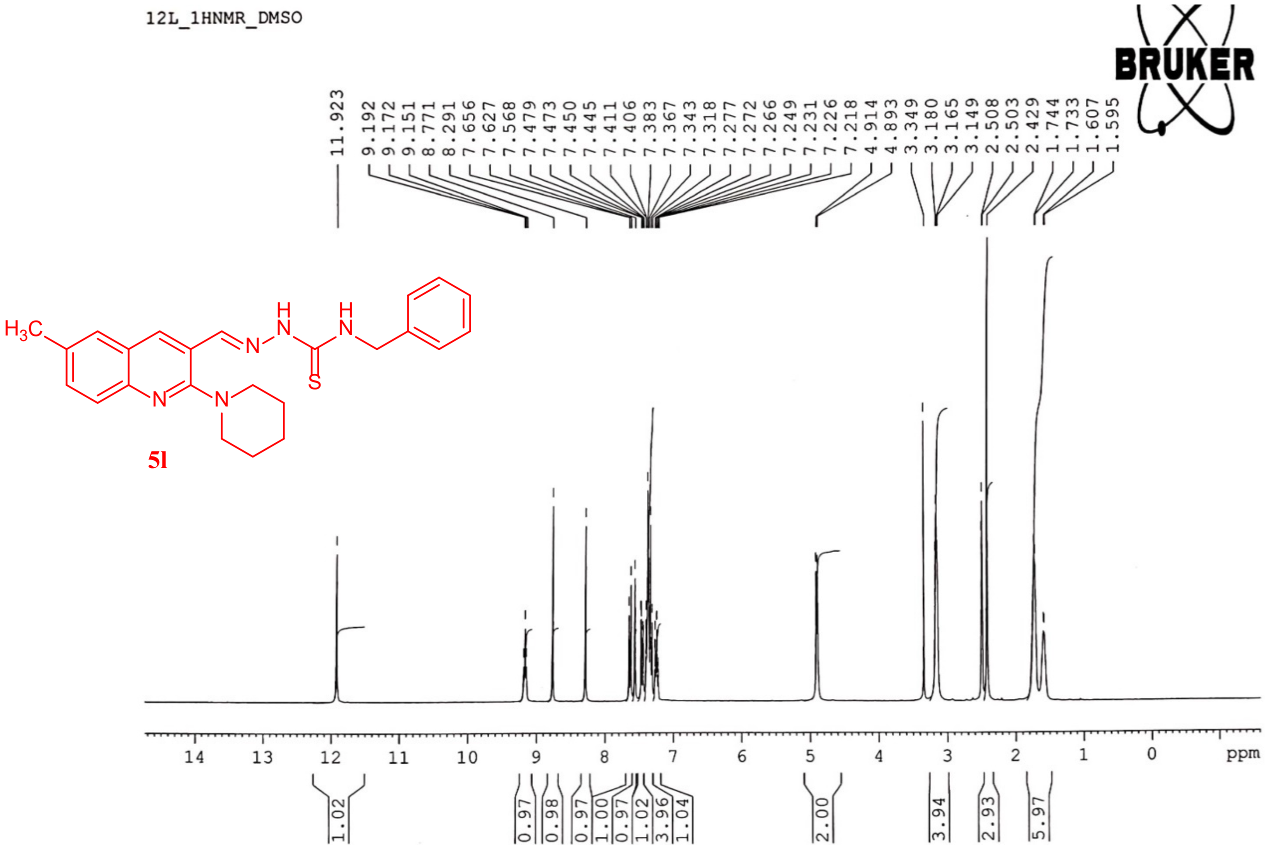
12L_1HNMR_DMSO
BRUKER
11.923
9.192 9.172 9.151 8.771 8.291 7.656 7.627 7.568 7.479 7.473 7.450 7.445 7.411 7.406 7.383 7.367 7.343 7.318 7.277 7.272 7.266 7.249 7.231 7.226 7.218 4.914 4.893 3.349 3.180 3.165 3.149 2.508 2.503 2.429 1.744 1.733 1.607 1.595
14 13 12 11 10 9 8 7 6 5 4 3 2 1 0 ppm
1.02 0.97 0.98 0.97 1.00 0.97 1.02 3.96 1.04 2.00 3.94 2.93 5.97
H3C
H
N
H
N
N
S
N
N
5l

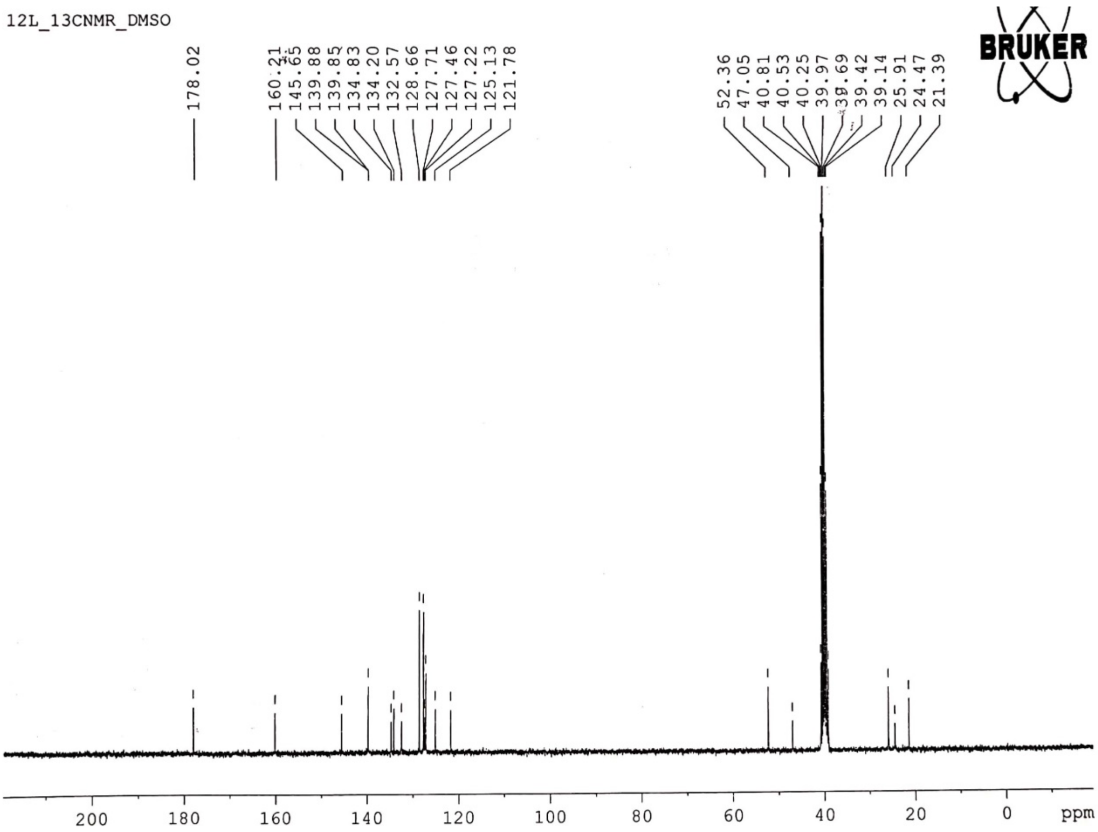
12L_13CNMR_DMSO
BRUKER
178.02
160.21 145.65 139.88 139.85 134.83 134.20 132.57 128.66 127.71 127.46 127.22 125.13 121.78
52.36 47.05 40.81 40.53 40.25 39.97 39.69 39.42 39.14 25.91 24.47 21.39
200 180 160 140 120 100 80 60 40 20 0 ppm

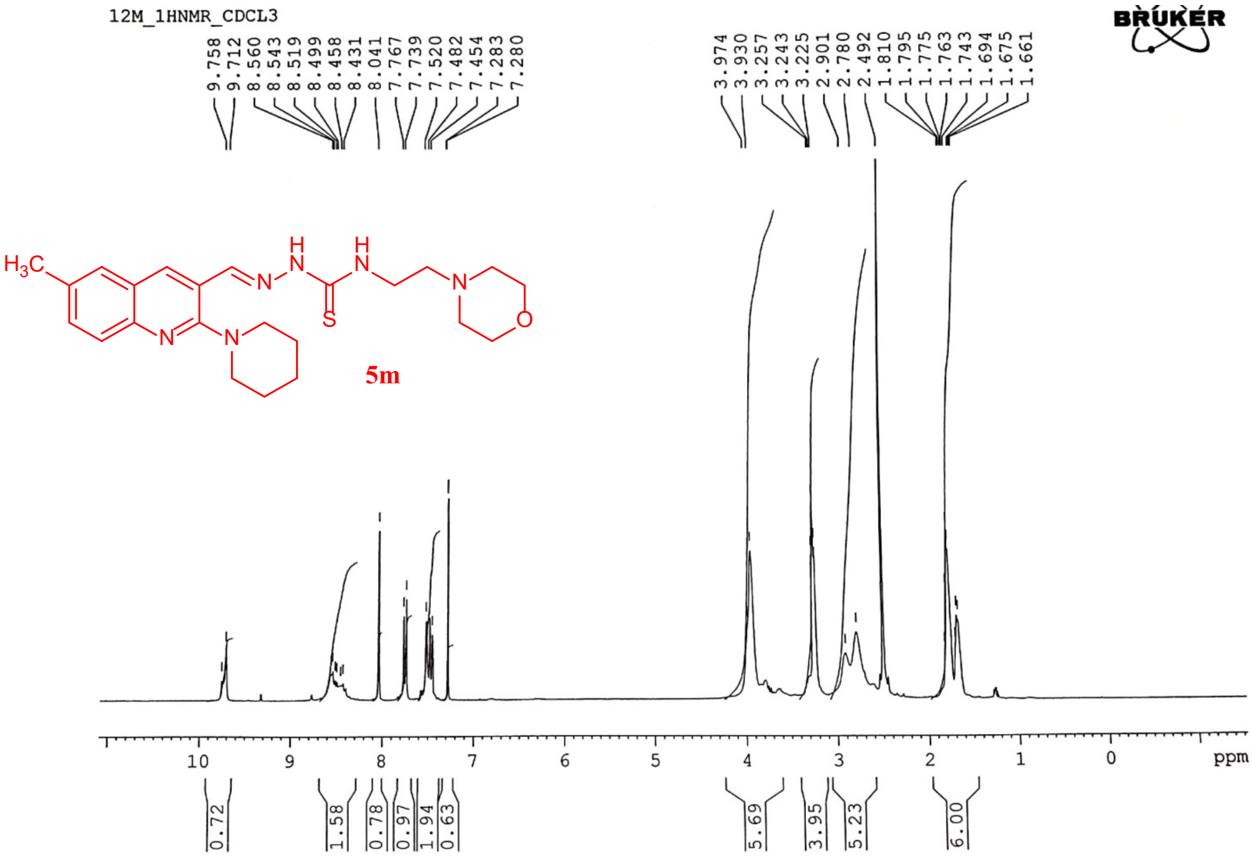

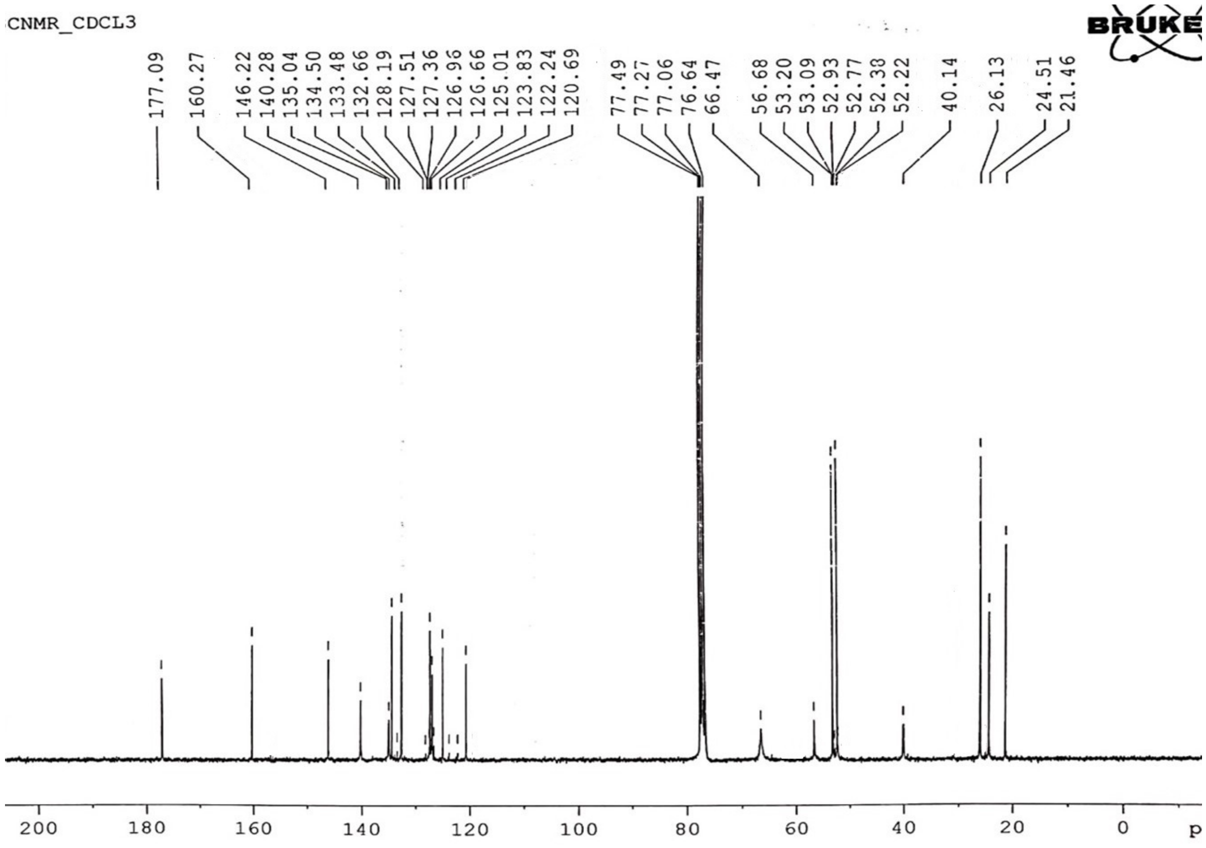

Figure S6. NMR Spectra of thiosemicarbazones 6(a-m).

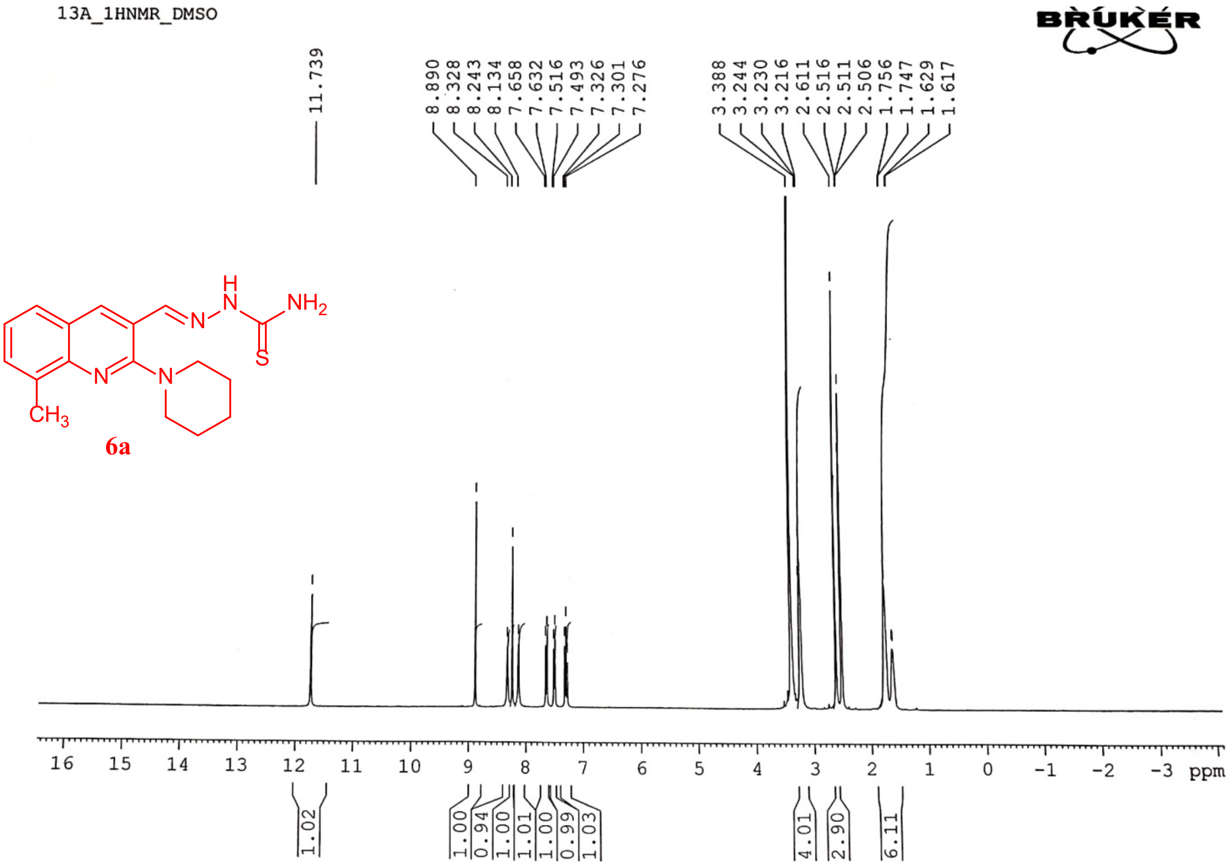
13A_1HNMR_DMSO
BRUKER
11.739
8.890
8.328
8.243
8.134
7.658
7.632
7.516
7.493
7.326
7.301
7.276
3.388
3.244
3.230
3.216
2.611
2.516
2.511
2.506
1.756
1.747
1.629
1.617
NH2
S
CH3
6a
16 15 14 13 12 11 10 9 8 7 6 5 4 3 2 1 0 -1 -2 -3 ppm
1.02
1.00
0.94
1.00
1.01
1.00
0.99
1.03
4.01
2.90
6.11

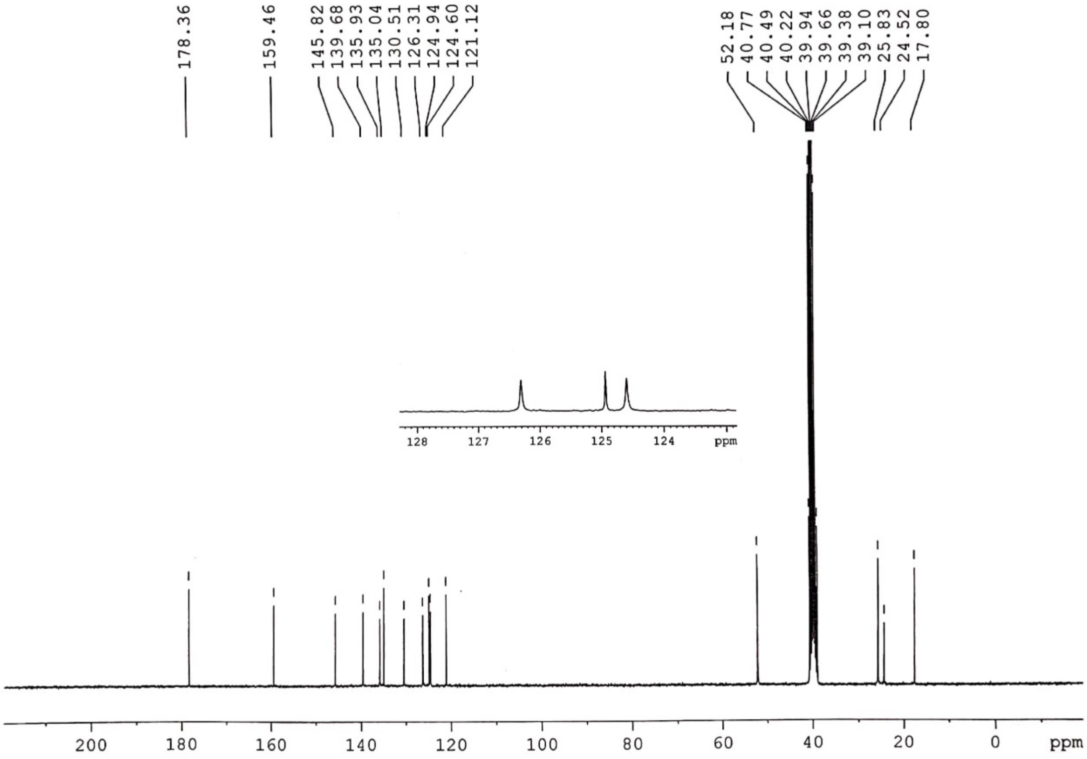
178.36
159.46
145.82
139.68
135.93
135.04
130.51
126.31
124.94
124.60
121.12
52.18
40.77
40.49
40.22
39.94
39.66
39.38
39.10
25.83
24.52
17.80
128 127 126 125 124 ppm
200 180 160 140 120 100 80 60 40 20 0 ppm

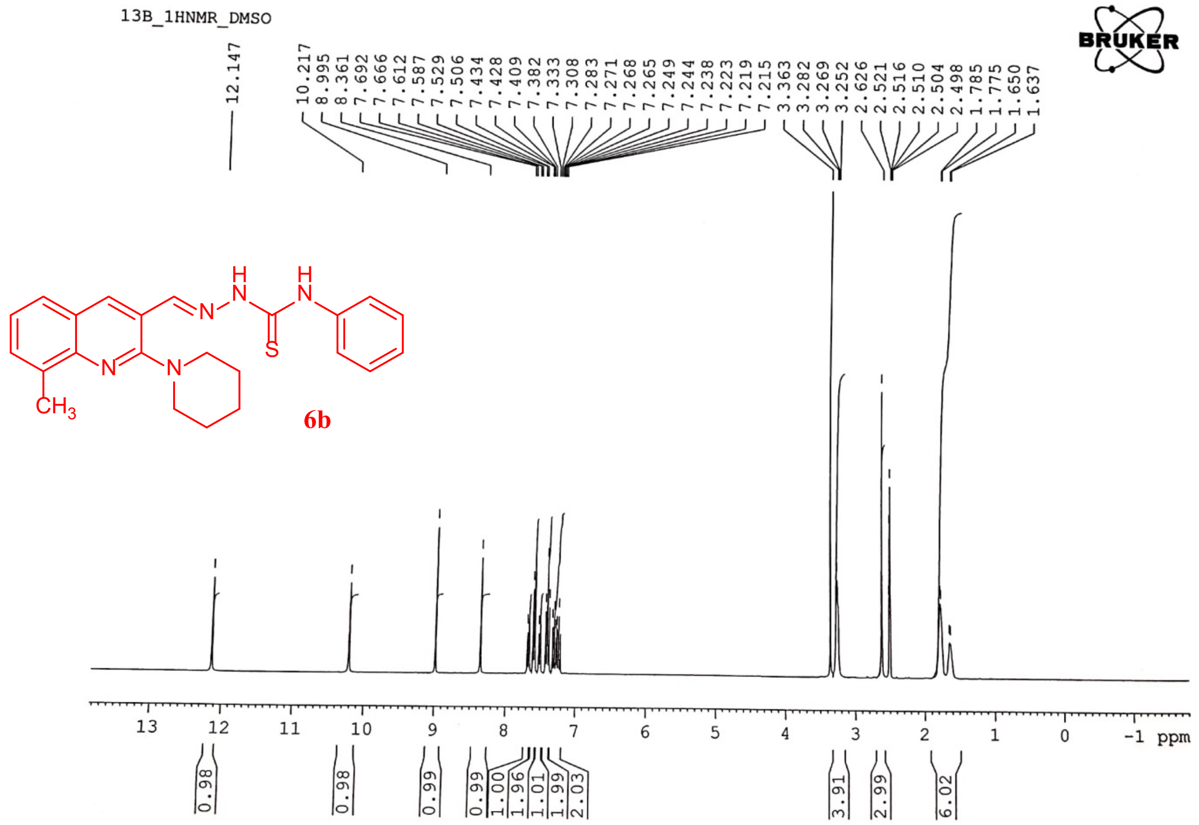
13B_1HNMR_DMSO
12.147
10.217 8.995 8.361 7.692 7.666 7.612 7.587 7.529 7.506 7.434 7.428 7.409 7.382 7.333 7.308 7.283 7.271 7.268 7.265 7.249 7.244 7.238 7.223 7.219 7.215
3.363 3.282 3.269 3.252 2.626 2.521 2.516 2.510 2.504 2.498 1.785 1.775 1.650 1.637
BRUKER
6b
0.98 0.98 0.99 0.99 1.00 1.96 1.01 1.99 2.03 3.91 2.99 6.02
ppm

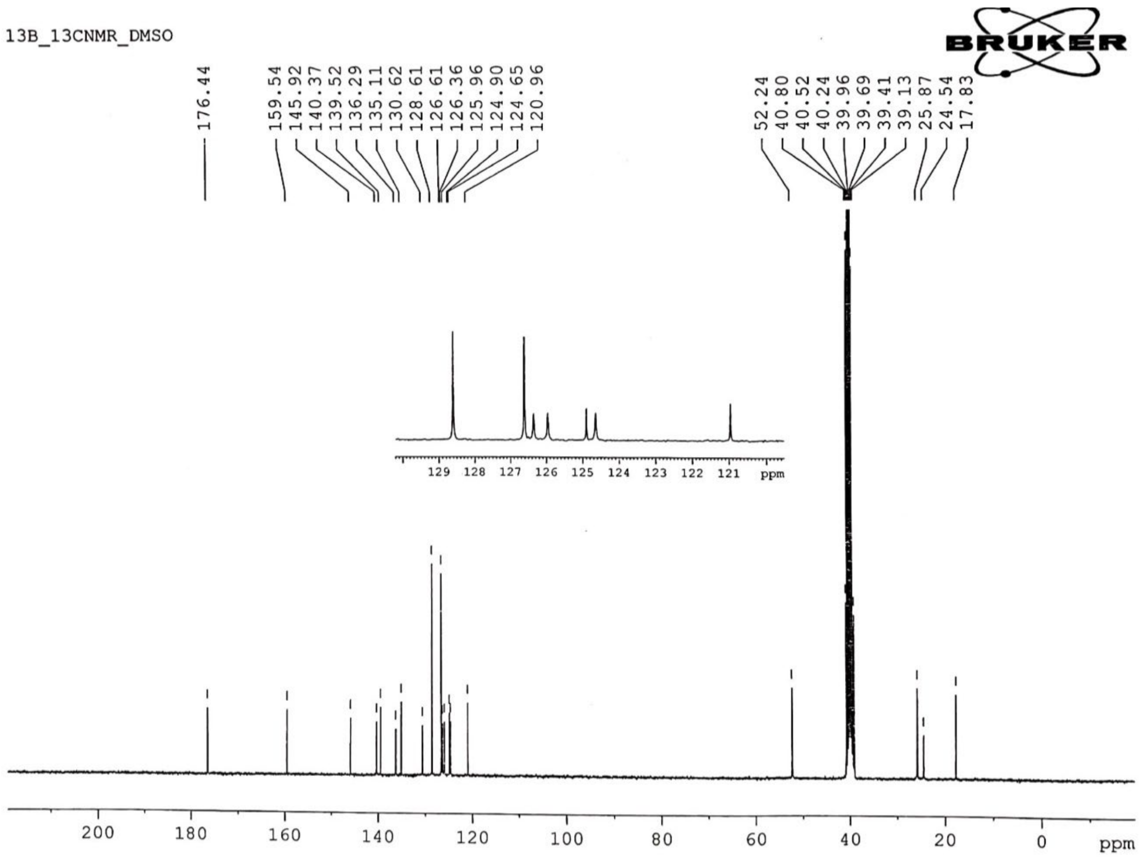
13B_13CNMR_DMSO
176.44
159.54 145.92 140.37 139.52 136.29 135.11 130.62 128.61 126.61 126.36 125.96 124.90 124.65 120.96
52.24 40.80 40.52 40.24 39.96 39.69 39.41 39.13 25.87 24.54 17.83
BRUKER
ppm

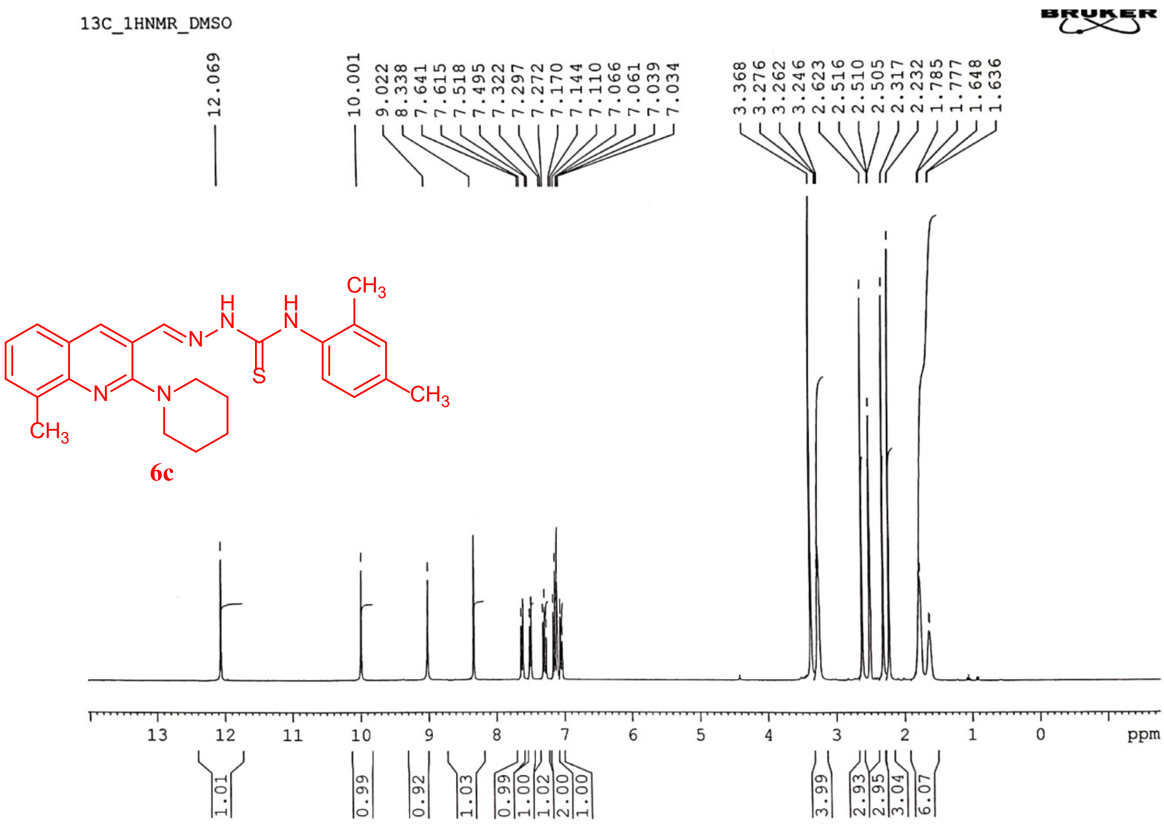

13C_1HNMR_DMSO
BRUKER
12.069
10.001
9.022
8.338
7.641
7.615
7.518
7.495
7.322
7.297
7.272
7.170
7.144
7.110
7.066
7.061
7.039
7.034
3.368
3.276
3.262
3.246
2.623
2.516
2.510
2.505
2.317
2.232
1.785
1.777
1.648
1.636
6c
ppm
1.01
0.99
0.92
1.03
0.99
1.00
1.02
2.00
1.00
3.99
2.93
2.95
3.04
6.07

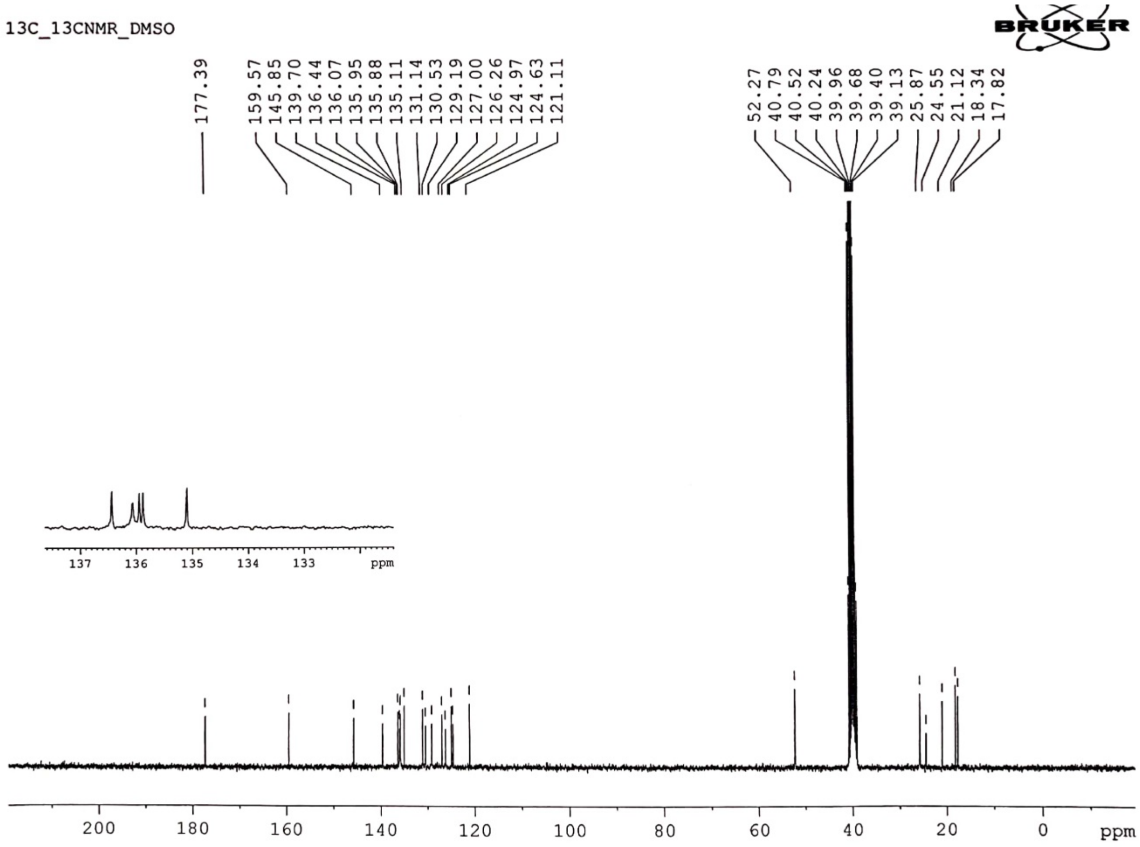

13C_13CNMR_DMSO
BRUKER
177.39
159.57
145.85
139.70
136.44
136.07
135.95
135.88
135.11
131.14
130.53
129.19
127.00
126.26
124.97
124.63
121.11
52.27
40.79
40.52
40.24
39.96
39.68
39.40
39.13
25.87
24.55
21.12
18.34
17.82
ppm

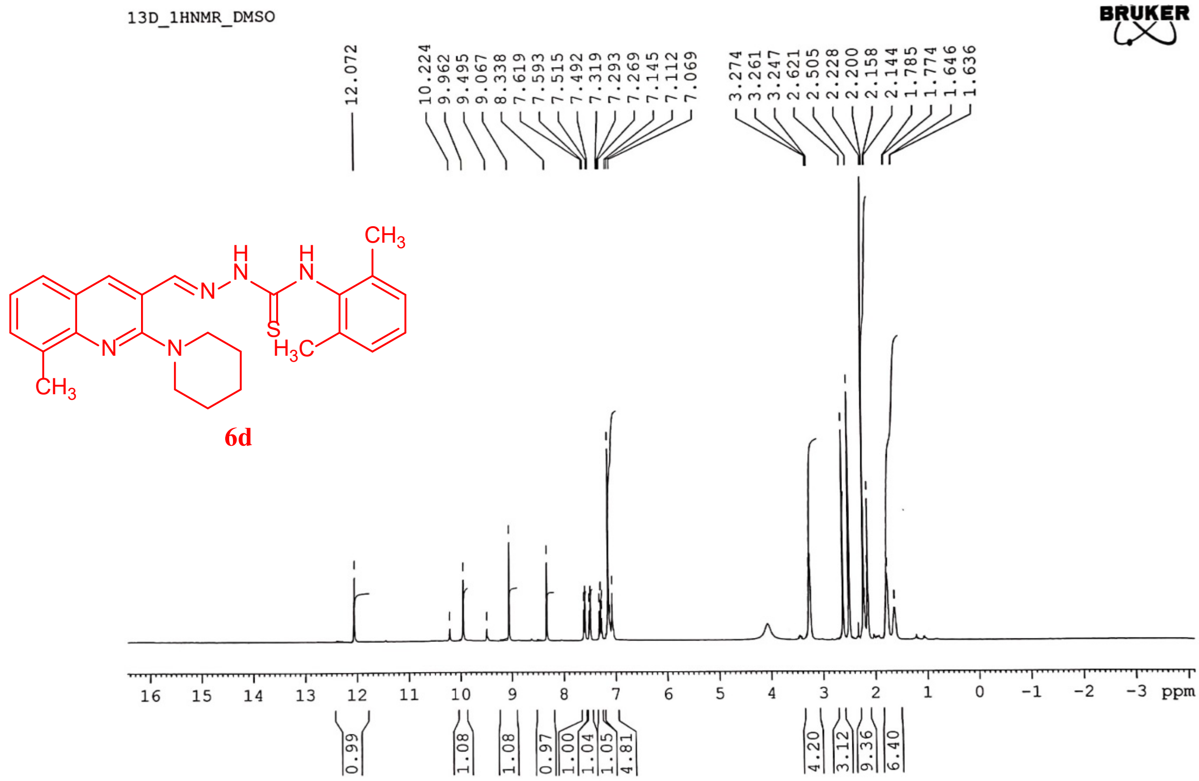
13D_1HNMR_DMSO
BRUKER
12.072
10.224
9.962
9.495
9.067
8.338
7.619
7.593
7.515
7.492
7.319
7.293
7.269
7.145
7.112
7.069
3.274
3.261
3.247
2.621
2.505
2.228
2.200
2.158
2.144
1.785
1.774
1.646
1.636
CH_3
H
N
H
N
N
S
H_3C
N
N
CH_3
6d
16 15 14 13 12 11 10 9 8 7 6 5 4 3 2 1 0 -1 -2 -3 ppm
0.99
1.08
1.08
0.97
1.00
1.04
1.05
4.81
4.20
3.12
9.36
6.40

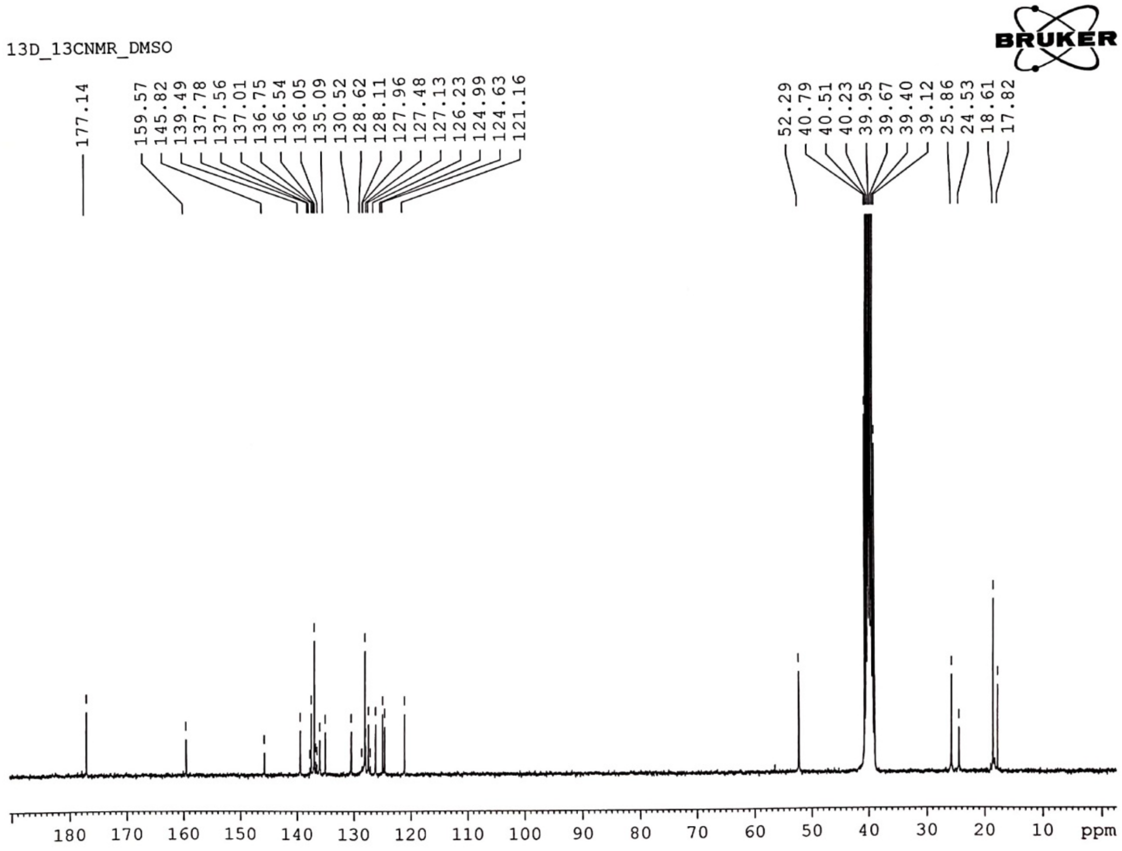
13D_13CNMR_DMSO
BRUKER
177.14
159.57
145.82
139.49
137.78
137.56
137.01
136.75
136.54
136.05
135.09
130.52
128.62
128.11
127.96
127.48
127.13
126.23
124.99
124.63
121.16
52.29
40.79
40.51
40.23
39.95
39.67
39.40
39.12
25.86
24.53
18.61
17.82
180 170 160 150 140 130 120 110 100 90 80 70 60 50 40 30 20 10 ppm

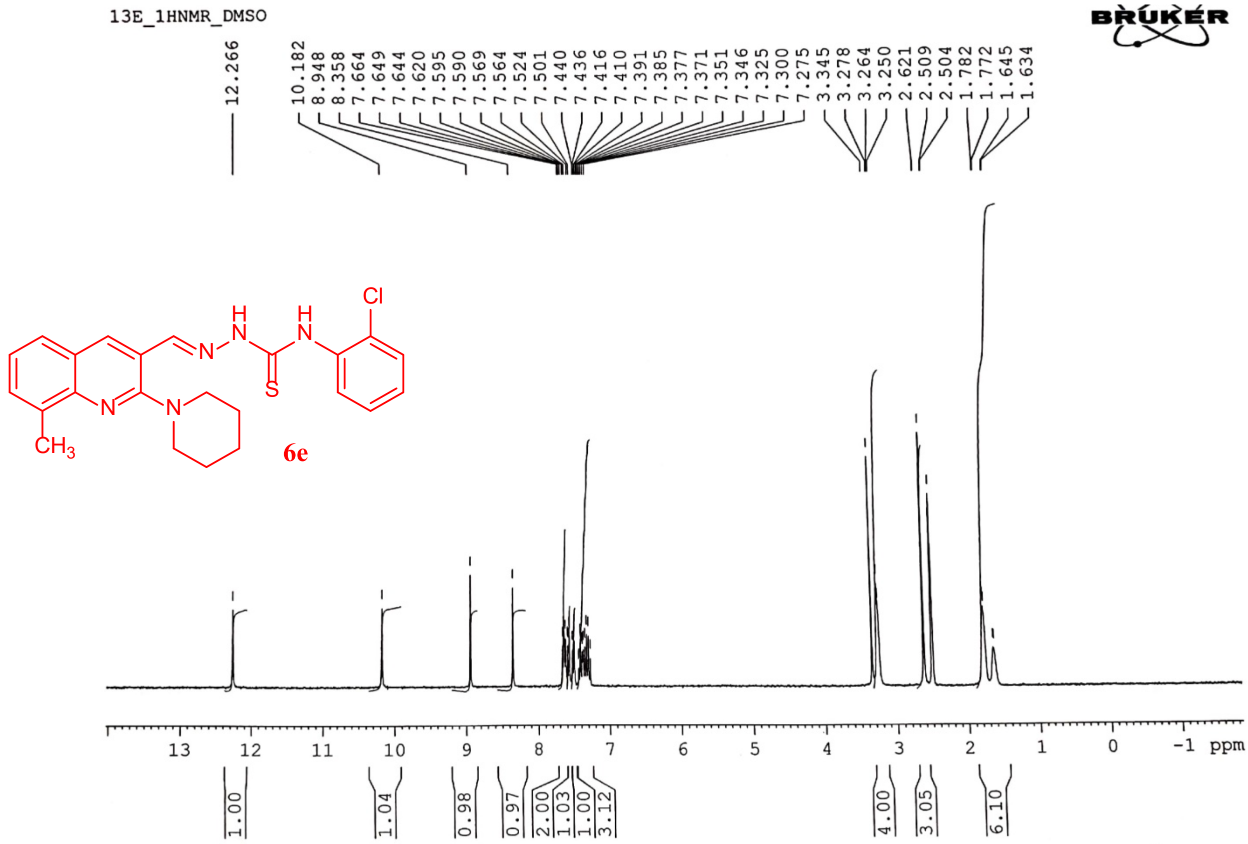
13E_1HNMR_DMSO
BRUKER
12.266
10.182
8.948
8.358
7.664
7.649
7.644
7.620
7.595
7.590
7.569
7.564
7.524
7.501
7.440
7.436
7.416
7.410
7.391
7.385
7.377
7.371
7.351
7.346
7.325
7.300
7.275
3.345
3.278
3.264
3.250
2.621
2.509
2.504
1.782
1.772
1.645
1.634
Cl
H
N
N
H
N
S
N
N
CH3
6e
13 12 11 10 9 8 7 6 5 4 3 2 1 0 -1 ppm
1.00
1.04
0.98
0.97
2.00
1.03
1.00
3.12
4.00
3.05
6.10

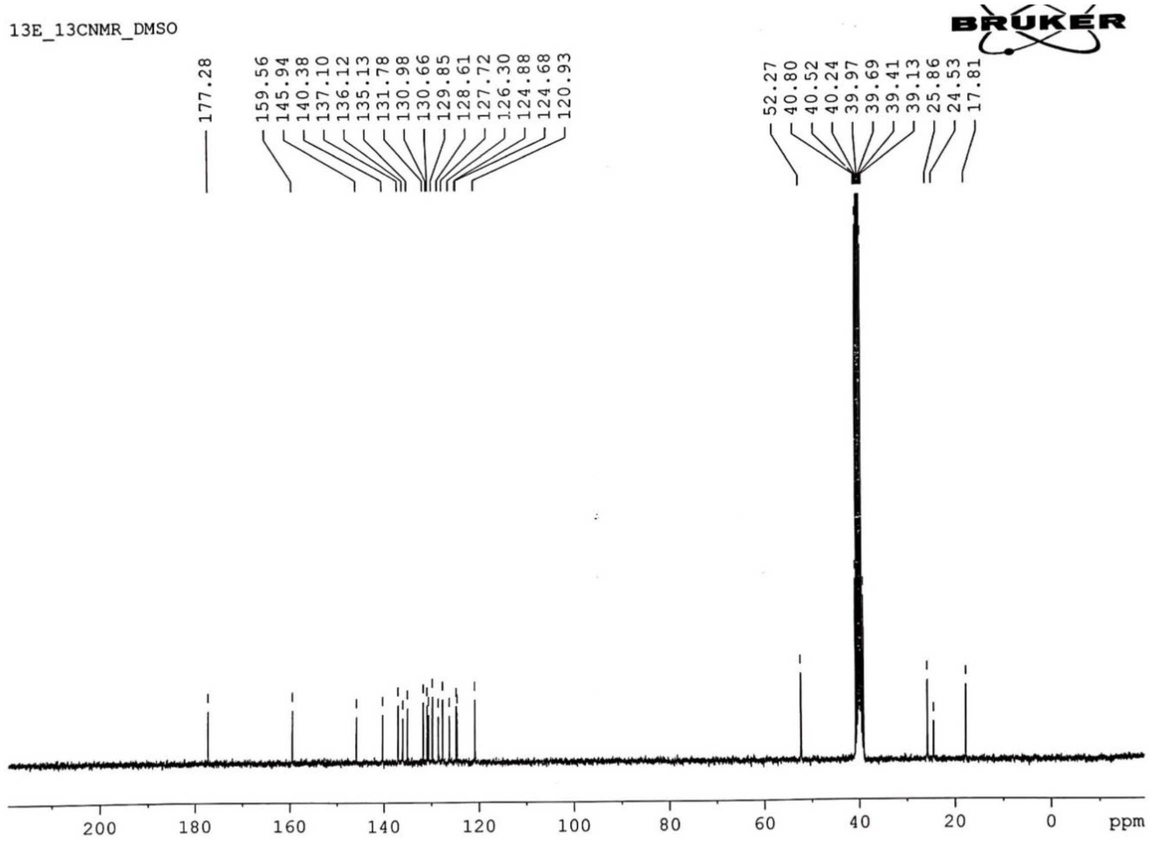
13E_13CNMR_DMSO
BRUKER
177.28
159.56
145.94
140.38
137.10
136.12
135.13
131.78
130.98
130.66
129.85
128.61
127.72
126.30
124.88
124.68
120.93
52.27
40.80
40.52
40.24
39.97
39.69
39.41
39.13
25.86
24.53
17.81
200 180 160 140 120 100 80 60 40 20 0 ppm

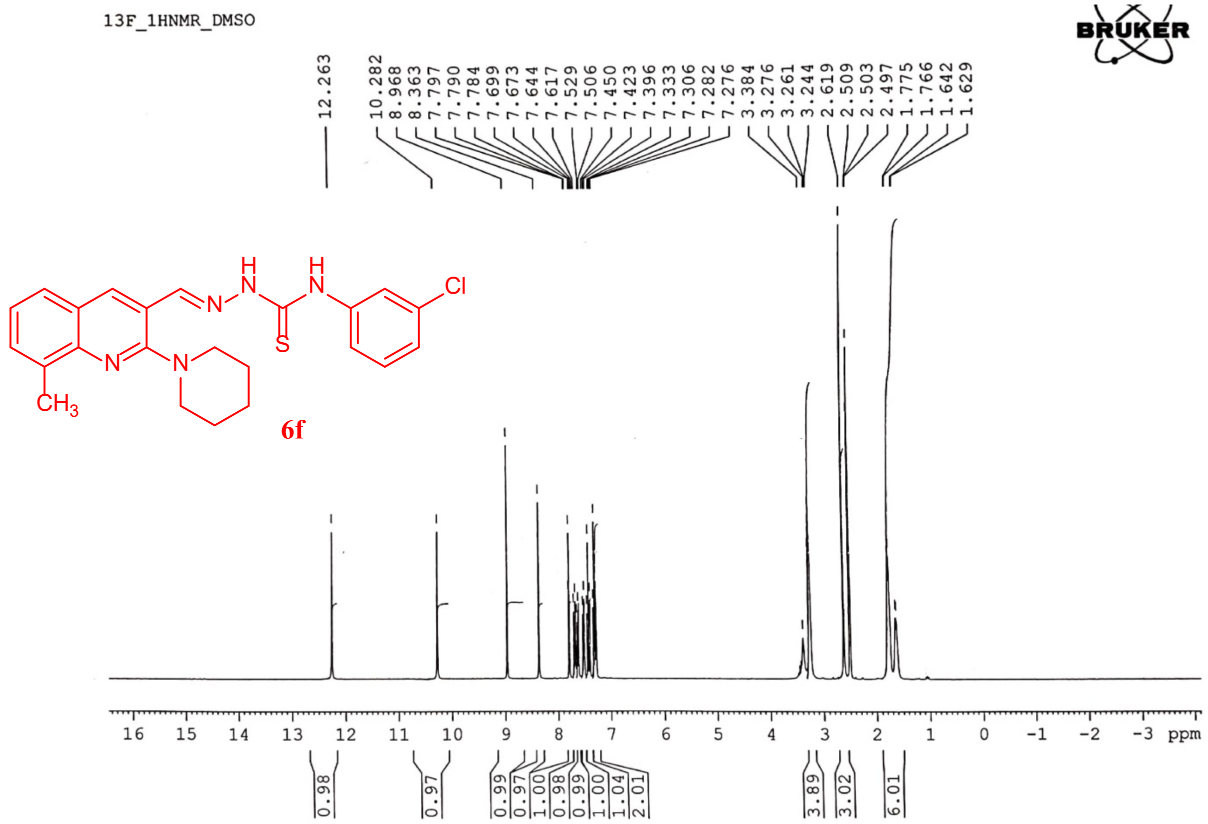
13F_1HNMR_DMSO
BRUKER
12.263
10.282
8.968
8.363
7.797
7.790
7.784
7.699
7.673
7.644
7.617
7.529
7.506
7.450
7.423
7.396
7.333
7.306
7.282
7.276
3.384
3.276
3.261
3.244
2.619
2.509
2.503
2.497
1.775
1.766
1.642
1.629
CH3
Cl
6f
ppm
0.98
0.97
0.99
0.97
1.00
0.98
0.99
1.00
1.04
2.01
3.89
3.02
6.01

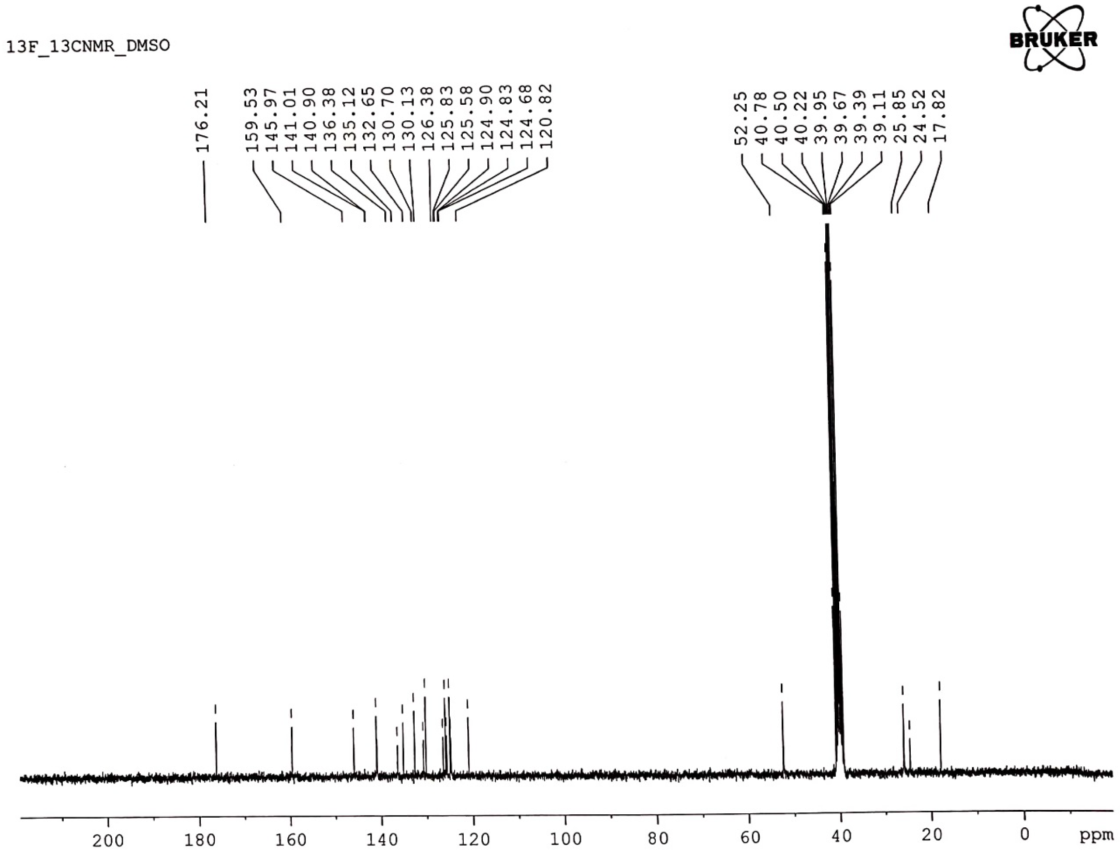
13F_13CNMR_DMSO
BRUKER
176.21
159.53
145.97
141.01
140.90
136.38
135.12
132.65
130.70
130.13
126.38
125.83
125.58
124.90
124.83
124.68
120.82
52.25
40.78
40.50
40.22
39.95
39.67
39.39
39.11
25.85
24.52
17.82
ppm

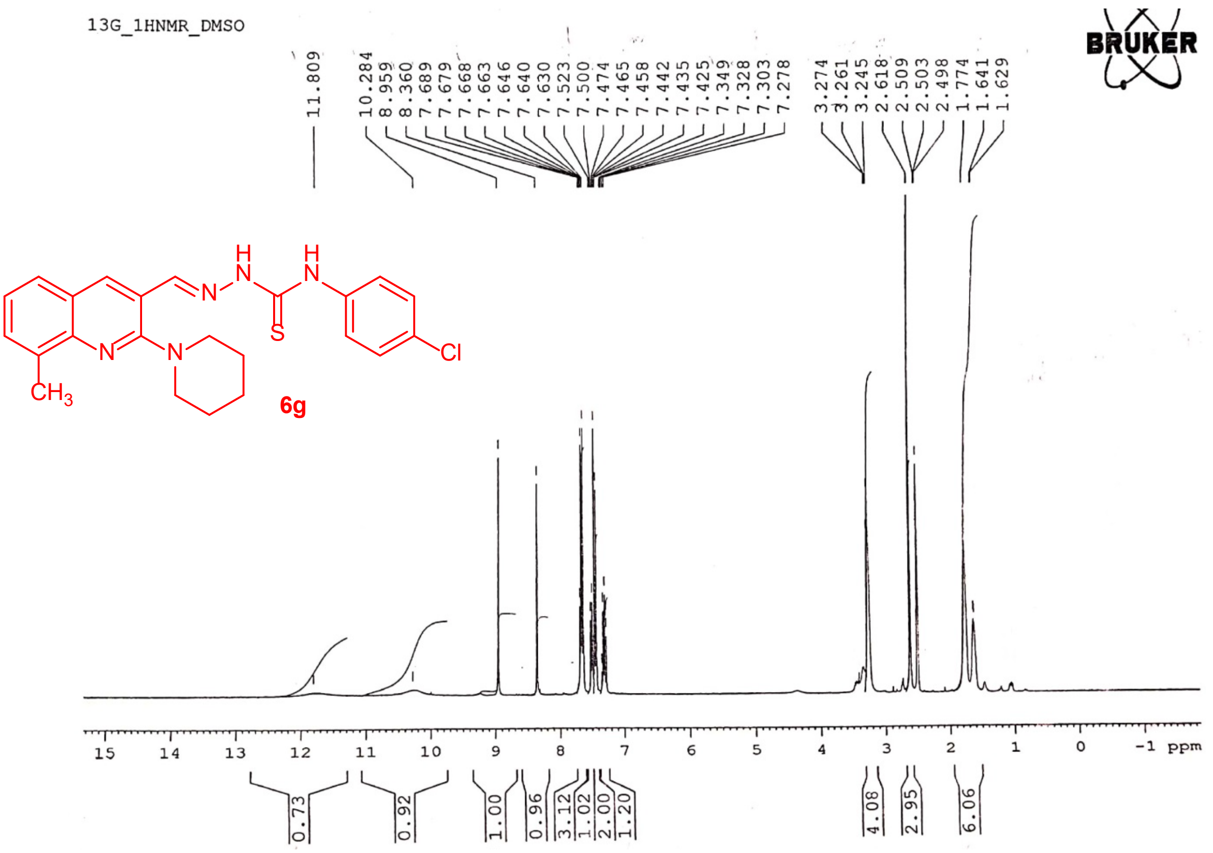
13G_1HNMR_DMSO
BRUKER
11.809
10.284 8.959 8.360 7.689 7.679 7.668 7.663 7.646 7.640 7.630 7.523 7.500 7.474 7.465 7.458 7.442 7.435 7.425 7.349 7.328 7.303 7.278
3.274 3.261 3.245 2.618 2.509 2.503 2.498 1.774 1.641 1.629
6g
Cl
S
N
H
CH3
15 14 13 12 11 10 9 8 7 6 5 4 3 2 1 0 -1 ppm
0.73 0.92 1.00 0.96 3.12 1.02 2.00 1.20 4.08 2.95 6.06

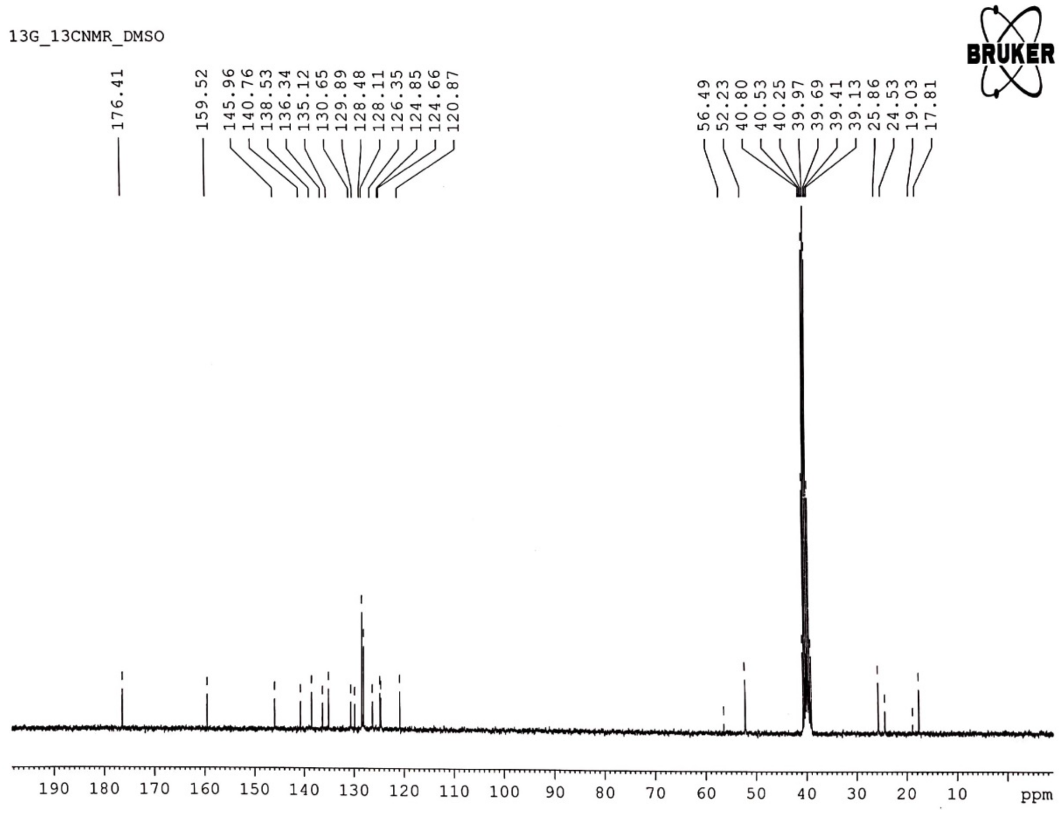
13G_13CNMR_DMSO
BRUKER
176.41
159.52 145.96 140.76 138.53 136.34 135.12 130.65 129.89 128.48 128.11 126.35 124.85 124.66 120.87
56.49 52.23 40.80 40.53 40.25 39.97 39.69 39.41 39.13 25.86 24.53 19.03 17.81
190 180 170 160 150 140 130 120 110 100 90 80 70 60 50 40 30 20 10 ppm

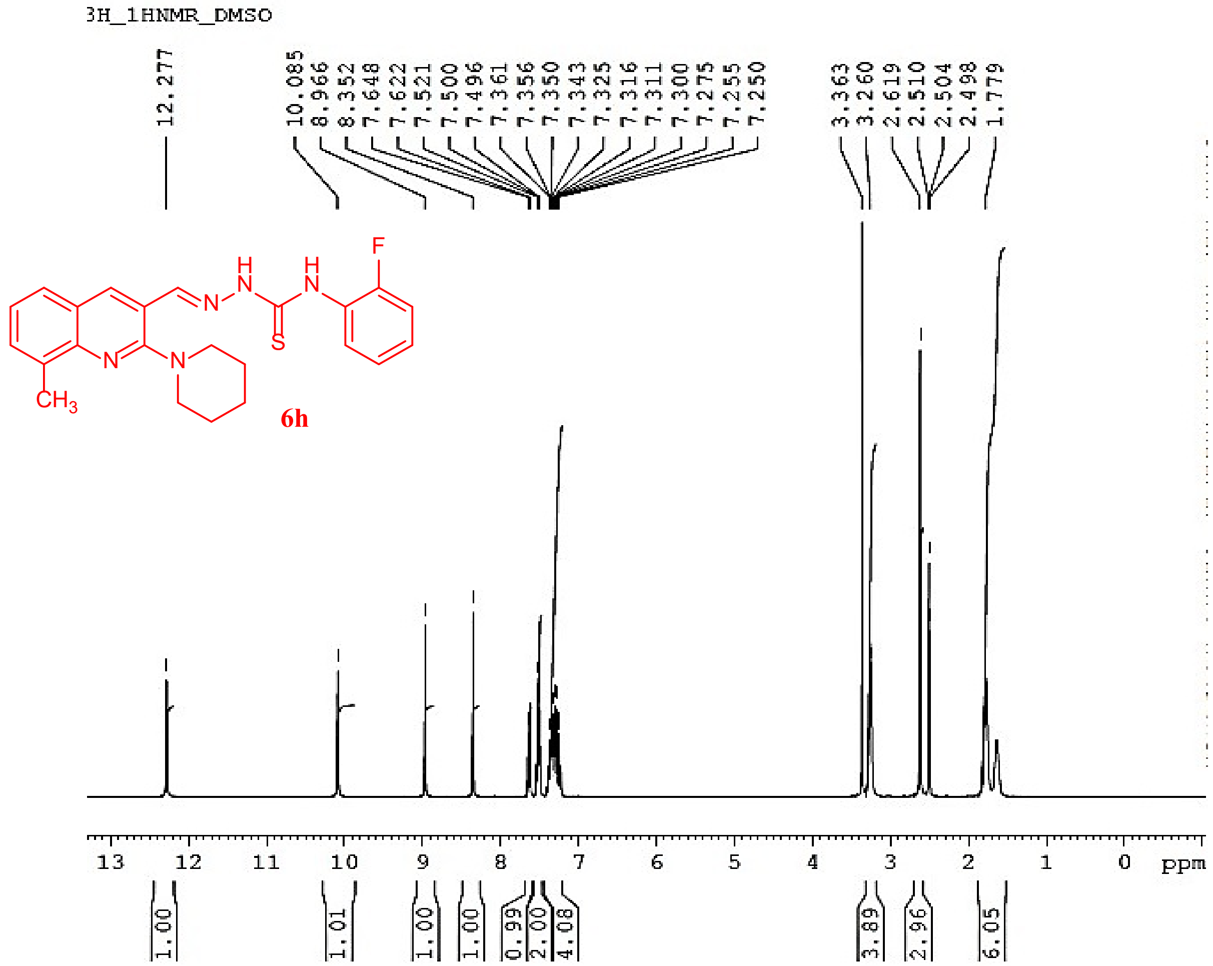
H_1HNMR_DMSO
12.277
10.085
8.966
8.352
7.648
7.622
7.521
7.500
7.496
7.361
7.356
7.350
7.343
7.325
7.316
7.311
7.300
7.275
7.255
7.250
3.363
3.260
2.619
2.510
2.504
2.498
1.779
F
H
N
N
H
N
S
N
N
CH3
6h
13 12 11 10 9 8 7 6 5 4 3 2 1 0 ppm
1.00
1.01
1.00
1.00
0.99
2.00
4.08
3.89
2.96
6.05

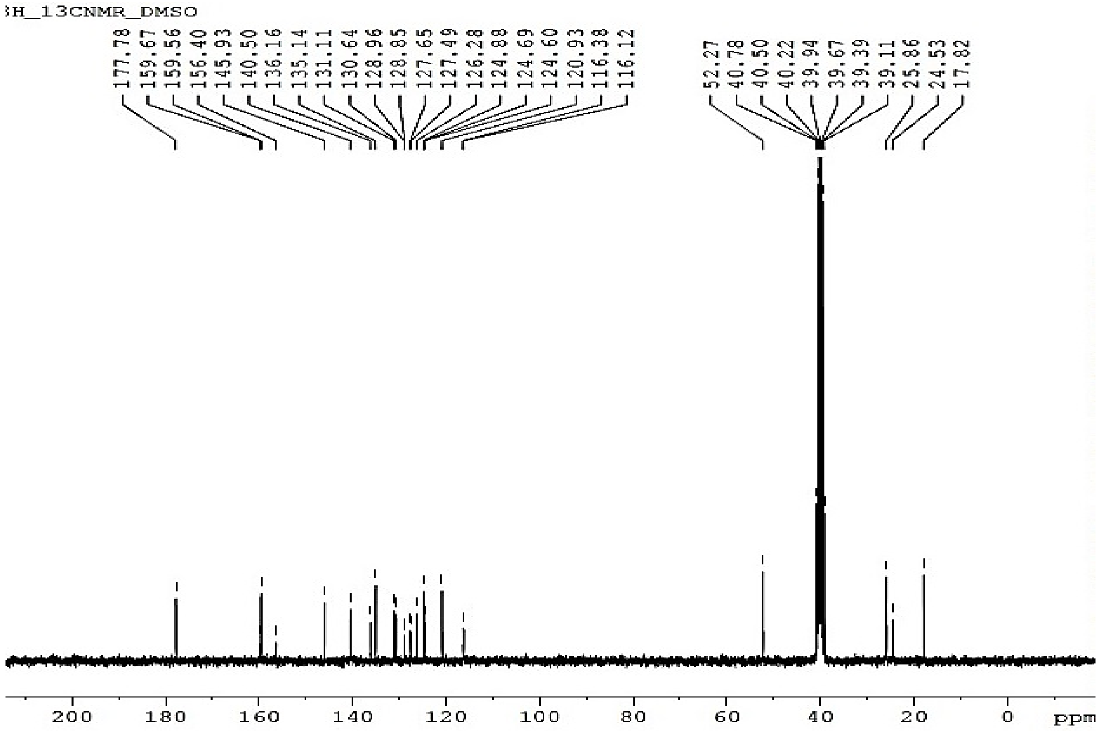
H_13CNMR_DMSO
177.78
159.67
159.56
156.40
145.93
140.50
136.16
135.14
131.11
130.64
128.96
128.85
127.65
127.49
126.28
124.88
124.69
124.60
120.93
116.38
116.12
52.27
40.78
40.50
40.22
39.94
39.67
39.39
39.11
25.86
24.53
17.82
200 180 160 140 120 100 80 60 40 20 0 ppm

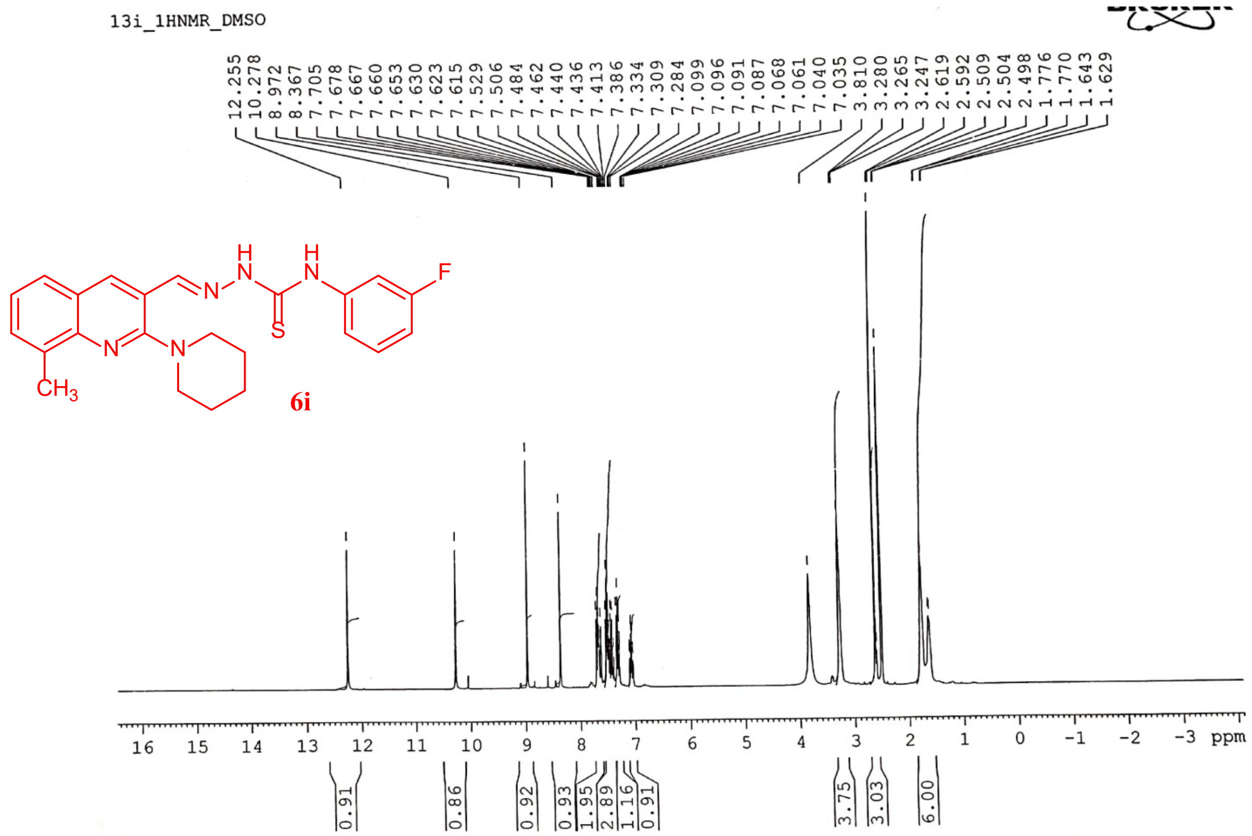
13i_1HNMR_DMSO
12.255
10.278
8.972
8.367
7.705
7.678
7.667
7.660
7.653
7.630
7.623
7.615
7.529
7.506
7.484
7.462
7.440
7.436
7.413
7.386
7.334
7.309
7.284
7.099
7.096
7.091
7.087
7.068
7.061
7.040
7.035
3.810
3.280
3.265
3.247
2.619
2.592
2.509
2.504
2.498
1.776
1.770
1.643
1.629
F
N
H
S
CH3
6i
16 15 14 13 12 11 10 9 8 7 6 5 4 3 2 1 0 -1 -2 -3 ppm
0.91
0.86
0.92
0.93
1.95
2.89
1.16
0.91
3.75
3.03
6.00

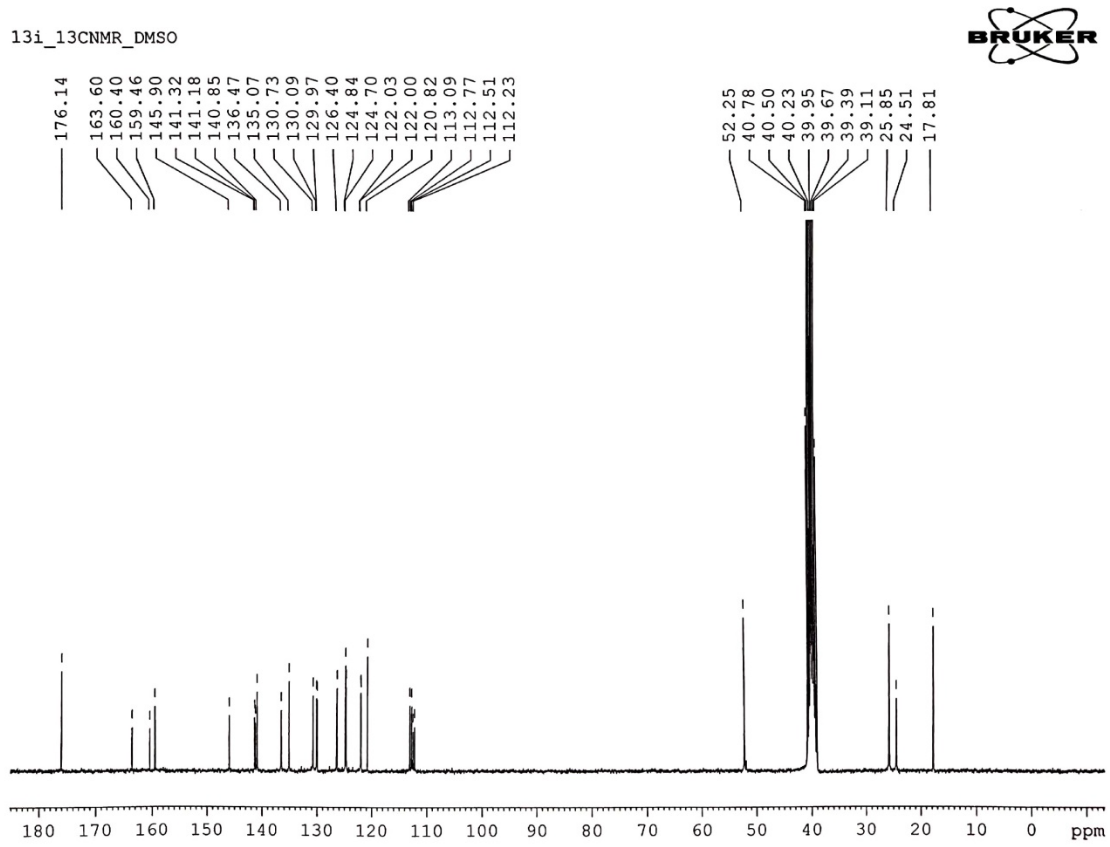
13i_13CNMR_DMSO
BRUKER
176.14
163.60
160.40
159.46
145.90
141.32
141.18
140.85
136.47
135.07
130.73
130.09
129.97
126.40
124.84
124.70
122.03
122.00
120.82
113.09
112.77
112.51
112.23
52.25
40.78
40.50
40.23
39.95
39.67
39.39
39.11
25.85
24.51
17.81
180 170 160 150 140 130 120 110 100 90 80 70 60 50 40 30 20 10 0 ppm

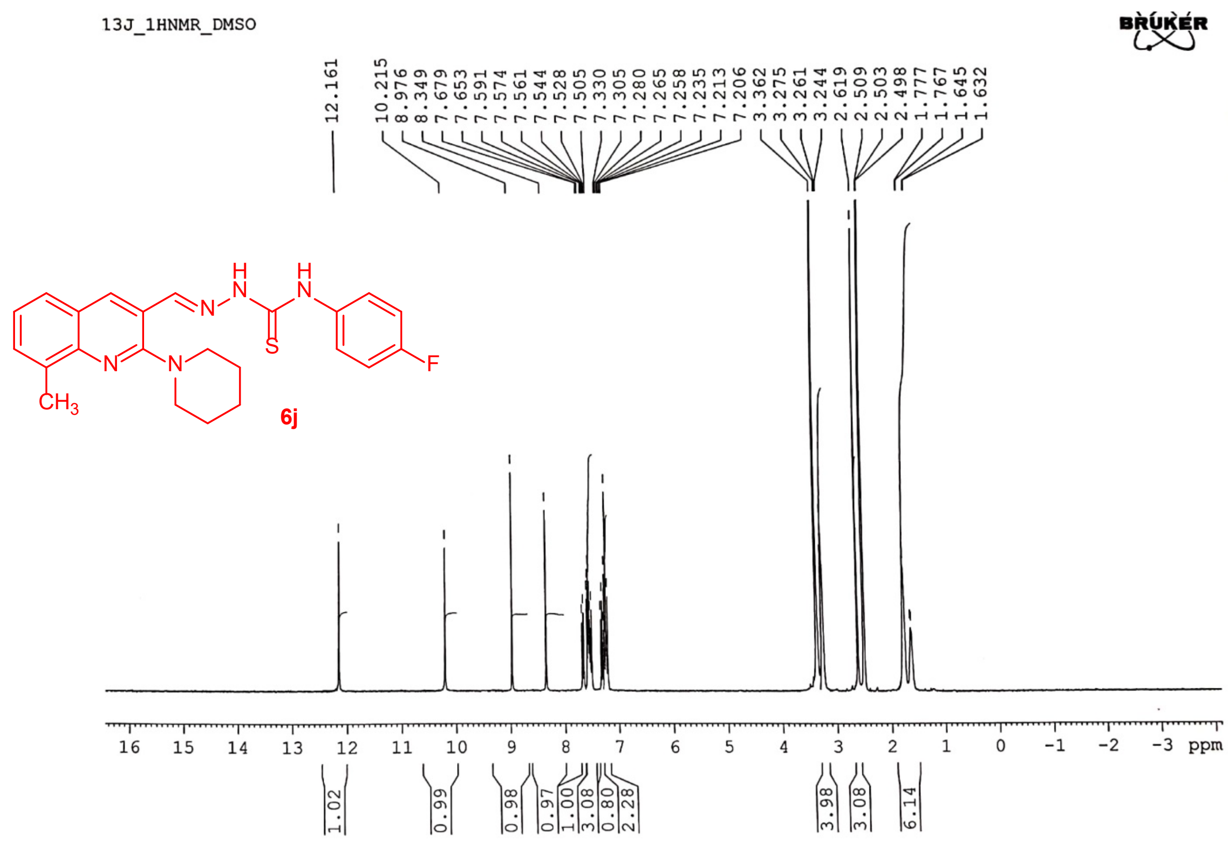
13J_1HNMR_DMSO
6j
ppm

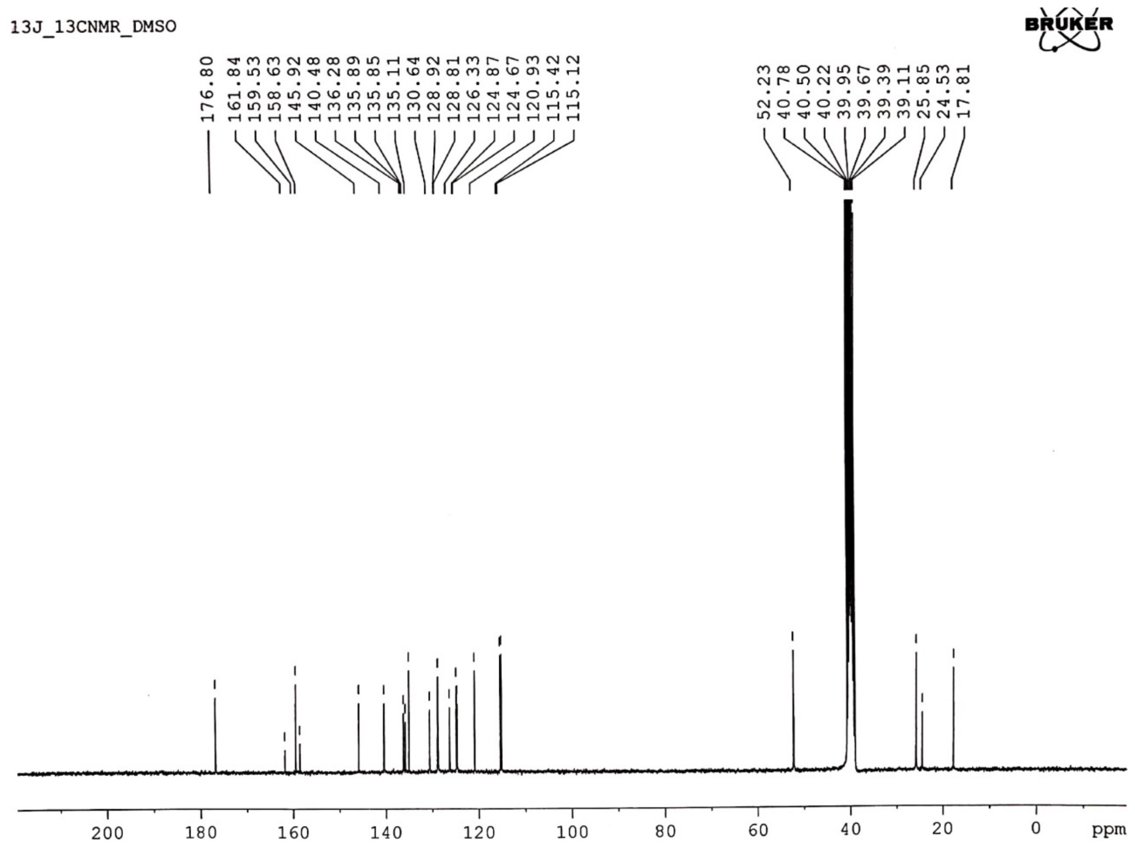
13J_13CNMR_DMSO
ppm

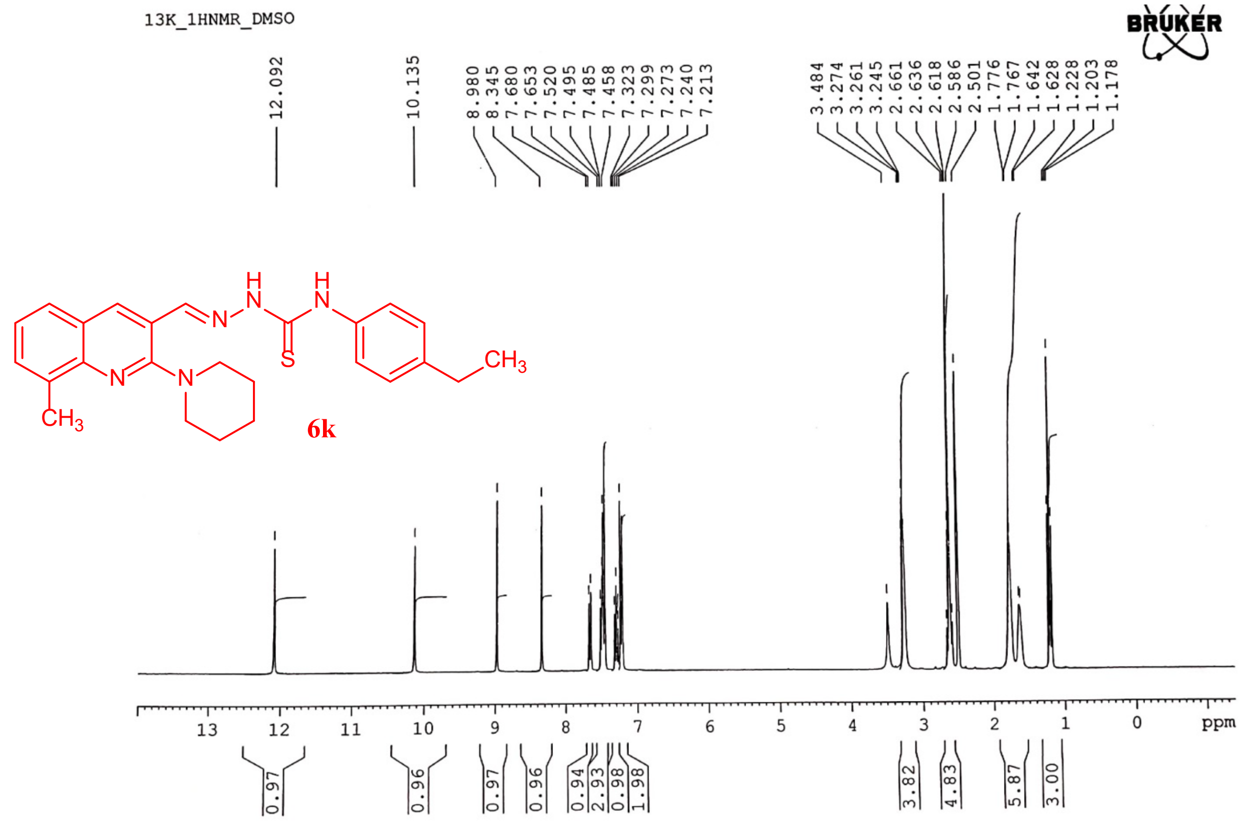
13K_1HNMR_DMSO
BRUKER
12.092
10.135
8.980
8.345
7.680
7.653
7.520
7.495
7.485
7.458
7.323
7.299
7.273
7.240
7.213
3.484
3.274
3.261
3.245
2.661
2.636
2.618
2.586
2.501
1.776
1.767
1.642
1.628
1.228
1.203
1.178
H
N
N
S
CH3
CH3
6k
13
12
11
10
9
8
7
6
5
4
3
2
1
0
ppm
0.97
0.96
0.97
0.96
0.94
2.93
0.98
1.98
3.82
4.83
5.87
3.00

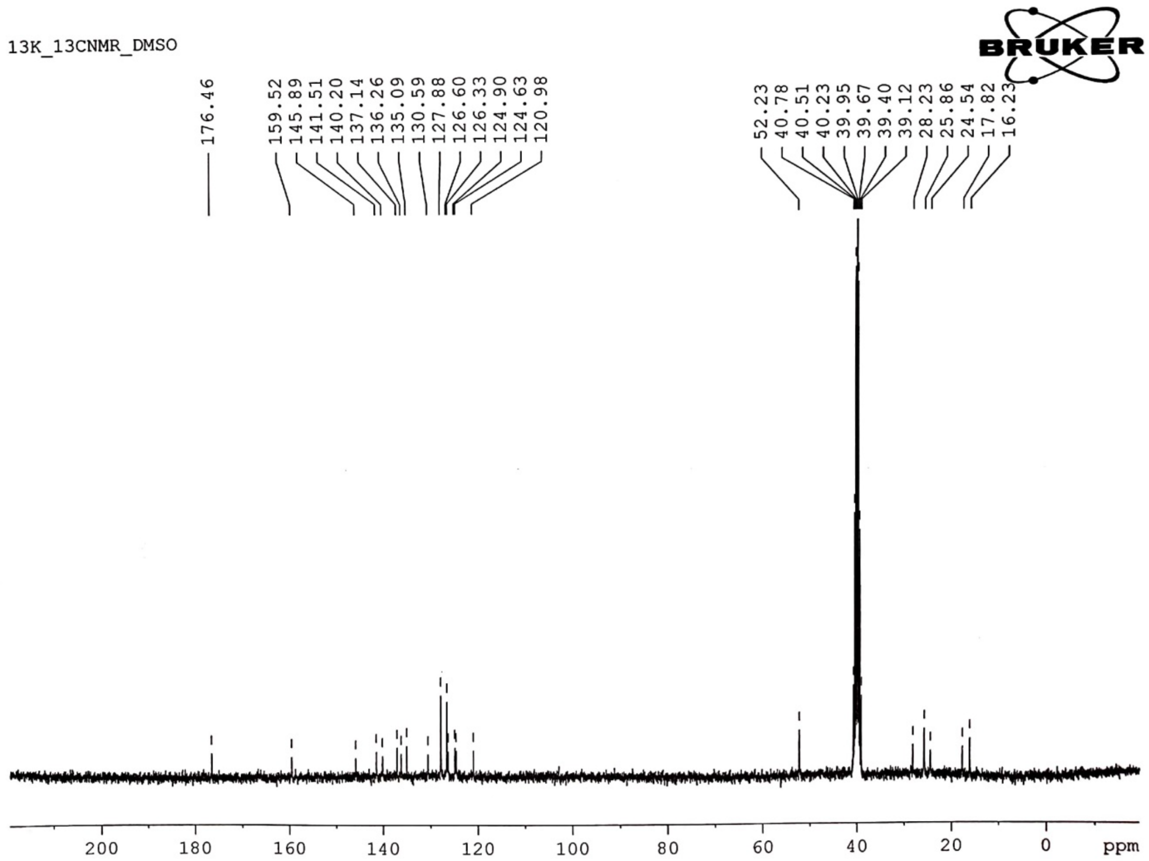
13K_13CNMR_DMSO
BRUKER
176.46
159.52
145.89
141.51
140.20
137.14
136.26
135.09
130.59
127.88
126.60
126.33
124.90
124.63
120.98
52.23
40.78
40.51
40.23
39.95
39.67
39.40
39.12
28.23
25.86
24.54
17.82
16.23
200
180
160
140
120
100
80
60
40
20
0
ppm

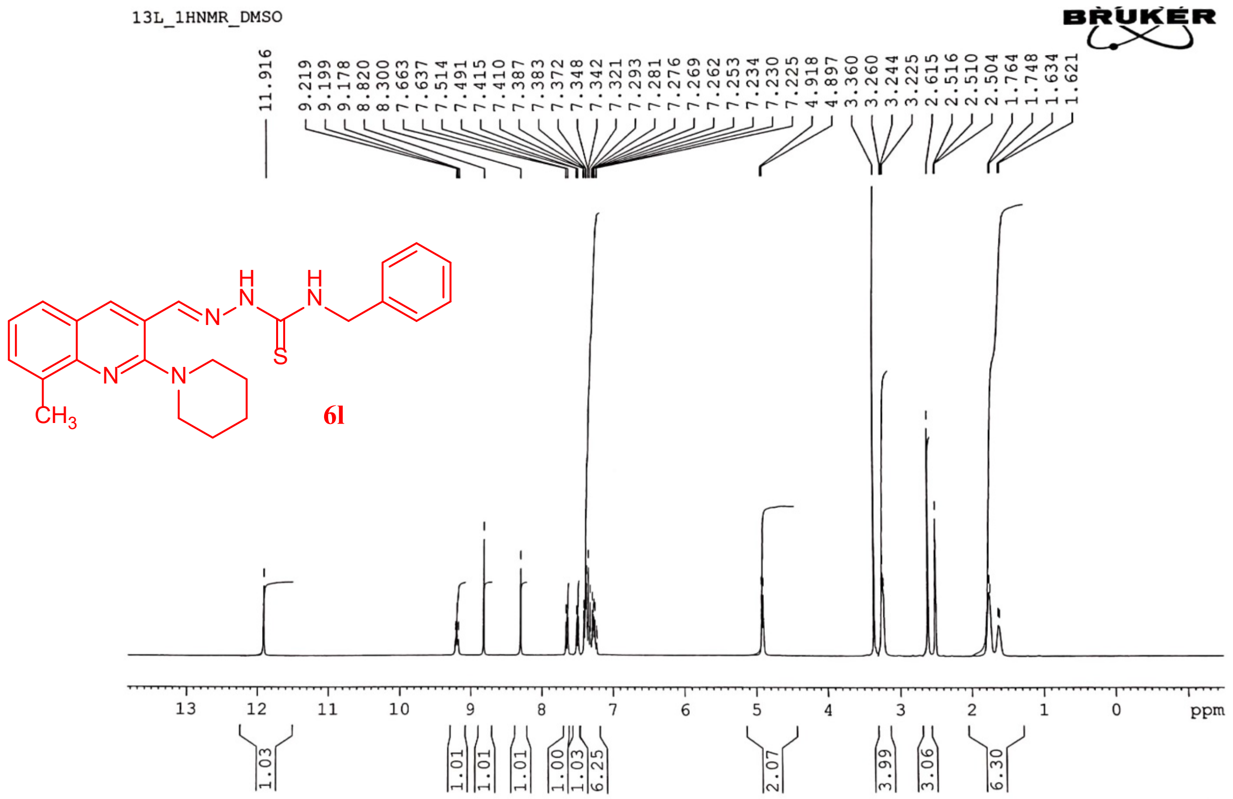
13L_1HNMR_DMSO
11.916
9.219
9.199
9.178
8.820
8.300
7.663
7.637
7.514
7.491
7.415
7.410
7.387
7.383
7.372
7.348
7.342
7.321
7.293
7.281
7.276
7.269
7.262
7.253
7.234
7.230
7.225
4.918
4.897
3.360
3.260
3.244
3.225
2.615
2.516
2.510
2.504
1.764
1.748
1.634
1.621
BRUKER
CH3
6l
13 12 11 10 9 8 7 6 5 4 3 2 1 0 ppm
1.03
1.01
1.01
1.01
1.00
1.03
6.25
2.07
3.99
3.06
6.30

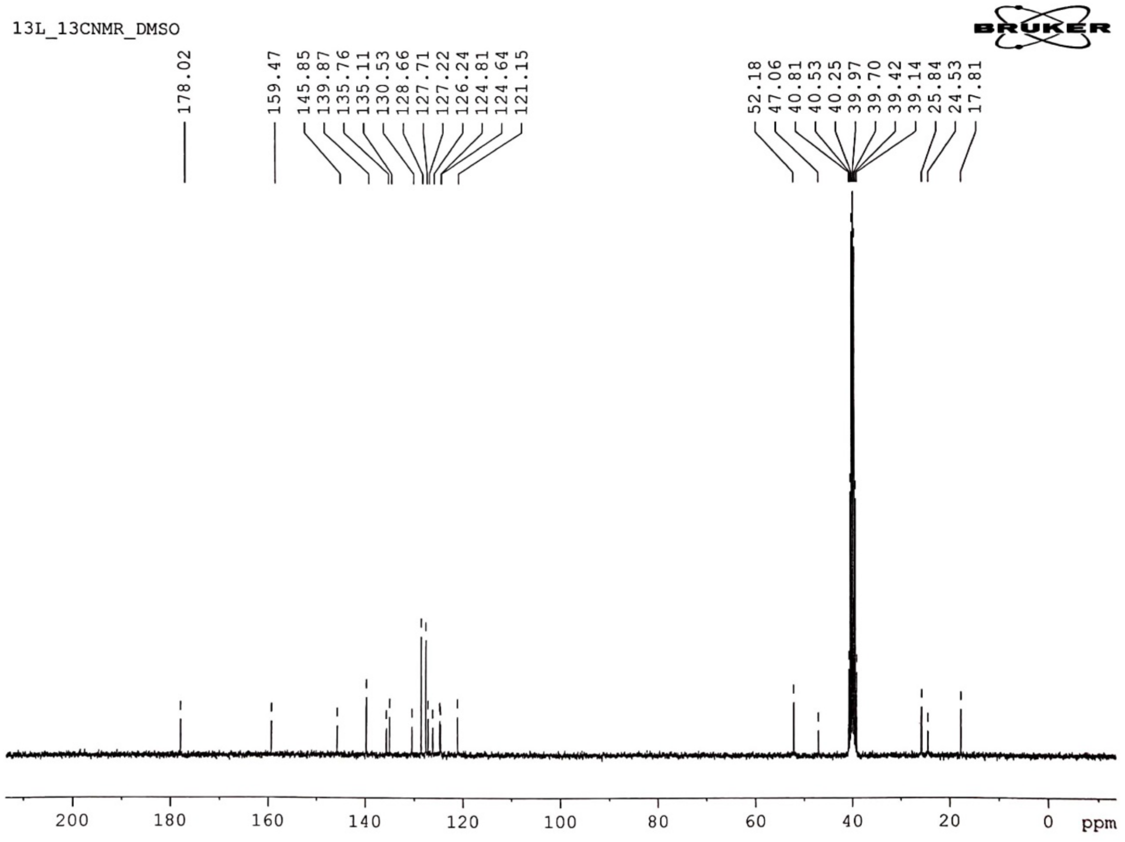
13L_13CNMR_DMSO
178.02
159.47
145.85
139.87
135.76
135.11
130.53
128.66
127.71
127.22
126.24
124.81
124.64
121.15
52.18
47.06
40.81
40.53
40.25
39.97
39.70
39.42
39.14
25.84
24.53
17.81
BRUKER
200 180 160 140 120 100 80 60 40 20 0 ppm

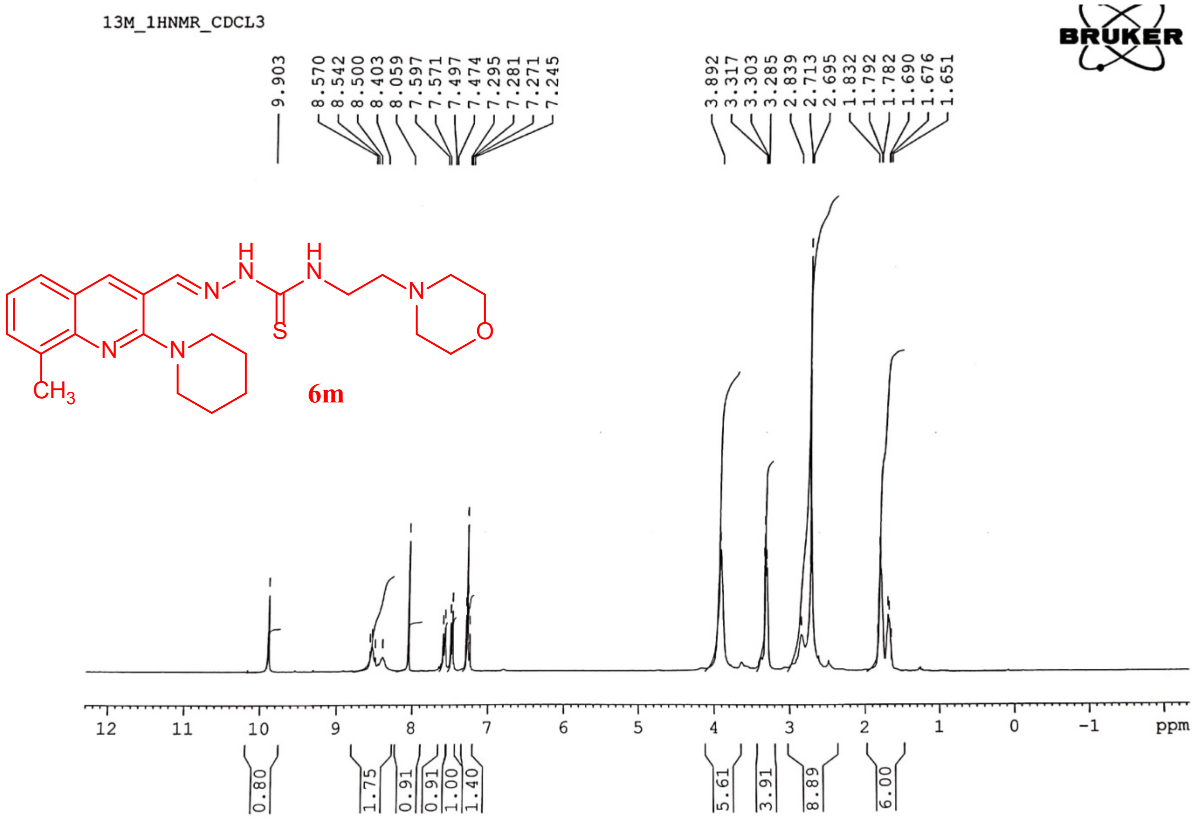
13M_1HNMR_CDCL3
BRUKER
9.903
8.570
8.542
8.500
8.403
8.059
7.597
7.571
7.497
7.474
7.295
7.281
7.271
7.245
3.892
3.317
3.303
3.285
2.839
2.713
2.695
1.832
1.792
1.782
1.690
1.676
1.651
6m
12 11 10 9 8 7 6 5 4 3 2 1 0 -1 ppm
0.80
1.75
0.91
0.91
1.00
1.40
5.61
3.91
8.89
6.00

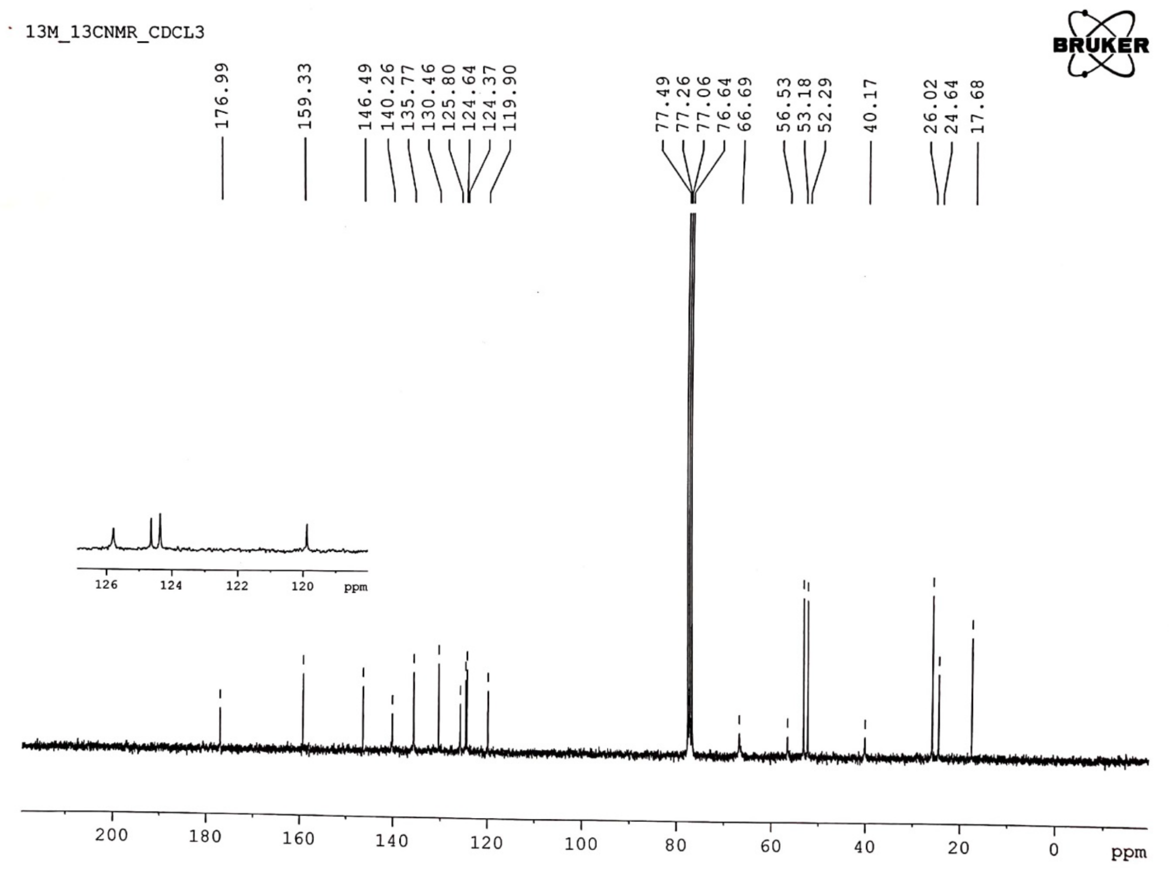
13M_13CNMR_CDCL3
176.99
159.33
146.49
140.26
135.77
130.46
125.80
124.64
124.37
119.90
77.49
77.26
77.06
76.64
66.69
56.53
53.18
52.29
40.17
26.02
24.64
17.68
126 124 122 120 ppm
200 180 160 140 120 100 80 60 40 20 0 ppm

References

1. Meth-Cohn, O.; Narine, B.; Tarnowski, B. A versatile new synthesis of quinolines and related fused pyridines, Part 5. The synthesis of 2-chloroquinoline-3-carbaldehydes. *J. Chem. Soc. Perkin Trans. 1*, **1981**, 1520–1530. https://doi.org/10.1039/P19810001537
2. Munir, R.; Athar, M.M.; Rehman, M.Z.; Javid, N. Synthesis of 6/8-methyl-2-(piperidin-1-yl)quinoline-3-carbaldehydes; A facile CTAB catalyzed protocol. Chiang Mai *J. Sci. 2020*, *47*, 175–180. http://epg.science.cmu.ac.th/ejournal/
3. Ramesh, V.; Rao, B.A.; Sharma, P.; Swarna, B.; Thummuri, D.; Srinivas, K.; Naidu, V.G.M.; Rao, V.J. Synthesis and biological evaluation of new rhodanine analogues bearing 2-chloroquinoline and benzo[h]quinoline scaffolds as anticancer agents. Eur. J. *Med. Chem.* **2014**, *83*, 569–580. https://doi.org/10.1016/0006-2952(61)90145-9
